# Human Digestive Physiology and Evolutionary Diet: A Metabolomic Perspective on Carnivorous and Scavenger Adaptations

**DOI:** 10.3390/metabo15070453

**Published:** 2025-07-04

**Authors:** Vicente Javier Clemente-Suárez, Laura Redondo-Flórez, Ana Isabel Beltrán-Velasco, Rodrigo Yáñez-Sepúlveda, Alejandro Rubio-Zarapuz, Alexandra Martín-Rodríguez, Eduardo Navarro-Jimenez, José Francisco Tornero-Aguilera

**Affiliations:** 1 Faculty of Medicine, Health and Sports, Universidad Europea de Madrid, Villaviciosa de Odón, 28670 Madrid, Spain; vctxente@yahoo.es; 2 Study Center in Applied Combate (CESCA), 45007 Toledo, Spain; 3 Department of Health Sciences, Faculty of Biomedical and Health Sciences, Universidad Europea de Madrid, Villaviciosa de Odón, 28670 Madrid, Spain; lauraredondo_1@hotmail.com; 4 Psychology Department, Facultad de Ciencias de la Vida y la Naturaleza, Universidad Antonio de Nebrija, 28240 Madrid, Spain; abeltranv@nebrija.es; 5 Faculty of Education and Social Sciences, Universidad Andres Bello, Viña del Mar 2520000, Chile; rodrigo.yanez.s@unab.cl; 6 Faculty of Health Sciences, UNIE University, 28015 Madrid, Spain; 7 Facultad de Ciencias de la Salud, Universidad Simón Bolívar, Barranquilla 080002, Colombia; eduardo.navarro@unisimon.edu.co; 8 Graduate School of Business, Universidad ESAN, Alonso de Molina 1652, Santiago de Surco, Lima 15023, Peru; joseaguilera9@hotmail.com

**Keywords:** metabolomics, human dietary evolution, meat consumption, digestive adaptation, gut microbiota, ketogenic metabolism, enzyme evolution

## Abstract

This review examines human digestive physiology and metabolic adaptations in the context of evolutionary dietary patterns, particularly those emphasizing carnivorous and scavenging behaviors. By integrating metabolomic data with archaeological, anatomical, and microbiological evidence, the study explores how early hominins adapted to intermittent but energy-dense animal-based diets. The analysis highlights the development of hepatic insulin resistance, enhanced fat and protein metabolism, and shifts in gut microbiota diversity as physiological signatures of meat consumption. Comparative evaluations of digestive enzyme profiles, intestinal morphology, and salivary composition underscore humans’ omnivorous flexibility and partial carnivorous specialization. Additionally, biomarkers such as ketone bodies, branched-chain amino acids, and trimethylamine-N-oxide are identified as metabolic indicators of habitual meat intake. These adaptations, though once evolutionarily advantageous, are discussed in relation to current metabolic disorders in modern nutritional contexts. Overall, this review presents a metabolomic framework for understanding the evolutionary trajectory of human digestion and its implications for health and dietary recommendations.

## 1. Introduction

The study of human dietary evolution has long been a subject of interdisciplinary research, encompassing archaeology, anthropology, and molecular biology. While traditional approaches such as isotopic analysis and dental microwear studies have provided valuable insights into ancestral diets, the advent of metabolomics has revolutionized our understanding of human dietary adaptations. Metabolomics enables the identification of small-molecule metabolites associated with specific dietary patterns, offering a biochemical perspective on the human diet’s evolution [1]. Recent studies comparing the plasma metabolomes of individuals following different dietary regimes, including carnivorous and omnivorous diets, have revealed significant metabolic distinctions, underscoring the importance of dietary composition in shaping human metabolism [2].

One of the critical questions in human dietary evolution is the extent to which early hominins relied on animal-based foods. Archaeological evidence, including cut marks on fossilized bones and the presence of hunting tools, strongly suggests that meat consumption played a pivotal role in human evolution [3]. From a metabolomic perspective, dietary protein intake influences the endogenous metabolome by altering amino acid metabolism, nitrogen balance, and lipid utilization [4]. Comparative analyses of modern human metabolomic profiles indicate that individuals consuming high-protein diets exhibit unique metabolic markers, such as increased urea cycle activity and altered gut microbiota composition, similar to those found in carnivorous species [1,2,3,4,5].

Beyond direct protein metabolism, dietary fat utilization has also been a critical component of human dietary evolution. The ability to efficiently metabolize fats, particularly long-chain polyunsaturated fatty acids (PUFAs), is considered an adaptation that supported the rapid brain expansion observed in hominins [6], a fact that also allows for maintaining a high level of physical activity, allowing better adaptation to the environment [6,7,8]. Metabolomic studies have demonstrated that lipid metabolism in humans is highly responsive to dietary intake, with ketogenic pathways becoming more prominent in individuals consuming high-meat diets [9]. This metabolic flexibility is a distinguishing feature of humans compared to other primates, who rely more on carbohydrate fermentation in the gut [10].

Despite this evidence, the role of plant-based foods in human evolution remains significant, and metabolomic research has highlighted the complexities of dietary interactions. Phytochemicals, a major component of plant-based diets, influence gut microbiota composition and metabolic processes [1]. The presence of specific plant-derived metabolites in human plasma suggests that early hominins maintained an omnivorous diet, incorporating both animal and plant foods for optimal metabolic adaptation.

The incorporation of animal-based foods into the human diet has been a defining characteristic of hominin evolution, influencing not only metabolic processes but also anatomical and physiological adaptations. One of the key aspects distinguishing humans from other primates is the relative reduction in gut volume and increased reliance on more energy-dense foods, such as meat and fat, which require less extensive fermentation than plant-based diets [11]. This shift has been crucial in supporting the high-energy demands of an enlarged brain, a hallmark of human evolution. Unlike other primates, whose digestive systems are adapted for the slow fermentation of fibrous plant materials, humans have a relatively smaller colon and larger small intestine, reflecting a dietary adaptation that prioritizes rapid nutrient absorption from high-quality foods, including animal proteins and fats [12]. This anatomical shift is complemented by physiological adaptations, such as increased gastric acidity, which facilitates the digestion of animal proteins while acting as a defense mechanism against pathogens commonly found in scavenged or raw meat [13].

Metabolomic studies further reinforce the role of meat consumption in human evolution by demonstrating distinct lipid and protein metabolism pathways that favor the efficient utilization of dietary fats and amino acids [14]. Comparative research on modern humans adhering to different dietary patterns, such as vegetarian, omnivorous, and carnivorous diets, has revealed significant metabolic differences. For instance, individuals consuming meat-based diets exhibit higher levels of ketone bodies and branched-chain amino acids, indicating a metabolic reliance on fat oxidation and protein catabolism for energy production [6]. This metabolic flexibility, particularly in lipid oxidation and ketogenesis, would have provided early humans with a survival advantage in environments where carbohydrate-rich foods were seasonally scarce or unpredictable [15].

Additionally, gut microbiota composition provides further evidence of dietary adaptation. Comparative studies show that the human gut microbiome shares similarities with that of carnivorous species in its ability to process high-protein, low-fiber diets [10]. While herbivorous species rely on an extensive microbial community to break down complex polysaccharides, humans harbor microbial populations that efficiently metabolize proteins and fats, generating bioavailable energy sources such as short-chain fatty acids (SCFAs) and nitrogenous compounds [2]. These findings suggest that meat consumption was not merely an occasional dietary supplement for early hominins but rather a central component of their nutritional strategy. Moreover, stable isotope analysis of fossilized hominin remains consistently supports the hypothesis that early humans occupied a trophic position closer to that of carnivores than of omnivorous primates, further reinforcing the argument for a meat-rich diet [16].

In contrast to the microbial adaptations associated with meat-based diets, fiber-rich plant-based diets promote markedly different gut microbiota profiles. Complex carbohydrates and indigestible polysaccharides such as cellulose, hemicellulose, and resistant starches serve as primary substrates for microbial fermentation in the colon. This process supports the growth of saccharolytic taxa such as Prevotella, Ruminococcus, and Faecalibacterium, which are associated with the production of SCFAs like butyrate, propionate, and acetate [17]. These SCFAs are essential for colonic health and have been linked to anti-inflammatory effects and improved metabolic outcomes. The reduction in fiber intake associated with carnivorous or low-carbohydrate diets can, thus, lead to a shift toward proteolytic fermentation, characterized by an increase in taxa such as Bacteroides, Alistipes, and Bilophila. This dietary modulation underlies the observed differences in gut microbial composition and function among omnivorous, herbivorous, and carnivorous dietary patterns [18].

The metabolic adaptations to meat consumption in humans extend beyond simple macronutrient utilization and encompass a wide range of biochemical and physiological processes. Metabolomic analyses have identified specific biomarkers associated with habitual meat consumption, such as carnitine, creatine, and various nitrogenous compounds, which reflect the body’s capacity to efficiently metabolize animal proteins and fats [14]. These metabolites play critical roles in energy production, muscle function, and neural activity, suggesting that dietary meat intake has influenced key aspects of human physiology. Additionally, the presence of metabolites such as trimethylamine-N-oxide (TMAO) and branched-chain amino acids (BCAAs) in the plasma of individuals consuming high-meat diets further indicates distinct metabolic pathways that differentiate carnivorous from omnivorous or herbivorous species [19]. While excessive levels of TMAO have been linked to cardiovascular disease risks in modern populations, some researchers argue that its role in ancestral diets may have been functionally neutral or even beneficial in different ecological and physiological contexts [20].

Beyond individual metabolic markers, metabolomic studies have also revealed how meat consumption influences systemic metabolic regulation, particularly in relation to insulin sensitivity and energy homeostasis. Protein-rich diets have been shown to stimulate gluconeogenesis, the process of generating glucose from non-carbohydrate substrates, which is essential for maintaining stable blood sugar levels in low-carbohydrate conditions [21]. Moreover, ketogenic metabolism, characterized by the increased production and utilization of ketone bodies from dietary fats, has been identified as a crucial adaptation that may have allowed early humans to endure prolonged periods of fasting or seasonal food scarcity [22]. These findings align with anthropological evidence suggesting that early hominins were not only hunters but also scavengers, relying on intermittent access to animal-based foods to sustain high-energy demands [15]. The ability to efficiently extract and utilize energy from meat and fat, as evidenced by metabolomic research, underscores the evolutionary significance of carnivorous dietary patterns in shaping human metabolic flexibility.

Given the increasing evidence from metabolomics supporting the role of meat consumption in human dietary evolution, it is crucial to investigate how biochemical adaptations have shaped human metabolism in response to animal-based diets. Traditional archaeological and isotopic methods have provided substantial insights into early hominin diets, but metabolomics now offers a more detailed perspective on the physiological and molecular pathways that differentiate human metabolism from that of other primates [4]. Metabolic markers such as ketone bodies, BCAAs, and TMAO provide a biochemical signature of meat consumption and indicate metabolic flexibility in utilizing proteins and fats as primary energy sources [23]. The ability of humans to efficiently oxidize fatty acids and shift to ketogenesis under fasting conditions suggests an evolutionary advantage in environments where carbohydrate-rich foods were scarce or seasonally unavailable [24].

This study aimed to analyze human digestive physiology and metabolic adaptations to carnivorous and scavenger diets from a metabolomic perspective. By integrating data from paleontology, archaeology, digestive anatomy, enzymatic activity, and gut microbiota composition, this review will evaluate how humans evolved to process and metabolize animal-derived nutrients. Additionally, by comparing the metabolomic profiles of individuals consuming high-meat diets with those on plant-based diets, we seek to identify biomarkers that elucidate the extent to which human metabolism exhibits carnivorous adaptations. This research will contribute to evolutionary biology by providing a biochemical framework for understanding human dietary evolution, with potential implications for contemporary nutrition and metabolic health.

### Search Methods and Strategies for Research Identification

The protocol for this review followed a systematic literature search strategy, incorporating both primary sources (peer-reviewed scientific articles) and secondary sources (bibliographic indexes and databases). To ensure a comprehensive selection of relevant studies, searches were conducted using PubMed, Scopus, Web of Science, ScienceDirect, Embase, and SciELO. The literature search focused on identifying metabolomic, paleontological, physiological, and anthropological studies related to human digestive adaptations to carnivorous and scavenger diets. The keywords used in this search included paleodiet, human dietary evolution, meat consumption, scavenging behavior, hunting and scavenging in hominins, food processing in early humans, marrow consumption, isotopic analysis of diet, dental microwear, enamel isotopes, tooth morphology, gut microbiota composition, digestive adaptations, gastric acidity, intestinal length, protein digestion, lipid metabolism, ketogenic metabolism, BCAAs, TMAO, energy metabolism, insulin response, glucagon, leptin, ghrelin, metabolic homeostasis, microbial fermentation of protein and fat, gut microbiome of omnivores vs. carnivores, dietary lipid absorption, high-protein diet effects, genetic adaptation to meat consumption, metabolic flexibility in humans, dietary adaptation and brain evolution, and health implications of ancestral diets. The search included articles published from January 2010 to the present, prioritizing the most recent findings in metabolomics and human dietary evolution. However, foundational studies were included when they provided essential historical or theoretical background following previous procedures [25,26,27,28,29,30]. Studies were excluded if they (i) contained outdated data irrelevant to current metabolomic research, (ii) focused on unrelated topics, or (iii) were PhD dissertations, conference proceedings, abstracts, unpublished studies, or books.

## 2. Evolution of the Human Diet: Paleontological and Archaeological Evidence

The evolution of the human diet has been a central focus in paleoanthropology, revealing how our ancestors shifted from primarily plant-based foraging to incorporate significant animal-sourced foods. Fossil and archaeological evidence, such as isotopic signatures in bones and cut-marked animal remains, indicates that meat and fat became increasingly important in hominin nutrition over the last several million years [31]. Advancements in analytical techniques, including dental microwear textures, stable isotope and lipid residue analyses, now provide detailed insight into what our ancestors ate and how they obtained and processed those foods [32]. In this review, we refer primarily to hominins, a subgroup within the hominid family that includes modern humans (Homo sapiens) and their direct ancestors following divergence from the last common ancestor with chimpanzees. While the term *hominid* encompasses all great apes (including gorillas, orangutans, and chimpanzees), *hominin* is more specific to the evolutionary line leading directly to humans.

### 2.1. Meat Consumption in Ancestral Hominins

Early hominins diverged from the predominantly herbivorous diets of great apes and began incorporating animal foods well before the emergence of *Homo sapiens*. Stable isotope evidence of fossil teeth and bones provide a chemical record of diet. For example, carbon isotope analysis shows that many Australopithecines (4–2 Ma) had mixed diets, including significant C4 plant resources such as tropical grasses or sedges, unlike modern apes that eat almost exclusively C3 plants [33]. However, direct evidence of meat consumption in these early species is more elusive. A controversial but important finding from Dikika, Ethiopia, suggests that *Australopithecus afarensis* (~3.4 million years ago) may have occasionally eaten meat. Two animal bones from Dikika bear incisions that detailed analysis determined were not caused by trampling; the most plausible explanation is butchery with stone tools [34]. These 3.4 Ma bones have multiple V-shaped cut marks and percussion damage consistent with flesh slicing and marrow extraction, implying that *Australopithecus afarensis* used sharp stones to remove meat and crack bones [35]. If confirmed, this pushes back evidence of butchery by roughly 800,000 years earlier than previously thought, indicating that even pre-*Homo* hominins may have scavenged or hunted small game for meat on occasion [36].

By the dawn of the genus *Homo* (~2.8–2.5 Ma), meat eating appears to have become more regular. The earliest stone tools (Oldowan industry, ~2.6 Ma) coincide with increased evidence of carcass processing in the archaeological record [37]. Animal bones from several African sites around 2.5–2.0 Ma show cut marks and breakage patterns from stone implements, suggesting that early *Homo* was systematically butchering animals [38]. At Kanjera South in Kenya (~2.0 Ma), three well-preserved faunal assemblages provide clear evidence that Oldowan hominins acquired and processed numerous, relatively complete, small ungulate carcasses and even sometimes accessed larger animal remains [39]. These fossils show a repeated pattern of hominin butchery over thousands of years, representing the earliest known case of persistent carnivory in the hominin lineage [40]. The intensification of meat eating around 2.0 Ma likely reflects a significant adaptive shift early *Homo* becoming both hunters and efficient scavengers of meat, rather than only occasional consumers of animal foods [41].

Dental evidence further supports a dietary shift toward more animal food in early *Homo*. Morphologically, *Homo erectus* and related species evolved smaller teeth and gut size alongside larger brains, changes often linked to higher-quality diets rich in meat [42]. Microscopic wear patterns on teeth (dental microwear) in early hominins suggest a broad and variable diet. For instance, microwear and enamel chemistry in species like *Australopithecus africanus* and *Paranthropus boisei* contradict the idea that they were strict herbivores or seed crushers, suggesting a more generalized foraging pattern [43]. By the Pleistocene, members of the genus *Homo* were likely consuming meat regularly enough that it influenced dental and facial evolution, with smaller chewing muscles and teeth compared to *Australopithecus afarensis* [44]. The incorporation of calorie- and protein-rich animal foods is hypothesized to have fueled brain expansion in the genus *Homo* and may have allowed early humans to thrive in the savanna ecological niche [45].

Later Pleistocene hominins, including Neanderthals and early modern humans, left especially strong evidence of carnivorous diets. Isotopic analyses of fossil bone collagen have consistently shown Neanderthals to be top-level carnivores, with stable nitrogen isotopes (δ^15^N) indicating they obtained most of their dietary protein from large herbivorous prey [46]. The isotopic evidence indicates that in all cases, Neanderthals were top-level carnivores, whereas Upper Paleolithic modern humans (~40,000 BP) show more variable isotope values and some reliance on fish and other aquatic resources [47]. New analytical techniques reinforce this view: Zinc isotope ratios in tooth enamel from a 150,000-year-old Neanderthal in Spain place it high on the trophic ladder, indicating a heavily meat-based diet [48].

Importantly, different lines of evidence illustrate that Neanderthal diets were not entirely uniform. Dental calculus (tartar) microfossils from some Neanderthals in Iraq and Spain show bits of cooked starchy plants and even medicinal herbs, implying plant consumption when available [49]. Moreover, fecal biomarker analysis from soil at El Salt, Spain (~50,000 BP) detected coprostanol (a cholesterol byproduct) at high levels, consistent with a diet heavy in animal fat/protein, but also found 5β-stigmastanol, indicating significant ingestion of plant matter [50]. These integrated findings portray Neanderthals as primarily carnivorous hunters who supplemented their meat-heavy diet with plant foods as necessary a flexible omnivory skewed towards carnivory, likely shaped by Ice Age environments [51].

Overall, from the first stone-tool users to Late Pleistocene humans, the trend in our lineage was toward greater exploitation of meat. This dietary shift is recorded not only in cut-marked bones and isotopic ratios but even in our physiology (e.g., gut proportions, dentition) and is considered a cornerstone adaptation in human evolution.

### 2.2. Hunting Tools and Food Processing in the Fossil Record

The archaeological record reveals a co-evolution of diet and technology. As hominins became more carnivorous, they developed tools and techniques to acquire and process animal foods more efficiently. The earliest known stone tools date to about 3.3 million years ago from Lomekwi 3, Kenya, and predate the genus *Homo*, suggesting that tool use for food processing began with *Australopithecus afarensis* [52]. These very ancient tools are large, heavy-duty implements that may have been used to pound hard foods like nuts or tubers, or to break bones and cut flesh. By 2.6 Ma, a more refined stone technology, from the Oldowan industry, appears in East Africa, contemporaneous with evidence of systematic butchery [53].

Oldowan tools, including simple core flakes and choppers, were sharp enough to slice meat and tendons, scrape hides, and crack bones. Animal fossils from sites such as Gona and Olduvai Gorge (~2.5–1.8 Ma) bear telltale cut marks from stone flakes and percussion marks from hammerstones, indicating that early *Homo* used these tools to skin carcasses, fillet muscle, and extract marrow [54]. The pattern and location of cut marks typically found on bones at muscle attachments or around limb joints show that hominins were defleshing carcasses in much the same way modern butchers do, by cutting away meat and disarticulating limbs [55].

Over time, stone tool technology became more sophisticated, enhancing hominins’ hunting and processing capabilities. Around 1.7 million years ago, the Acheulean tool industry emerged, characterized by large bifacial handaxes. These teardrop-shaped handaxes and cleavers remained in use for over a million years, suggesting they were highly effective multi-purpose tools [56]. Although often thought of for woodworking or digging, experiments show Acheulean handaxes were efficient butchery tools their broad, sharp edges could efficiently joint large carcasses and slice meat. Acheulean sites such as Olorgesailie in Kenya or Gesher Benot Ya’aqov in Israel have yielded both stone handaxes and abundances of animal bones with cut marks, implying that *Homo erectus* and contemporaries were regularly butchering sizeable animals.

By the Middle Pleistocene, hominins also developed wooden and compound hunting weapons. The famous Schöningen spears from Germany, sharpened wooden spears dating to ~300,000 years ago, are the oldest complete hunting weapons found and demonstrate active big-game hunting by *Homo heidelbergensis* or Neanderthals [57]. These 2–2.5-m javelins were finely crafted and well balanced, suggesting they were used to stab or throw at large prey, such as horses, and represent a leap in hunting technology beyond stone tools alone. Likewise, stone-tipped spears (hafted stone points) appear in the archaeological record ~500,000 years ago, increasing the lethality of hominin weapons.

Hominins also innovated food processing techniques beyond cutting tools. A major technological milestone was the control of fire, which allowed cooking. Cooking directly affects diet by making foods especially meat and starchy plants easier to chew, detoxify, and digest, thereby increasing net energy gain. Though there are disputed claims of earlier fires, the oldest widely accepted evidence of controlled fire use is from Wonderwerk Cave in South Africa (~1.0 million years ago) [58]. There, archaeologists found unambiguous evidence in the form of burned bone and ashed plant remains, indicating that early *Homo erectus* was likely cooking its food.

By 400,000 years ago, fire use was habitual at sites like Qesem Cave (Israel) and Schöningen (Germany), where concentrated hearths, charred bones, and heat-altered stone tools have been discovered [59]. Cooking meat would have offered several advantages, evident in the archaeological and anatomical record: Softer, cooked meat and marrow can be eaten with smaller teeth and weaker jaws (matching the reduced dentition of *Homo erectus*); pathogens in meat are killed by heat (reducing disease); and cooked food yields more calories, supporting larger brains and bodies.

In addition to cooking, hominins developed other processing behaviors such as drying or caching food. Cut marks on 400,000-year-old deer bones from Qesem Cave show a unique pattern: They appear not to have been made when the animal was freshly killed, but after the outer flesh had been removed and the bones stored for a period [60]. Researchers concluded that Paleolithic humans at Qesem were storing ungulate leg bones with the skin on, then later stripping the dried skin and cracking the bone to eat the marrow, effectively preserving marrow as a food supply for up to nine weeks [61]. This surprising behavior indicates a planned, delayed consumption strategy akin to food storage, which was previously not thought to exist until much later in prehistory.

### 2.3. Marrow and Fat Consumption in Hominins

Nutrient-dense fat played a vital role in human evolution, and a wealth of evidence shows that our ancestors went to great lengths to extract fat from animal carcasses particularly in the form of bone marrow and brain tissue. Marrow, the fatty, calorie-rich tissue inside long bones, preserves well in the paleontological record through the damage patterns left on bones broken to access it. Paleoanthropologists have identified characteristic fracture and impact patterns in animal bones at hominin sites, revealing intentional marrow extraction.

Long bones from early Pleistocene sites often show spiral fractures (produced when fresh bones are broken) and percussion marks (dents or pits where hammerstones struck), indicating hominins smashed them soon after death to scoop out the marrow [61]. Notably, an analysis of bone assemblages from Olduvai Gorge (~1.8 Ma) led researchers to conclude that maximization of marrow (fat) yields, rather than muscle meat, was the criterion shaping decisions about carcass processing by early hominins [62].

Later hominins continued to avidly exploit marrow and fat with increasing efficiency. By the Middle Pleistocene, there is evidence of not only immediate consumption but also delayed consumption of marrow. The findings from Qesem Cave (~400–200 ka) are particularly revealing, showing that prehistoric humans stored ungulate long bones at the cave, still encased in dried skin as a natural *container*, then cracked them open weeks later when needed [63]. The presence of stored bones suggests that fat was a sufficiently critical resource to be preserved and rationed.

Biomolecular analyses have also begun to detect residues of animal fat on prehistoric tools, strengthening the case that hominins deliberately targeted fatty tissues. Residue analysis of stone tools from Qesem Cave identified preserved fat molecules, likely degraded bone grease, on the artifacts, providing direct evidence that those tools were employed in breaking bones to access marrow [64].

Thus, the convergence of paleoanthropological, archaeological, and biochemical evidence provides a robust understanding of how profoundly the human diet has changed since our ape ancestors. The fossil record, isotopic analyses, and tool evidence collectively show that early *Homo* species relied increasingly on meat and marrow, driving adaptations in brain size, dentition, and technological complexity. The mastery of hunting, butchery, fire use, and food preservation strategies underscored a major dietary transformation that played a central role in shaping human evolution. Ongoing interdisciplinary research will continue to refine our understanding of these dietary shifts and their implications for human adaptation.

## 3. Dental and Masticatory Adaptations

Diet has been a major driving force in human evolution, shaping the form and function of our teeth and jaws. The human masticatory apparatus, including teeth, mandible, maxilla, and chewing muscles, has adapted over millions of years in response to changing dietary behaviors. Unlike specialized carnivores or herbivores, humans are omnivores with a mixed diet, and our dentition reflects a balance of traits for processing both meat and plant foods [31].

### 3.1. Human vs. Carnivore and Herbivore Dentition

Human teeth differ markedly from those of strict carnivores and herbivores in terms of morphology, enamel characteristics, and jaw function. Carnivorous mammals, such as felids and canids, typically have pronounced canines for seizing prey and blade-like carnassial teeth for slicing flesh. Their jaw joints are configured for powerful vertical biting with minimal side-to-side motion, essentially functioning as a hinge optimized for crushing and tearing [65].

In contrast, herbivorous mammals, including ungulates such as cows and horses, lack strong canines and instead feature broad, ridged molars adapted for grinding tough plant matter. Herbivore jaws have a looser joint articulation, allowing significant horizontal (lateral) movements for chewing; this lateral translation capability, paired with well-developed dental ridges, enables the efficient shredding and grinding of fibrous vegetation [66]. Accordingly, their craniomandibular architecture differs: Carnivores have vertically oriented jaw muscles and joints optimized for forceful closure, whereas herbivores possess jaw joint surfaces and musculature that facilitate lateral motion and prolonged chewing [67].

Humans, as omnivores, occupy an intermediate position. Our jaws can move both vertically and slightly laterally, allowing some grinding. Our dentition includes incisors for biting, modest canines, and bunodont (low, rounded-cusp) molars suited for both slicing and crushing food [68].

Another key difference lies in enamel thickness and hardness. Enamel, the hard outer layer of teeth, varies with diet: Herbivores generally have thicker and harder enamel than carnivores, as a response to resisting wear from abrasive plant foods. Evidence show that herbivores tend to exhibit the highest enamel surface hardness, followed by omnivores, with carnivores having the lowest values [69]. Humans have notably thick enamel for a primate—considerably thicker than that of our great-ape relatives, such as chimpanzees and gorillas. Among primates, fruit- and leaf-eating species tend to have thin enamel, whereas omnivorous primates have intermediate enamel thickness, with *Homo sapiens* possessing some of the thickest enamel, an evolutionary hallmark linked to a broad diet and the need for durable teeth over a long lifespan [70].

Jaw biomechanics mirror these dental differences. Human jaw muscles (temporalis and masseter) are arranged to produce a balanced bite force and allow a degree of rotary chewing, unlike the highly specialized muscle arrangements in carnivores (built for biting force at the canines) or herbivores (built for endurance grinding). The human temporomandibular joint (TMJ) permits an oval motion during chewing, enabling both the vertical and slight lateral movements needed to process a varied diet [71,72]. Overall, the human dentition and jaws represent a compromise between the slicing apparatus of carnivores and the grinding mills of herbivores.

### 3.2. Molar Structure and Evolutionary Trends from Early Hominins to Humans

The structure of human molars—the large cheek teeth—has undergone notable changes through hominin evolution, reflecting shifts in diet and food processing techniques. Early hominins from the Pliocene (e.g., *Australopithecus*) had considerably larger molars and thicker enamel than both earlier apes and modern humans. The australopiths, living 4–2 million years ago, evolved megadont (large-toothed) dentitions with expansive, flat molar surfaces and thick enamel, especially in the robust australopiths (genus *Paranthropus*). These features suggest an adaptation for consuming tough, abrasive foods such as roots, tubers, and hard seeds [73].

For example, *Paranthropus boisei* (nicknamed “Nutcracker Man”) had enormous, flat molars with hyper-thick enamel, a deep robust mandible, and a skull with a prominent sagittal crest to anchor powerful chewing muscles—morphology indicative of extreme chewing forces [74]. In contrast, early *Homo* (starting ~2.5 Ma) showed a trend of dental reduction—smaller molars and jaws—which continued through *Homo erectus* and into *Homo sapiens* [75]. These trends likely reflect dietary shifts: increased consumption of animal protein, which is easier to chew than tough plant fiber, and the advent of food processing (such as cutting meat with tools and later cooking with fire), which softened foods before they entered the mouth.

The use of tools to slice meat and crack bones, and the controlled use of fire (at least by ~1 Ma and certainly by 0.5 Ma), reduced the need for extremely large teeth and jaws, favoring smaller faces and teeth in genus *Homo*. Experiments suggest that cooking tubers and meat greatly decreases the chewing effort and number of chewing cycles needed, which, over evolutionary time, would relax selection for massive teeth and jaws [76].

Within the genus *Homo*, molar structure became more refined. Early *Homo erectus* (~1.8 Ma) still had larger teeth than modern humans but they were smaller than those of australopiths, indicating a more generalized diet that included substantial meat [77]. By the time of anatomically modern humans (~300,000 years ago), molars had reduced further in size, and jawbones became thinner and less robust. This reduction co-evolved with cultural practices—not only cooking but also the grinding of grains, use of mortars, and other food preparation techniques in the later Pleistocene and Holocene, all of which decreased the toughness of foods entering the mouth [78].

Thus, dental and masticatory adaptations in human evolution reflect a complex interplay between biology and diet. Humans evolved from ape ancestors with relatively generalized omnivorous dentitions, and through time, our lineage experimented with both ends of the dietary spectrum from the tough plant processing of *Australopithecus* to the meat-heavy diets of later *Homo*. Compared to carnivores and herbivores, our dentition today is a mosaic: We lack the extreme slicing or grinding specializations, yet we have retained thick enamel, versatile jaw mechanics, and moderate tooth size, underscoring our omnivorous niche.

## 4. Digestive Anatomy and Physiology: Comparison with Carnivores and Omnivores

Carnivorous diets have left distinct metabolic footprints in human physiology. One of the most notable effects is observed in carbohydrate metabolism: Prolonged consumption of low-carbohydrate diets led to the development of hepatic insulin resistance. This adaptive mechanism favored endogenous glucose production through gluconeogenesis, ensuring a continuous supply of glucose for the brain in the absence of dietary carbohydrates [79]. Additionally, carnivorous feeding patterns have been associated with modifications in amino acid and purine metabolism. Animal studies have demonstrated shifts in the profile of circulating free amino acids, such as elevated levels of taurine, as well as alterations in purine degradation pathways—both of which are closely tied to high protein intake and reflect the biochemical demands of a meat-based diet [79]. These metabolic adjustments were paralleled by changes in the gut microbiome. Carnivorous species typically display reduced microbial diversity, including the loss of genes associated with mucosal immune defense, such as *NOX1*, and a diminished presence of microbial enzymes involved in the fermentation and degradation of plant-derived fibers, suggesting an ecological restructuring of the intestinal ecosystem in response to meat-centered nutrition [79].

The transition to a predominantly carnivorous diet during human evolution brought profound reproductive and metabolic consequences. One key advantage was the possibility of earlier weaning, which contributed to shorter interbirth intervals and, ultimately, increased reproductive rates, thereby reshaping human population dynamics over time [79]. However, several of the adaptive traits that emerged under conditions of nutritional scarcity have become maladaptive in modern dietary contexts. For instance, hepatic insulin resistance, which once conferred survival benefits by maintaining cerebral glucose availability in carbohydrate-deficient environments, now predisposes individuals to metabolic disorders such as type 2 diabetes when exposed to diets high in refined sugars and carbohydrates [80,81]. Furthermore, genomic evidence suggests that humans, like other carnivorous mammals, have experienced a progressive loss of detoxifying enzymes, including cytochrome P450 family members such as *CYP2C* and conjugating enzymes like *UGT*. These enzymes are responsible for neutralizing a broad spectrum of phytochemicals and environmental toxins. Their evolutionary reduction likely reflects ancestral dietary patterns with minimal exposure to plant-based xenobiotics, but in contemporary settings, this loss may increase vulnerability to synthetic and environmental toxins that were absent from prehistoric diets [82,83].

### 4.1. Digestive Anatomy and Physiology: A Comparative Overview of Carnivores and Omnivores

The digestive anatomy of carnivores and omnivores exhibits fundamental differences shaped by their distinct dietary requirements. A comparative analysis of their morphological and physiological traits highlights the evolutionary adaptations that support their respective feeding strategies.

In the oral cavity, carnivores such as cats (obligate carnivores) and dogs (facultative carnivores) possess sharp canines and well-aligned molars optimized for tearing flesh. Their saliva exhibits minimal α-amylase activity, reflecting a limited role in carbohydrate digestion [84]. In contrast, omnivores display a mixed dentition: sharp canines for tearing, complemented by flat molars designed for grinding plant matter. Their saliva often contains α-amylase, which initiates the enzymatic breakdown of carbohydrates within the mouth [85].

The stomach of carnivores is typically single-chambered and highly specialized for protein digestion. Felines, for example, rely predominantly on amino acids such as glutamate and aspartate as metabolic fuels for enterocytes [84]. While omnivores also possess a single-chambered stomach, it is less specialized. Nonetheless, it produces gastric acid and enzymes capable of digesting both animal and plant proteins, enabling a more flexible dietary intake [84,85].

The small intestine of carnivores is relatively short in proportion to body length, consistent with their capacity to rapidly and efficiently digest nutrient-dense animal proteins and fats. In felids such as lions and tigers, the small intestine measures approximately three to six times the body length [86]. In humans, by comparison, the adult small intestine spans about five meters [87]. Dogs, unlike cats, exhibit moderate pancreatic α-amylase activity, which enables partial starch digestion [84]. In omnivores, the small intestine is of intermediate length, supporting the digestion of a broad spectrum of foods. Certain omnivorous fish, for instance, show histological variations across intestinal segments as adaptations to their mixed diets [88].

The large intestine of carnivores is typically short and functions primarily in the fermentation of undigested proteins and water absorption. Microbial activity in this region is limited relative to that observed in omnivores [84,89]. In omnivorous species, the large intestine is generally more developed, allowing for microbial fermentation of plant materials. In animals such as bilbies, the cecum and colon serve as primary sites of microbial fermentation [85].

With respect to carbohydrate and plant metabolism, carnivores are adapted to diets low in carbohydrates and possess limited digestive capacity for plant-based components due to enzymatic and anatomical constraints [90]. In species such as lions and tigers, the relatively short intestinal tract enables the rapid transit of easily digestible meat, facilitated by proteolytic enzymes and highly acidic gastric secretions [86]. Omnivorous species, by contrast, demonstrate substantial dietary flexibility. For example, bilbies can efficiently digest both animal-based foods (e.g., mealworms) and plant-based matter (e.g., seeds), although their ability to process fiber-rich diets is constrained by reduced selective retention in the hindgut [85].

These anatomical and physiological differences reflect evolutionary adaptations to distinct dietary needs. Carnivores are biologically optimized for consuming protein-rich diets with minimal reliance on carbohydrate processing, whereas omnivores exhibit greater digestive versatility, enabling them to efficiently utilize a broad range of both animal and plant-based food sources (Table 1).

**Table 1:** Key comparative findings between carnivores and omnivores.

Characteristic	Carnivores	Omnivores
Dentition	Sharp canines for tearing flesh	Mixed dentition (canines for tearing, molars for grinding)
Stomach	Single-chambered; highly acidic and specialized for protein	Single-chambered; less specialized, capable of processing varied foods
Small Intestine	Short in proportion to body length	Intermediate length for broader nutrient absorption
Large Intestine	Simple and short; limited microbial fermentation	Longer; supports microbial fermentation of plant matter
Dietary Adaptability	Specialized for high-protein intake; limited carbohydrate processing	Flexible; capable of digesting both animal and plant-based foods

### 4.2. Human Intestinal Length in Comparison to Carnivorous and Herbivorous Species

The relative length of the human intestine, when compared to that of carnivorous and herbivorous species, reflects an omnivorous adaptation characterized by intermediate anatomical traits. Humans exhibit a gastrointestinal morphology that is neither as specialized for protein digestion as that of carnivores—who possess shorter intestines optimized for the rapid breakdown and absorption of animal proteins—nor as elongated as that of herbivores, whose extended intestines are designed to facilitate the microbial fermentation of fibrous plant material. Notably, the human small intestine is relatively long, enabling the efficient digestion and absorption of a broad range of macronutrients, including proteins, fats, and carbohydrates [91]. Leroy et al. have shown that the total intestinal length in humans, with a body length-to-intestine ratio of approximately 5:1, more closely resembles that of dogs (carnivores: 6:1) than that of ruminants such as cattle (herbivores: 12:1), further underscoring the human digestive system’s generalist configuration [92].

McKenney et al. reported substantial individual variation in human intestinal length. For instance, females consistently displayed longer small intestines than males, a difference that may reflect greater lipid absorption demands associated with reproductive functions such as gestation and lactation [91]. Furthermore, while small intestine length showed relative consistency, the colon exhibited greater variability, suggesting that reliance on microbial fermentation may differ across individuals. These findings support the *expensive tissue hypothesis*, which posits that dietary shift toward higher-quality foods—such as cooked starches and meat—reduced the evolutionary dependence on microbial fermentation in the colon, thereby allowing for an increased allocation of energy to brain development. This perspective aligns with the mosaic evolution hypothesis, which suggests that specific regions of the gastrointestinal tract evolved independently in response to dietary pressures. The human intestinal length exemplifies evolutionary adaptations to an omnivorous dietary strategy, balancing traits found in both carnivorous and herbivorous species. Importantly, considerable interindividual variability—driven by factors such as sex and diet—highlights the evolutionary trade-offs associated with dietary changes, including the adoption of cooking and the reduced reliance on microbial digestion [13,92].

### 4.3. Presence and Functionality of the Human Cecum and Its Dietary Implications

Although considerably smaller than in herbivorous species, the human cecum retains important functions related to microbial fermentation, SCFA production, and intestinal homeostasis. Its functional relevance is closely linked to dietary fiber intake, with significant implications for inflammation regulation and metabolic health [93,94,95]. Bacterial fermentation of dietary fiber in the cecum results in the generation of SCFAs, including butyrate, propionate, and acetate. Among these, butyrate serves as the primary energy source for colonocytes, enhances mucosal immunity, and plays a key role in suppressing intestinal inflammation [95,96]. Experimental studies in mice have demonstrated that cecal removal reduces SCFA levels by approximately 50%, leading to a decline in microbial diversity and an increased susceptibility to infections [95].

The colon hosts a diverse anaerobic microbiome, which is essential for colonization resistance against pathogenic organisms. Disruption or loss of the cecal microbiota is strongly associated with dysbiosis and a subsequent decrease in anti-inflammatory SCFA levels [97]. In addition, SCFA-mediated pH reduction in the colon facilitates the absorption of key minerals such as calcium and magnesium [97,98]. Diets deficient in fiber reduce SCFA production, a phenomenon linked to the pathogenesis of inflammatory bowel disease, type 2 diabetes [99], and atherosclerosis [100] and an increased risk of colorectal cancer [101,102].

From an evolutionary perspective, the relatively small size of the human cecum is consistent with the *expensive tissue hypothesis*, which proposes that the reduction in gut size was an adaptive trade-off that favored increased brain development at the expense of microbial digestive capacity [103,104]. Then, while the human cecum is reduced in size, it retains critical fermentative functions that are dependent on dietary fiber intake. Its anatomical reduction reflects evolutionary adaptations to high-quality diets but also underscores the modern necessity for fiber-rich nutrition to maintain intestinal health through SCFA-mediated mechanisms.

### 4.4. Gastric Emptying Rate and Stomach Acidity in Relation to Meat Digestion

Gastric emptying rate and gastric acidity are tightly integrated with the digestion of meat, involving a coordinated interplay of structural, enzymatic, and regulatory mechanisms. These factors operate in concert to ensure the effective breakdown and assimilation of animal protein.

The structural complexity of meat, characterized by its dense protein matrix and fibrous texture, resists rapid degradation. This necessitates prolonged mechanical breakdown via antral contractions, which delays gastric emptying compared to homogenized or liquid meals. Studies have demonstrated that fat- and protein-rich boluses—similar in composition to meat—tend to sediment within the stomach, sequestering nutrients and further slowing their release into the duodenum [105].

Particle size regulation is another key determinant of gastric transit. The pyloric sphincter restricts the passage of gastric contents to particles smaller than 2 mm. Larger fragments of meat are retained in the stomach for further mechanical breakdown, thereby prolonging gastric residence time. While this facilitates efficient protein hydrolysis, it also delays nutrient delivery to the small intestine [106,107].

Protein denaturation is catalyzed by the highly acidic gastric environment (pH 1–3), which unfolds the tertiary structure of meat proteins, exposing peptide bonds to enzymatic cleavage by pepsin. Insufficient gastric acidity impairs this process, resulting in poorly digested meat residues [107,108]. The activation of pepsin is pH-dependent, with optimal conversion of pepsinogen to pepsin occurring at pH < 4, and maximal enzymatic activity at pH 2. In conditions of hypochlorhydria (pH > 5), pepsin remains inactive, reducing proteolysis and, thereby, prolonging gastric retention of undigested protein particles [109,110].

Acidic chyme produced during meat digestion stimulates pH-sensitive receptors in the duodenum, triggering the release of regulatory hormones such as secretin. This feedback mechanism inhibits gastric emptying, preventing intestinal overload and ensuring adequate neutralization and enzymatic processing in the small intestine [106,111].

Nutrient-dependent signaling also plays a central role. Hydrolyzed proteins and amino acids derived from meat digestion stimulate gastrin secretion, which enhances both gastric acid production and antral motility. This creates a positive feedback loop in which protein-rich meals promote their own digestion by increasing the functional capacity of the stomach [112,113].

High-protein diets further enhance gastric acid secretion due to both the buffering capacity of meat and the presence of specific amino acids (e.g., phenylalanine, tryptophan). This acid response compensates for the structural resistance of intact meat, balancing mechanical breakdown and chemical digestion to optimize gastric emptying [114].

The rate of gastric emptying also varies with the physical form of the meat. Mechanically processed meats (e.g., ground beef) empty more rapidly due to reduced structural integrity, whereas whole cuts require prolonged acid and enzymatic action. For example, liquid protein shakes bypass structural delay and are emptied from the stomach faster than their solid counterparts [105,115].

Clinically, the use of proton pump inhibitors (PPIs), which elevate gastric pH above 4, compromises pepsin activity and impairs protein digestion. This not only delays gastric emptying but may also increase intestinal permeability and the risk of malabsorption [116,117].

## 5. Digestive Enzyme Production and Dietary Implications

Digestion in animals relies on a suite of enzymes tailored to break down proteins, carbohydrates, and fats. The profile and activity of these digestive enzymes have evolved in accordance with dietary habits [118]. Species that consume starch-rich diets tend to express higher levels of amylases, whereas strict carnivores primarily invest in proteases to maximize protein digestion [119].

Humans, as omnivores, produce a broad spectrum of digestive enzymes, yet there are notable constraints, particularly regarding the breakdown of certain complex plant polysaccharides. Unlike herbivorous species, which possess extensive microbial fermentation capabilities in specialized gut chambers, the human digestive system has limited endogenous enzymatic capacity to process fiber [120].

The evolution of digestive enzymes in humans reflects a key metabolic adaptation to dietary transitions throughout hominin evolution. Proteolytic enzymes such as pepsin, trypsin, and chymotrypsin became essential for efficiently extracting amino acids from animal-derived proteins. This enzymatic capacity not only enabled early humans to rely on intermittent but nutrient-dense meat consumption but also contributed to metabolic flexibility by supporting gluconeogenesis and ketogenesis in carbohydrate-scarce environments [121]. In parallel, lipase activity—particularly, pancreatic lipase and lingual lipase—facilitated the rapid hydrolysis and absorption of fats, supporting sustained energy production during periods of fasting or food scarcity [122]. Compared to herbivorous species, humans exhibit a reduced expression of cellulolytic enzymes, reflecting an evolutionary shift away from fiber-rich diets [123]. These enzyme profiles mirror the broader metabolic transition toward reliance on animal proteins and fats, as documented in metabolomic biomarkers such as branched-chain amino acids, carnitine, and ketone bodies.

### 5.1. Pepsin and Proteases in Protein Digestion

Protein digestion begins in the stomach with pepsin, an aspartic protease that is activated in the highly acidic gastric environment. Pepsin is the primary protease in human gastric juice, initiating the breakdown of complex proteins into peptides. It is secreted as an inactive precursor, pepsinogen, by chief cells in the gastric mucosa and is activated when the stomach pH falls below ~2–4, a process that involves auto-catalytic cleavage [124]. In humans, pepsin, in conjunction with hydrochloric acid (HCl), plays a fundamental role in protein digestion by denaturing muscle fibers and exposing peptide bonds for enzymatic cleavage [125]. The low gastric pH not only optimizes pepsin activity but also serves as a protective mechanism against ingested pathogens. Pepsin typically hydrolyzes approximately 10–20% of dietary protein within the stomach, producing peptide fragments that further stimulate digestive processes in the small intestine [126].

Once chyme enters the duodenum, pancreatic proteases take over. The pancreas secretes inactive zymogens, including trypsinogen, chymotrypsinogen, and procarboxypeptidases, which are subsequently activated in the small intestine to trypsin, chymotrypsin, and carboxypeptidase. These enzymes systematically cleave peptide bonds, reducing polypeptides into oligopeptides and free amino acids, which are further processed by brush-border peptidases for absorption. This two-phase digestive strategy allows humans to efficiently assimilate protein, with evidence indicating that lean meat protein exhibits > 90% digestibility when properly cooked and chewed [127].

Comparative studies indicate that pepsin and analogous gastric proteases are ubiquitous among carnivorous and omnivorous vertebrates, though their activity varies depending on stomach acidity and dietary protein reliance. Obligate carnivores, such as felids, typically exhibit highly acidic gastric environments (pH ~1–2), which ensures full activation of pepsin and enables rapid protein breakdown. In contrast, herbivorous mammals, particularly foregut fermenters, maintain higher gastric pH values (pH 4–7), as they depend more on microbial fermentation rather than enzymatic hydrolysis for protein acquisition. Interestingly, humans exhibit gastric pH levels around 1.5, akin to scavengers, suggesting an evolutionary advantage in omnivorous diets, possibly to aid in pathogen elimination and the efficient digestion of animal proteins [128].

Protein-digesting enzymes in other species share functional similarities with human pepsin and pancreatic proteases, though they may differ in isoforms and enzymatic output. Ruminant herbivores, for example, secrete lysozyme in the abomasum to hydrolyze bacterial cell walls, as their primary protein source comes from microbial fermentation. Additionally, carnivorous and insectivorous species often produce chitinases in the stomach or pancreas, allowing them to digest chitin-rich prey, a capability largely absent in humans [129]. Overall, while the core suite of gastric and pancreatic proteases in humans is comparable to that of other omnivores, obligate carnivores exhibit a more specialized and intensified proteolytic capacity, reflecting their reliance on animal protein. Evolutionarily, species that have transitioned to higher-protein diets often show upregulation or gene duplications of proteases, and some species even induce new proteases during developmental shifts toward carnivory, such as in amphibians undergoing metamorphosis.

These comparative insights emphasize that pepsin and proteases are evolutionarily conserved but their levels, specificity, and gastric environment are tuned to an organism’s dietary composition. Humans, as omnivores, maintain a balance between proteolytic efficiency and digestive flexibility, facilitating the consumption of both plant and animal-derived protein sources.

### 5.2. Limited Capacity for Complex Polysaccharide Digestion in Humans

Humans possess a well-developed capacity for digesting simple sugars and starch but have a limited ability to break down complex polysaccharides such as dietary fiber. The digestion of starch begins in the oral cavity with the action of salivary α-amylase, which initiates the hydrolysis of α-1,4 glycosidic bonds in starch. This process continues in the small intestine, where pancreatic amylase further degrades starch into maltose and smaller oligosaccharides, which are subsequently broken down into absorbable monosaccharides by brush-border enzymes such as maltase, isomaltose, and sucrase. However, not all starch is fully digestible [130]. A portion, called resistant starch, resists amylase hydrolysis due to its physical structure or retrogradation properties and remains intact until reaching the colon, where it undergoes microbial fermentation.

While humans efficiently digest starch, they lack the necessary enzymes to break down plant structural polysaccharides, particularly cellulose. Cellulose, the primary constituent of plant cell walls, remains indigestible because humans do not produce cellulase, the enzyme required to hydrolyze β-1,4 glycosidic linkages. Similarly, hemicelluloses, pectins, and other non-starch polysaccharides are resistant to human digestive enzymes and largely bypass enzymatic degradation in the small intestine. As a result, these fibers reach the large intestine, where microbial fermentation occurs [131].

Unlike ruminants and hindgut-fermenting herbivores, which possess specialized gut chambers and symbiotic microbiota for fiber degradation, humans have only a modest fermentative capacity. The human proximal colon, while functionally analogous to the enlarged cecum of herbivores, is far less efficient at extracting energy from fiber. However, a diverse gut microbiota enables partial fiber breakdown, converting undigested carbohydrates into SCFAs such as acetate, propionate, and butyrate, which serve as energy substrates for colonic cells and contribute to metabolic health. The presence of fiber-degrading bacterial species in the human colon, some of which are also found in herbivores (e.g., *Ruminococcus* and *Bacteroides*), suggests that humans retain a limited ability to extract energy from plant cell walls through microbial fermentation [128].

Despite microbial assistance, humans derive minimal caloric value from dietary fiber. Most fiber is excreted after fermentation, contributing only a small fraction of daily energy intake. The relatively low fiber consumption in modern diets further limits microbial energy extraction. Even under high-fiber dietary conditions, the absence of a large fermentation chamber (such as rumen) imposes physiological constraints on fiber utilization, reinforcing the necessity for readily digestible carbohydrates or animal-derived nutrients in human diets [132].

Cooking and food processing can increase nutrient availability in fibrous foods by softening plant cell walls, thereby enhancing enzymatic access. However, the human digestive system remains primarily adapted to digestible starches and sugars, with gut microbiota compensating for fiber digestion to a limited extent. In contrast, herbivores exhibit intricate symbioses and morphological adaptations—such as multi-chambered stomachs or enlarged ceca—that maximize fiber degradation and microbial fermentation. This fundamental difference underscores why humans must rely on more energy-dense foods, such as processed plant carbohydrates or animal products, to meet metabolic demands [133].

### 5.3. Enzymatic Profiles of Carnivores vs. Omnivores (Humans)

Species-specific differences in digestive enzyme production reflect long-term dietary specializations. The digestive systems of strict carnivores (obligate meat-eaters) and omnivores (such as humans) have evolved to optimize the breakdown of macronutrients through key enzymatic adaptations [134,135,136].

#### 5.3.1. Carbohydrate-Digesting Enzymes (Amylases)

Omnivores generally produce more amylases than carnivores, consistent with a higher intake of dietary starch. Humans, for instance, exhibit high levels of salivary and pancreatic amylases, encoded by the *AMY1* and *AMY2* genes, respectively. Populations with historically starch-rich diets evolved additional copies of the *AMY1* gene, leading to elevated salivary amylase activity. Humans can carry between 2 and 15 copies of *AMY1*, significantly more than non-human primates, which typically have only two copies. This genetic adaptation reflects the evolutionary shift toward starch-based diets, incorporating tubers, grains, and cooked roots [137].

By contrast, strict carnivores exhibit minimal amylase activity. For example, cats produce no salivary amylases, and their pancreatic amylase secretion is relatively low compared to omnivores. Wild carnivores such as wolves also demonstrate limited starch digestion capacity, possessing only two copies of the *AMY2B* gene and lacking salivary amylase entirely. Interestingly, domesticated dogs, due to their co-evolution with humans and adaptation to starch-rich diets, have developed a higher pancreatic amylase output, with some individuals carrying between 4 and 30 copies of *AMY2B*. This enzymatic plasticity highlights the evolutionary flexibility of omnivores compared to the rigid metabolic specialization of obligatory carnivores [138].

Other omnivorous species, such as pigs and rats, also exhibit substantial amylase activity—some in both saliva and pancreas—to accommodate plant matter in their diet. In summary, the presence, quantity, and regulation of amylase serve as a distinguishing feature between dietary specializations: Omnivores like humans rely on amylase to break down starch before absorption, while strict carnivores largely depend on gluconeogenesis from protein and produce minimal amylase [139].

#### 5.3.2. Protein-Digesting Enzymes (Proteases)

Both carnivores and omnivores require robust protease activity, but carnivores typically demonstrate higher baseline protease levels to cope with protein-dense meals. All mammals produce a similar suite of pancreatic proteases (e.g., trypsin, chymotrypsin, elastase, carboxypeptidases) and gastric pepsin, but their secretion rates and regulation differ [140]. Obligate carnivores, such as felines, have higher dietary protein requirements and do not downregulate protein catabolic pathways, even when consuming lower-protein diets—an evolutionary adaptation to consistent meat consumption. Their stomach acidity is also greater, with a pH as low as 1.0–2.0, optimizing protease activity and facilitating rapid protein breakdown. This combination of high protease secretion and gastric acidity allows for the efficient digestion of large, infrequent meals typical of carnivores [141].

Humans, as omnivores, produce substantial proteases, but their total protein intake is lower than that of felines or wild carnivores. While a human pancreas and a canine pancreas generate the same proteolytic enzymes, carnivores often have higher enzyme output per kilogram of body weight. Some carnivores also secrete gastric chitinase, an enzyme specialized in digesting insects and crustacean exoskeletons, which are either absent or vestigial in humans. Conversely, omnivores produce enzymes such as sucrase-isomaltose, enabling the digestion of plant-derived sugars, which carnivores metabolize inefficiently [142]. Another noteworthy adaptation in carnivores that consume whole prey is the presence of gastric lysozyme, an enzyme that facilitates the digestion of bacterial cell walls, providing an additional protein source from gut microbiota. This specialization is absent in strict herbivores but occurs in some ruminant-feeding carnivores (e.g., hyenas), which ingest partially digested plant matter from their prey’s stomachs [143].

#### 5.3.3. Fat-Digesting Enzymes (Lipases)

The ability to digest fats is critical for both carnivores and omnivores, as lipids represent a major energy source. The primary enzyme involved is pancreatic lipase, which hydrolyzes triglycerides into fatty acids and monoglycerides, facilitating absorption in the small intestine. While all mammals produce pancreatic lipase (PL), auxiliary lipases differ between dietary groups. Carnivores such as cats and dogs secrete high levels of pancreatic lipase to efficiently digest animal-derived fats [144]. However, they lack pancreatic lipase-related protein 2 (PLRP2), an enzyme found in many omnivores and herbivores that assists in digesting plant-derived fats, particularly glycolipids. Research indicates that cats and dogs do not express PLRP2, whereas omnivores (e.g., humans, pigs, rats) and non-ruminant herbivores (e.g., horses, rabbits) produce both classical lipase and PLRP2, reflecting their broader dietary spectrum [144].

The absence of PLRP2 in carnivores can be explained by dietary consistency: Animal-based foods contain triglycerides and phospholipids but negligible glycolipids, making PLRP2 functionally unnecessary. Humans, consuming both animal fats and plant oils, have retained PLRP2, conferring a more versatile lipid digestive capacity. Moreover, carnivores tend to have shorter intestines, requiring efficient and rapid fat digestion and absorption. Their bile secretion is also optimized for handling high dietary fat loads, whereas omnivores must accommodate both moderate and low-fat diets. Interestingly, human saliva contains a lingual lipase, an enzyme that initiates fat digestion in the stomach, a feature shared with some wild carnivores (e.g., neonatal mammals relying on milk digestion) [145].

#### 5.3.4. Evolutionary Considerations

The enzymatic adaptations observed in carnivores and omnivores reflect evolutionary trade-offs shaped by diet. Gene copy variation plays a crucial role: Humans and dogs have evolved multiple amylase gene copies in response to starch-rich diets, while cats have lost the need for amylase entirely. Similarly, the absence of PLRP2 in carnivores and its retention in omnivores illustrates how dietary pressures drive enzyme evolution. Regulatory plasticity is also evident: Omnivores can adjust digestive enzyme production based on diet (e.g., increasing amylase secretion on high-carbohydrate diets), whereas carnivores have a more fixed enzymatic profile, reflecting their specialized reliance on protein and fat metabolism. These physiological distinctions highlight how dietary composition directly influences enzyme expression and digestive efficiency, demonstrating a clear evolutionary relationship between feeding strategy and biochemical adaptation [146].

Thus, digestive enzyme production in humans reflects our omnivorous heritage, equipping us to process a diverse range of foods while lacking the extreme specializations seen in strict carnivores or herbivores. Pepsin and other proteases enable efficient protein digestion, functioning similarly to those found in carnivores but without the extreme adaptations observed in obligate meat eaters. Carnivores possess higher protease secretion rates and consistently low gastric pH, whereas human proteolytic activity remains adaptive and intermediate—effective for meat digestion but not as highly optimized as in true carnivores [120]. In contrast, human carbohydrate digestion is significantly more developed than that of carnivores. High salivary and pancreatic amylase levels allow efficient starch hydrolysis, a trait shared with other omnivores and reflecting an evolutionary reliance on starch-rich foods. Comparatively, strict carnivores have minimal carbohydrase activity, lacking salivary amylases entirely, while possessing only low pancreatic amylase output [146].

Despite our proficiency in digesting starches and proteins, humans lack endogenous enzymes for breaking down most dietary fibers, underscoring a significant digestive limitation. Instead, we depend on gut microbiota to ferment indigestible plant polysaccharides—an approach resembling herbivorous digestive strategies, albeit in a rudimentary form. While herbivores possess large fermentation chambers (i.e., rumen, cecum), humans rely primarily on colonic fermentation, yielding only a modest energy return in the form of SCFAs. These enzymatic distinctions have clear dietary implications. Humans thrive on a mixed diet, obtaining energy efficiently from starches, proteins, and fats, but deriving little direct energy from raw plant fiber. Conversely, carnivores struggle to metabolize starch and cannot subsist on plant matter alone due to their high reliance on proteolytic and lipolytic pathways. Evolution has finely tuned digestive biochemistry in each lineage through gene duplications, functional losses, and regulatory adaptations, aligning enzyme profiles with dietary availability [147].

Understanding these enzyme differences underscores why diet must match an animal’s biological design. For humans, high-quality proteins and accessible carbohydrates (alongside fiber for gut health) best complement our enzymatic capabilities. Extreme dietary patterns—such as all-raw greens or exclusively raw meat—may challenge our intermediate physiology, as our digestive system is not optimized for highly specialized feeding strategies. Ongoing research in genomics and microbiome science continues to illuminate the co-evolution of human digestive enzymes and gut microbes, offering deeper insights into nutritional health and the evolutionary forces that shaped human digestion.

## 6. Macronutrient Metabolism in Humans

Metabolic diseases are associated with the nutrition transition, which is defined by a change from traditional to modern diets that are low in nutritional diversity and high in energy density. The human diet has different components, including nutrients that supply critical materials for numerous metabolic activities in every cell, along with non-nutrients [148]. These components and their metabolites can control gene expression and cellular function through several methods. Certain components are advantageous, whereas others exhibit harmful consequences [148]. In this regard, human metabolism has developed to effectively utilize proteins and fats as principal energy sources [149], especially when the carbohydrate supply is restricted. This adaptation is essential for survival in conditions with limited or irregular dietary glucose intake, setting humans apart from strictly herbivorous creatures that primarily depend on carbohydrates for energy [150]. Although carbohydrate-rich foods are essential for a nutritious diet, as they supply the necessary glucose for biological functioning and physical activity, the excessive intake of processed, simple, and low-quality carbohydrates directly affects physical and mental pathology. In fact, carbohydrate consumption is hypothesized to be a significant influence in the emergence of the predominant Western diseases of the 21st century [151]. Given these metabolic dynamics, it is crucial to examine how the body processes the conversion of macronutrients into energy and the perspective specifically from a high-protein diet.

### 6.1. Efficient Conversion of Proteins and Fats into Energy: A Lower Dependence on Carbohydrates

Unlike herbivorous species, whose digestive systems are specialized for fermenting plant matter to extract energy from carbohydrates, humans have developed versatile metabolic pathways that allow for the efficient utilization of fats and proteins as alternative energy sources [128]. As explained many years ago by Alberts and collaborators in 2002, cells break down food molecules to generate chemical energy in the form of Adenosine triphosphate (ATP) and Nicotinamide adenine dinucleotide (NADH). This procedure occurs in three primary phases. Initially, during digestion, macromolecules from food, including proteins, lipids, and polysaccharides, are decomposed into their monomers, such as amino acids, fatty acids, and sugars, either extracellularly or within specialized organelles like lysosomes. Secondly, in glycolysis, glucose is transformed into pyruvate in the cytosol via a sequence of events that produce ATP and NADH. Pyruvate is subsequently carried to the mitochondria, where it is transformed into acetyl-CoA [152]. Third, in the citric acid cycle and oxidative phosphorylation, acetyl-CoA enters the citric acid cycle within the mitochondria, generating NADH and FADH_2_, which convey electrons to the electron transport chain situated in the inner mitochondrial membrane, where the released energy facilitates ATP synthesis and oxygen consumption. This multi-step process allows cells to effectively derive energy from food molecules to maintain essential processes [152].

Protein synthesis, an essential activity in all biological systems, is crucial for maintaining life and promoting organism growth and development [153]. Corsetti et al. describe a recent study that offers a distinctive viewpoint highlighting the complex interactions among energy, dietary proteins, and amino acid composition, emphasizing their interdependence for health-related implications [154]. Protein synthesis is an energy-intensive process, requiring at least four ATP molecules for each peptide bond formed. The creation of a typical protein, such as albumin, requires more than 2900 ATP molecules. ATP molecules are produced by the mitochondrial oxidation of around 60–70 glucose molecules, provided that cellular metabolism functions with low entropy [155]. This process is governed by complex metabolic systems. Indeed, as previously said, both energy generation and protein synthesis must be meticulously regulated to fulfill cellular and biological requirements. Concretely, AMP-activated protein kinase (AMPK) is a crucial energy sensor that regulates metabolic processes in response to cellular energy levels [156].

When ATP levels drop and AMP levels rise, AMPK becomes activated, inhibiting anabolic pathways like protein, fatty acid, and cholesterol synthesis while promoting catabolic processes such as glucose uptake, fatty acid oxidation, and autophagy (AUT) [157]. AMPK suppresses mTOR activity, thereby regulating protein synthesis and facilitating cell adaptation to energy stress. AUT, a self-degradative process, helps maintain energy homeostasis by recycling cellular components, particularly during metabolic stress. Additionally, essential amino acids (EAAs), particularly branched-chain amino acids like leucine, play a pivotal role in activating mTOR, modulating protein synthesis, and influencing mitochondrial biogenesis [158,159]. The mTOR pathway integrates nutrient and energy signals to balance anabolic and catabolic processes, promoting cell growth and metabolism when energy is sufficient and triggering autophagy under low-energy conditions. Moreover, transcription factors like ATF4 respond to nutrient deprivation and mitochondrial stress, regulating gene expression to support protein folding, antioxidant defense, and autophagy-related processes [154].

Fat metabolism provides a highly efficient and sustained energy supply, particularly during periods of fasting, prolonged exercise, or carbohydrate restriction [160]. The process begins with lipolysis, where stored triglycerides in fat cells break down into glycerol and free fatty acids [161,162]. Panov and collaborators pointed out again, recently, that glycerol can be converted into glucose through gluconeogenesis, while free fatty acids are transported into the bloodstream to be used by cells for energy production. Inside the cells, fatty acids undergo β-oxidation in the mitochondria, where they are broken down into two-carbon units called acetyl-CoA while also generating high-energy electron carriers NADH and FADH_2_. These molecules then enter the citric acid cycle, also known as the Krebs cycle, where acetyl-CoA undergoes further reactions producing more NADH and FADH_2_ as well as releasing carbon dioxide as a byproduct [163]. The final step occurs in the electron transport chain, where NADH and FADH_2_ donate electrons to a series of protein complexes in the mitochondrial membrane, creating an electrochemical gradient that drives ATP synthesis through oxidative phosphorylation, ensuring maximum energy yield [163].

Thus, when carbohydrate intake is low, an alternative pathway called ketogenesis takes place in the liver, where excess acetyl-CoA is converted into ketone bodies such as acetoacetate β-hydroxybutyrate and acetone. These ketones serve as an alternative energy source particularly for the brain, heart, and muscles since fatty acids themselves cannot directly cross the blood–brain barrier. Ketone bodies are water-soluble, making them easily transportable in the bloodstream, providing a continuous energy supply in the absence of glucose [164]. This metabolic flexibility is essential in contexts where carbohydrate intake is limited, such as during fasting, ketogenic diets, or prolonged caloric restriction. Then, fatty acids can be used directly as an energy source by most tissues, except for the brain and red blood cells, which rely primarily on glucose for their function [165].

Knowing this process mentioned above, it could be said that humans exhibit a lower dependence on carbohydrates than herbivorous species, which are adapted to extract and utilize energy primarily from fiber fermentation [166,167,168]. In contrast, the human digestive system lacks specialized fermentation chambers, such as the rumen found in ruminants, and has a relatively small cecum, limiting the capacity for fiber digestion [169,170]. This anatomical and physiological design supports a diet that includes higher proportions of fats and proteins, as evidenced by the relatively higher activity of lipases and proteases compared to cellulases in the human digestive tract.

### 6.2. Impact of Low-Carbohydrate Diets on Human Metabolic Regulation

Low-carbohydrate diets have been shown to induce significant metabolic adaptations, including increased fatty acid oxidation, enhanced insulin sensitivity, and reduced glucose fluctuations. In fact, recent guidelines such as those of Foley and colleagues have acknowledged and promoted the utilization of a low carbohydrate diet (LCD) as a legitimate alternative for patients with T2DM and obesity [171]. Systematic, evidence-informed education must be accessible to all physicians to enhance confidence and guarantee consistency and quality assurance. It is based on the idea that the transition to a fat-based metabolism is marked by a reduction in insulin secretion, promoting lipolysis and reducing dependency on glycogen stores [172].

Although in clinical studies, low-carbohydrate and ketogenic diets have demonstrated benefits for metabolic health, including improved glycemic control in individuals with type 2 diabetes, reduced markers of inflammation, and enhanced mitochondrial efficiency [173,174], long-term adherence to such diets remains an object of debate due to potential concerns regarding nutrient balance, gut microbiota alterations, and cardiovascular implications [175]. However, a recent study investigated the effects of a high-carbohydrate/low-fat (HC) diet compared to an LCD on physically active adults. Over a three-week period, participants followed an HC diet, then underwent a wash-out phase, and subsequently adhered to an LCD. The study assessed physical performance, body composition, and blood parameters after each dietary phase. The findings revealed that the LC diet led to significant reductions in body weight and fat mass without compromising physical performance. In contrast, the HC diet did not result in notable changes in these parameters. These results suggest that an LCD may be more effective for body composition improvements while maintaining performance in physically active individuals [176]. Also, Santamarina et al. exposed that a low-carbohydrate diet facilitates weight reduction and enhances glucose intolerance in individuals with obesity. The quality of lipids directly affects outcomes, indicating that the intake of ω-3 polyunsaturated and ω-9 monounsaturated lipids can enhance glucose intolerance, lipid metabolism, and anti-inflammatory effects [177].

In conclusion, human metabolism is highly adaptable, allowing for efficient energy extraction from proteins and fats while maintaining metabolic flexibility in response to dietary composition. This ability has played a crucial role in human evolution and continues to have implications for modern dietary strategies and metabolic health.

## 7. Metabolomic Profile of Individuals on Carnivorous Diets

Carnivorous diets, which are primarily composed of animal-based foods, have gained popularity in recent years. These diets emphasize high protein and fat intake while minimizing carbohydrates, and their effects on metabolism have been the subject of various studies, as this type of diet has been linked to different pathologies. For instance, previous literature related red and processed meat consumption to insulin resistance, metabolic syndrome, type 2 diabetes, oxidative stress, and non-alcoholic fatty liver disease (NAFLD) [178,179,180,181,182].

Metabolomics has gained significant attention for its ability to provide insights into the biochemical processes influenced by various diets. Understanding the metabolomic profile of individuals on carnivorous diets is essential, as it can reveal the metabolic impacts of consuming a meat-rich diet and help identify potential health risks and benefits. Several studies have investigated the metabolomic profiles of populations consuming meat-rich diets. These studies aim to identify specific metabolites associated with high meat consumption and understand the metabolic pathways affected by such diets. Hence, nutritional metabolomics primarily aims to identify specific metabolites linked to food intake and their effects on the risk of chronic diseases. For example, research in this field has uncovered the atherogenic compound trimethylamine N-oxide (TMAO). TMAO is generated by the gut microbiome from dietary components like choline, betaine, and L-carnitine, which are commonly found in foods such as eggs, red meat, and fish. Thus, TMAO has been associated with higher cardiovascular disease development [183,184]. Moreover, the current literature has proposed how L-carnitine, abundant in red meat, is metabolized by gut microbes into TMAO, with JAGTTR01 sp018223385 confirmed as a key contributor. Researchers analyzed microbial genes in individuals with different TMAO production levels and identified the gbu gene cluster as the primary driver, rather than cntA/B or the cai operon. Previously, it was believed that the cntA/B genes or the cai operon were the main drivers of TMAO production. Additionally, this study proposed that gbu gene abundance increased with carnivorous diets and decreased with plant-based diets. These findings suggest that gbu-containing microbes could serve as biomarkers or targets for personalized nutrition and cardiovascular health strategies [185].

Thus, a recent study has explored the effects of high-fat meat protein diets on potential metabolite biomarkers in a mice model, developed for non-alcoholic fatty liver disease (NAFLD). The study showed that diets supplemented with mutton proteins increased hepatic total cholesterol, triglycerides, serum alanine transaminase, and aspartate transaminase, along with lipid droplet accumulation. These changes were accompanied by altered gene expression associated with lipid and cholesterol metabolism. This kind of diet significantly upregulated the expression of acetyl-CoA carboxylase 1 (ACC1), sterol regulatory element-binding protein 1 (Srebf1), fatty acid transporter (CD36), fatty acid synthase (FAS), and stearoyl-CoA desaturase 1 (SCD1). Additionally, the diet increased the levels of sterol regulatory element-binding protein 2 (Srebf2), 3-hydroxy-3-methyl-glutaryl-CoA reductase (HMGCR), and peroxisomal acyl-CoA oxidase 1 (ACoX1). Conversely, it downregulated the expression of cholesterol 7 alpha-hydroxylase (Cyp7a1) and sterol 27-hydroxylase (Cyp27a1), indicating a shift in lipid and cholesterol metabolic pathways [186].

Furthermore, in a recent population-based in Swedish cohort, metabolites associated with processed red meat consumption were correlated with elevated levels of fasting insulin, hemoglobin A1c, and lipoprotein(a). Additionally, these metabolites showed an inverse relationship with maximal oxygen consumption. In contrast, the metabolites linked to unprocessed red meat consumption did not exhibit these specific associations, highlighting the distinct metabolic impacts of processed versus unprocessed red meat. More specifically, unprocessed red meat intake was associated with 403 metabolites, with strong positive and negative links to 1-(1-enyl-stearoyl)-2-arachidonoyl-GPE and glutamine degradants. Processed red meat showed similar associations, notably, with 1-(1-enyl-stearoyl)-2-arachidonoyl-GPE and 2,6-dihydroxybenzoic acid. All these findings may relate higher meat intake to adverse health outcomes, including cardiovascular disease [187].

Comparing the metabolomic profiles of individuals on carnivorous diets with those on predominantly plant-based diets reveals significant differences in specific metabolites and metabolic pathways. Plant-based diets are characterized by higher consumption of fruits, vegetables, grains, legumes, nuts, and seeds, which influence the metabolomic profile differently than meat-rich diets. In this line, a recent study proposed the association of plant-based diet indexes with the metabolomic profile. This study showed that individuals with a higher healthful plant-based diet index had a lower body mass index (BMI), lower fasting glucose levels, and higher high-density lipoprotein cholesterol (HDL-C) levels. In contrast, those with higher unhealthful plant-based diet indexes had a higher BMI, higher triacylglycerol and fasting glucose levels, and lower HDL-C levels. The study identified several amino acids and biogenic amines associated with cardiometabolic diseases, highlighting the beneficial effects of healthful plant-based diets on metabolic health [188].

Moreover, recent researchers described the plasma metabolomic profiles of plant-based dietary indices and their association with metabolic syndrome [188,189]. Hence, the authors proposed how plant-based diets were associated with improved health outcomes, including a lower risk of metabolic syndrome. Among 411 plasma metabolites analyzed, 13 key metabolites were identified, including hypaphorine, indolepropionic acid, and lignan-derived enterolactones, which were connected to all plant-based diet indices and showed an inverse relationship with metabolic syndrome components. Indolepropionic acid, derived from dietary tryptophan, has been linked to improvements in chronic low-grade inflammation and higher dietary fiber intake [190,191]. Enterolactones, produced by gut microbiota from plant lignans, are associated with health benefits in chronic and cardiometabolic diseases [192,193]. These findings highlighted how the metabolomic fingerprints of plant-based diets revealed potential pathways for metabolic syndrome associations, emphasizing the role of plant-based diets in promoting metabolic health.

A diet rich in meat has been linked to various biomarkers associated with protein metabolism. One significant biomarker is blood urea nitrogen (BUN). BUN is produced in the liver when proteins are broken down, and it serves as an indicator of protein intake and kidney function. High-protein diets, like those in carnivorous eating patterns, tend to raise BUN levels due to the increased breakdown of amino acids. Another important biomarker is creatinine, a byproduct of muscle metabolism. Creatinine levels are commonly used to assess kidney function, but they can also indicate muscle mass and protein metabolism. Elevated creatinine levels can be seen in individuals who consume large amounts of animal protein, as their muscle tissue undergoes more frequent breakdown and renewal. Studies have shown that individuals on high-protein diets, such as those following carnivorous diets, often have elevated BUN and creatine levels, reflecting increased protein metabolism [194].

In terms of lipid metabolism, triglycerides and cholesterol are commonly used as biomarkers. Carnivorous diets are typically high in fats, especially saturated fats, which can affect lipid profiles. People who follow a diet rich in animal fats often show elevated levels of triglycerides and cholesterol, particularly low-density lipoprotein (LDL). These lipid biomarkers are linked to the risk of cardiovascular diseases, raising concerns about the long-term health effects of high-fat carnivorous diets [195].

Ketone bodies, especially β-hydroxybutyrate, are another key biomarker in carnivorous diets. When carbohydrate intake is very low, the body enters ketosis, using fat as its main energy source. Carnivorous patterns often show higher levels of ketone bodies, as their bodies adjust to using fats instead of carbohydrates for energy. Elevated ketone bodies clearly indicate lipid metabolism when carbohydrates are not significantly consumed [196].

## 8. Gut Microbiota and Adaptation to a Carnivorous Diet

There have been recent studies that have suggested that microbiota in the gut may play a significant role in the preservation of human health [197]. Even though nutrition research has primarily concentrated on how direct interactions between dietary components and host systems influence human health, it is becoming increasingly vital to take into consideration the effects of nutrients on the microbiome of the gut to obtain a more comprehensive picture [198]. The comprehension of nutrient–host–microbiome interactions has the potential to reveal new mechanisms of disease etiology and progression, as well as provide new disease prevention strategies and therapeutic possibilities, and may require alternative criteria to assess the safety of food constituents [198]. Recent research has revealed how gut microbiotas have helped mammalian species adapt to new carbon sources and specialize in certain foods. Gut microbiotas also amplify environmental and developmental signals, shaping mammalian phenotypic plasticity. These microbiota-induced evolutionary consequences appear to have occurred swiftly amongst closely related mammalian species and communities, possibly affecting recent human evolution [199].

### 8.1. Differences in Microbiota Composition Among Carnivores, Omnivores, and Herbivores

The distinct dietary adaptations of carnivores, omnivores, and herbivores are reflected in the vast differences in the composition of gut bacteria among these three groups [200]. Bacteria that are experts at fermenting and metabolizing carnivorous foods thrive on these diets because they are high in fat and protein and low in fiber. The bacteria that can break down complex carbohydrates and produce SCFAs are more abundant in the guts of omnivores and herbivores [201], especially those who eat a high-fiber diet [202]. Zoelzer et al. showed precisely that herbivores exhibit significantly higher microbial diversity than carnivores, reflecting distinct gut microbiota adaptations [202]. However, unlike herbivores, carnivores show high intra-species variability in microbiota composition, posing methodological challenges in microbiome research. Studies indicate that analyzing fewer than 10 samples per species may yield inconsistent results, whereas from 10 onwards, bacterial composition stabilizes, ensuring more reliable findings [203]. Nevertheless, studies have pointed out that *Clostridium* and *Bacteroides* dominate the protein and fat metabolism microbiota of carnivores [204], which is less diverse and more variable. Their role and that of *Fusobacterium* play crucial roles in these diets:

• *Clostridium perfringens* is associated with protein breakdown through proteolytic fermentation, producing metabolites such as ammonia and hydrogen sulfide [205,206].
• *Bacteroides* species are efficient in hydrolyzing fats and proteins, contributing to lipid metabolism and the production of essential nutrients [207].
• *Fusobacterium* specializes in the degradation of amino acids and peptides, further supporting protein digestion and energy extraction [208].

The predominance of these bacteria in carnivorous gut microbiomes enables efficient nutrient absorption but also results in the production of byproducts such as branched-chain fatty acids and nitrogenous waste, which influence gut health and metabolic homeostasis [209]. However, a recent case study analyzed the gut microbiota of a healthy individual who followed a strict carnivorous diet for four years, consuming only animal-based products [210]. Surprisingly, their microbiota exhibited an alpha and beta diversity comparable to that of healthy omnivores. Additionally, dominant bacterial genera, such as *Faecalibacterium, Blautia*, *Lachnospiraceae* and *Roseburia*, included those typically associated with fiber degradation. These findings suggest that despite the absence of plant-based foods in the diet, the gut microbiota can maintain a composition and functionality similar to that of an omnivorous diet [210]. In particular, individuals following animal-based diets often exhibit an increased abundance of Bacteroides spp. and Clostridia, taxa associated with proteolytic fermentation and amino acid catabolism. Conversely, fiber-rich plant-based diets are linked to higher levels of Prevotella and Ruminococcus, microbes that specialize in carbohydrate fermentation [2]. A key marker in gut microbial composition is the Firmicutes/Bacteroidetes (F/B) ratio, which is known to shift in response to dietary macronutrient profiles. High-protein, low-carbohydrate diets often result in a decreased F/B ratio, favoring Bacteroidetes, which are more adapted to metabolize fats and proteins. This shift is consistent with observations in carnivorous species, where Bacteroides, Alistipes, and Bilophila dominate the microbiota [211].

Functionally, these microbial populations harbor genes encoding proteases, urease, and enzymes involved in amino acid deamination and the production of nitrogenous waste metabolites such as ammonia and TMA (trimethylamine), precursors to TMAO [212]. These enzymatic capabilities reflect an adaptation to protein-centric substrates and may underlie the generation of metabolomic signatures such as branched-chain amino acids and nitrogenous compounds seen in meat-based diets [213].

Regarding omnivores, they have a more diverse microbiota capable of digesting proteins fats and carbohydrates the predominant bacteria, including *Bacteroides*, versatile in fermenting both proteins and carbohydrates; *Prevotella*, associated with carbohydrate, particularly in fiber-rich diets [214]; and *Firmicutes* such as *Lactobacillus* and *Clostridium*, which contribute to both protein and carbohydrate metabolism [215]. Omnivores may digest plant and animal foods with a balanced microbiota of carbohydrate- and protein-fermenting bacteria, giving them more nutritional flexibility [216].

In addition, herbivores have a microbiota specialized in fiber fermentation and short-chain fatty acid SCFA production, which is crucial for energy extraction from plant material. The main bacterial genera include *Ruminococcus*, responsible for breaking down cellulose and complex carbohydrates; *Fibrobacter*, key in fiber degradation; and *Lactobacillus* and *Bifidobacterium*, which produce beneficial SCFAs through fiber fermentation [217]. Based on the above, evolutionary adaptations to dietary niches may affect digestion, metabolism, and health. Concretely, the gut microbiota plays a crucial function in immune regulation, the synthesis of bioactive compounds, and defense against pathogens [218]. Maintaining a balanced gut microbiota is essential for host health, as its disruption has been linked to various chronic diseases, including cardiovascular, hepatic, renal, respiratory, and intestinal disorders. Understanding how animals adapt their gut microbiota throughout their life course and under extreme environmental conditions can provide valuable insights into the microbial modulation of host biology, with potential biomimetic applications for the prevention and treatment of human diseases [218].

### 8.2. Impact of High Fiber Intake on Human Gut Microbiota

While fiber intake is fundamental for gut health in omnivores and herbivores, its impact on individuals with a microbiota adapted to a high-protein, high-fat diet can lead to significant microbial shifts [219]. Dietary fiber promotes the aforementioned growth of *saccharolytic* bacteria such as *Bifidobacterium*, *Lactobacillus*, and *Prevotella*, which are involved in the fermentation of complex carbohydrates and the production of SCFAs. Thus, adequate fiber intake supports gut microbiota diversity, protects the mucus barrier, and reduces pathogen risk. However, a low-fiber diet promotes mucus-degrading bacteria like *Akkermansia muciniphila* and *Bacteroides caccae*, leading to barrier erosion and increased susceptibility to infection [220]. Desai et al. (2016) showed that fiber deficiency shifts microbial metabolism toward host-derived sugars, thinning the mucus layer and triggering immune changes [220]. More concretely, a diet rich in fiber supports gut microbiota diversity and enhances mucus layer integrity, reducing the risk of pathogen infection [221]. Studies using gnotobiotic mouse models shows that chronic fiber deficiency alters microbial metabolism, leading to a shift from dietary polysaccharide fermentation to host mucus degradation. This process results in the increased proximity of luminal bacteria to the intestinal epithelium and immune response alterations, such as increased levels of antimicrobial peptides and inflammation-related gene expression [222].

However, in individuals or species with a microbiota specialized in protein and fat metabolism, a sudden increase in fiber intake may result in transient dysbiosis, altering microbial composition and potentially leading to digestive discomfort [223]. Studies suggest that while carnivores have a lower abundance of fiber-degrading bacteria, gradual exposure to dietary fiber can lead to adaptive microbial changes, increasing the presence of SCFA-producing bacteria and enhancing gut microbial diversity. It has been demonstrated in a human study that the composition of the gut microbiota can be swiftly altered by the short-term consumption of animal-based versus plant-based foods [168]. This alteration is linked to a reduction in the prevalence of *Firmicutes*, which metabolize plant-based polysaccharides, and an increase in the prevalence of bile-tolerant bacteria, including *Alistipes*, *Bacteroides*, and *Bilophila*. The last of these bacteria has the potential to induce inflammatory bowel diseases [2]. Another human study demonstrated that, while a 24-h transition from animal-based to plant-based food can alter the structure of the gut bacteria (from *Bacteroides*, which is associated with animal food, to *Prevotella*, which is linked to carbohydrates), long-term dietary modification is necessary to reinforce the gut bacteria enterotypes [10]. The diet is essential for gut health because it promotes the development of beneficial bacteria, increases microbial diversity, and increases the production of SCFAs. Additionally, experimental evidence indicates that the intestinal microbiota not only influences the absorption and synthesis of nutrients and vitamins [224] but also regulates the absorption of energy from food. For example, research has demonstrated that obesity is associated with a relative abundance of *Bacteroidetes and Firmicutes*, and the microbiome of obese individuals is more efficient in extracting energy from diet. However, the transfer of their gut microbiota to germ-free mice increases the mice’s total body fat [225].

These findings highlight the importance of dietary adaptations in shaping the gut microbiota and suggest that extreme dietary shifts, such as transitioning from a high-protein to a high-fiber diet, may require a period of microbial adjustment for optimal digestion and metabolic function. The findings also highlight that fiber fermentation by gut bacteria leads to the production of SCFAs such as acetate, propionate, and butyrate, which contribute to maintaining intestinal homeostasis and reducing inflammation [132].

## 9. Hormonal Regulation and Meat Consumption

Hormones play a crucial role in regulating metabolism, appetite, and energy balance, and diet is a key factor influencing their secretion and function. A carnivorous diet, characterized by high protein intake and minimal carbohydrates, has significant effects on hormonal pathways, particularly those related to glucose metabolism and appetite control. Low carbohydrate intake significantly impacts insulin and glucagon regulation [226]. Insulin is a key anabolic hormone that facilitates glucose uptake into cells, while glucagon functions antagonistically, promoting glycogen breakdown and gluconeogenesis to maintain blood glucose levels [171].

Research suggests that insulin secretion can be partially stimulated by a high protein diet, but not to the same extent as carbohydrate consumption [227]. Nevertheless, the insulin response to protein is contingent upon the amino acid composition, with certain amino acids (e.g., leucine and arginine) promoting insulin secretion [228]. However, Kalogeropoulou’s study concluded that leucine supplementation, at a dose comparable to that found in a high-protein meal, led to an increase in glucagon levels without significantly affecting serum glucose or insulin concentrations. In this regard, when leucine was consumed alongside glucose, there was a notable attenuation of the glucose response, accompanied by a significant increase in insulin secretion [229]. Additionally, leucine prevented the expected decrease in glucagon levels that typically occurs with glucose intake alone. These findings suggest that leucine requires a substantial rise in glucose concentration to trigger significant insulin secretion, which, subsequently, modulates the body’s glycemic response to ingested glucose [229]. Regarding arginine, Krause et al. demonstrated that might enhance β-cell insulin secretion, promote antioxidant and protective responses, and improve the functional integrity of β-cells and islets in the presence of proinflammatory cytokines [230,231].

Regarding its antagonistic function, the body primarily relies on gluconeogenesis to maintain blood glucose levels in the absence of significant carbohydrate intake, which is why a carnivorous diet tends to increase glucagon production [232]. This change facilitates metabolic adaptations, including ketogenesis and increased fat oxidation, which are prevalent in low-carb, high-protein diets [175]. Dietary ketosis is a physiological condition that is harmless and results from the regulated and controlled production of ketone bodies during periods of extremely low carbohydrate intake. Ketone bodies are transported from the liver to extra-hepatic tissues (e.g., the brain) for fuel consumption [164]. This process is analogous to the oxidation of fatty acids as an alternative, which conserves glucose metabolism [164]. Thus, ketone bodies are a highly effective respiratory fuel in comparison to glucose [164]. Intriguingly, the effects of ketone body metabolism indicate that moderate ketosis may possess therapeutic potential in a diverse array of common and rare disease states. Additionally, a recent study carried out by Manninen et al. demonstrated that a very-low-carbohydrate diet led to a substantial decrease in adipose mass and a corresponding increase in lean body mass [233]. However, though a reduction in carbohydrate consumption may decrease insulin demand, the long-term maintenance of a meat-based diet may impact insulin sensitivity. Zelber-Sagi et al. exposed that numerous diseases are associated with excessive meat [234]. Furthermore, the prolonged heating of meat at elevated temperatures results in the formation of heterocyclic amines, which have adverse health consequences. The formation of non-alcoholic fatty liver disease is significantly correlated with insulin resistance, and it is a substantial public health burden [171]. Kim et al. also concreted that a dietary pattern rich in red and processed meats and refined grains diminished insulin sensitivity in comparison to a dietary pattern abundant in whole grains, nuts, dairy products, and legumes, but only among relatively insulin-resistant adults [235]. This may be attributed to factors such as heme iron, advanced glycation end products (AGEs), and saturated fat ingestion.

### 9.1. Regulation of Leptin and Ghrelin in Response to High-Protein Diets

A high-protein diet, such as a carnivorous diet, influences appetite regulation by modulating leptin and ghrelin secretion. Leptin, which signals satiety, becomes more effective in high-protein diets due to increased sensitivity, but since its levels depend on fat mass, individuals with reduced body fat may experience lower leptin levels, potentially leading to increased appetite [236,237]. Limited research has been undertaken on the correlation between dietary protein and leptin levels in humans. A study carried out by Weigle et al. including healthy participants revealed that an isocaloric high-protein meal did not affect serum leptin levels but enhanced self-reported fullness. Moreover, an ad libitum high-protein meal led to reduced circulating leptin levels, as well as diminished calorie intake, body weight, and fat mass [238].

Conversely, ghrelin, the hunger-stimulating hormone, is significantly suppressed by high protein intake, promoting prolonged satiety and reduced caloric intake [151]. While these effects support short-term weight loss and appetite control, long-term hormonal adaptations may impact metabolism, with persistent ghrelin suppression and low leptin levels potentially making sustained weight management more difficult [239]. Research indicates that the predominant diet abundant in fruits, vegetables, and protein sources had a favorable correlation with ghrelin, and adherence to a diet abundant in animal proteins was linked to reduced BMI and blood pressure. However, a diet high in refined carbohydrates and snacks was linked to reduced adiponectin levels and may lead to insulin resistance and cardiometabolic risk [240].

### 9.2. Influence on Energy Metabolism and Weight Homeostasis

Elevated meat consumption correlates with heightened saturated fat intake and diminished. Consequently, meat consumption is associated with obesity, cardiovascular disease, metabolic syndrome, and gastrointestinal malignancies. Animal-based diets are characterized by elevated levels of leucine and other necessary amino acids, which are linked to enhanced anabolic potential and muscle protein synthesis [241]. Numerous research indicates contradictory findings about the actual advantages of animal-based diets, implying that overall protein consumption may be the most reliable indicator for maintaining lean muscle mass and enhancing muscle function [241]. One of the primary metabolic shifts observed in individuals following a high-protein, low-carbohydrate diet is an increased reliance on fat oxidation and ketogenesis [172]. With limited carbohydrate availability, the body adapts by breaking down stored fat for energy, leading to higher ketone production.

Elevated glucagon levels further support this process by promoting lipolysis and reducing fat storage, facilitating fat loss while preserving lean muscle mass [242]. Additionally, Halton exposed many years ago that protein has a high thermogenic effect, meaning its digestion and metabolism require more energy compared to carbohydrates and fats [243]. This results in an increased metabolic rate, which can aid in weight maintenance and reduce fat accumulation. The combination of enhanced fat oxidation and the thermic effect of protein contributes to improved body composition, as studied by Guarnieri et al. Higher protein meals/diets increase components of energy expenditure [244], so more recently, high-protein diets have been shown to reduce fat mass while maintaining or even increasing muscle mass [245]. This is particularly beneficial for metabolic health and long-term weight control [168]. However, excessive protein intake without proper caloric balance may lead to unintended weight fluctuations. In fact, Hernandez-Alonso et al. remarked that elevated dietary protein consumption correlates with a long-term heightened risk of weight gain and overall mortality in a Mediterranean cohort at elevated cardiovascular risk [246].

While the short-term benefits of a carnivorous diet include increased satiety, muscle retention, and fat loss, the long-term metabolic implications remain a subject of research. Prolonged dependence on high-protein, meat-based diets may pose challenges related to nutrient balance, gut microbiota composition, and potential metabolic stress [247]. Some studies suggest that excessive consumption of red and processed meats could contribute to metabolic disorders if not properly regulated, emphasizing the need for further investigation into the long-term effects of this dietary approach [27,248].

## 10. Anthropological Evidence of Scavenging in Early Humans

Archaeological investigations have documented compelling evidence that early hominins engaged in scavenging behaviors, including the consumption of large felids—most likely lions—approximately 1.84 million years ago at Olduvai Gorge. Cut marks on bones indicate that early humans gained access to the carcasses only after primary carnivores had already fed, suggesting a secondary acquisition of meat. This pattern points to the ecological proximity of early hominins to large carnivore guilds and supports the hypothesis of a mixed subsistence strategy involving both hunting and scavenging [249].

Computational modeling studies have further examined how early hominins competed with other scavengers, such as giant hyenas, for access to carcasses abandoned by apex predators like saber-toothed cats. These simulations suggest that maintaining an optimal group size was critical for successful carcass acquisition. Moreover, the availability of nutrient-rich remains may have played a pivotal role in facilitating the dispersal of early Homo species from Africa into Eurasia between approximately 1.4 and 0.8 million years ago [249,250].

Additional lines of evidence indicate that, around 2 million years ago, early human ancestors adopted confrontational scavenging strategies—also known as “power scavenging”—in which they actively contested carcasses with competing carnivores rather than passively collecting remains. The sequence of bone surface modifications, particularly cut marks superimposed over tooth marks, supports this behavior by suggesting that hominins accessed carcasses prior to other scavengers [250,251,252].

Nevertheless, some researchers have challenged the evidence for scavenging among Neanderthals and early anatomically modern humans, citing taphonomic biases in faunal assemblages from archaeological sites. These biases may have led to misinterpretations favoring scavenging over hunting, which, in certain contexts, was likely the primary mode of animal resource acquisition [253,254].

Despite the debate, scavenging likely provided significant nutritional advantages to early hominins, particularly in terms of access to animal-derived proteins and lipids, which were crucial for brain and body growth. Furthermore, this behavior may have shaped social complexity and group coordination, as collective effort was often required to compete effectively with other scavengers [250,255,256]. While scavenging was not a behavior exclusive to early humans—being common among various carnivores—it likely played a foundational role in shaping the evolutionary trajectory of the genus *Homo*.

### 10.1. Analysis of Feeding Strategies in Early Hominins

Feeding strategies in early hominins provide crucial insights into the adaptive pathways that shaped human evolution. Recent research emphasizes the dietary diversity and ecological flexibility exhibited by early hominin species, along with the socioecological factors that influenced their subsistence behaviors [257]. The following analysis synthesizes key findings from contemporary studies:

Early hominins likely employed opportunistic feeding strategies. Observational studies of wild chimpanzees reveal behavioral parallels, particularly among juveniles, who self-supplement their diets by consuming readily accessible, nutrient-rich resources such as marrow and decayed wood. This behavior may have enabled young hominins to meet their metabolic demands while avoiding the risks associated with foraging at a distance from maternal protection [258,259].

Extractive foraging, especially the excavation of underground storage organs such as roots and tubers, appears to have been a significant component of early hominin diets. These carbohydrate-rich foods were abundant in open habitats and offered a reliable energy source even under challenging environmental conditions. The practice of extractive foraging also required tool use and may have facilitated intra-group food sharing, thereby fostering cooperative behavior [58,260].

Species such as *Homo* and *Paranthropus* demonstrated remarkable dietary flexibility across a range of paleoenvironments. Stable isotope analyses suggest that their diets incorporated both C3 plant resources (e.g., fruits and leaves) and C4 resources (e.g., tropical grasses and sedges) [3,261,262]. This dietary versatility would have enabled them to thrive in ecotones ranging from wooded savannas to open grasslands.

Food sharing emerged as a key behavioral adaptation among early hominins, particularly regarding extracted plant foods. Sharing likely evolved as a cooperative strategy that enhanced group survival and cohesion. For instance, females were observed to share food with offspring and unrelated males under certain mating systems (e.g., pair bonding), potentially laying the groundwork for the emergence of complex social behaviors [260,263].

Certain australopithecines developed morphological adaptations, including robust jaws and thick molar enamel, which were suited for processing hard foods such as nuts and seeds. However, microwear analyses and isotopic data suggest that these items did not consistently dominate their diets, indicating a broader dietary repertoire than previously assumed [3,264,265,266,267].

In sum, early hominins exhibited significant adaptive flexibility through opportunistic feeding, extractive foraging, and cooperative food sharing. These strategies not only enhanced survival in variable environments but also played a fundamental role in shaping the evolutionary trajectory of the human lineage.

### 10.2. Evidence of Access to Meat Through Opportunistic Scavenging

Extensive research has provided compelling evidence that early hominins accessed meat through opportunistic scavenging, offering critical insights into their subsistence strategies and ecological adaptations. One of the most direct examples comes from the DS site in Bed I of Olduvai Gorge, dated to approximately 1.84 million years ago. At this site, early hominins scavenged carcasses left behind by large felids, most likely lions. Cut marks on bones indicate that hominins gained access to the remains only after primary carnivores had consumed part of the prey, underscoring the opportunistic nature of their behavior [249].

Across Africa, early hominins are thought to have frequently scavenged defleshed carcasses left by both modern and extinct felids. In southern Europe, they exploited remains left by saber-toothed predators such as *Megantereon* and *Homotherium*, suggesting a degree of ecological flexibility in their scavenging strategies based on regional conditions [268].

Historical and ethnographic studies further support the idea that opportunistic waste collection has been a viable subsistence strategy among hunter–gatherers under specific environmental constraints. Archaeological evidence from sites in South America demonstrates the use of animal remains collected in similar contexts, reinforcing the evolutionary relevance of scavenging behavior [269].

Opportunistic scavenging enabled early hominins to access high-calorie animal-derived resources, such as meat and marrow, which were instrumental for brain and somatic development. This behavior illustrates their adaptability in exploiting available resources according to ecological opportunity. Moreover, it likely influenced social cooperation and technological innovation, as processing scavenged remains often required the use of tools.

### 10.3. Comparison with Other Scavenger Species and Its Evolutionary Relevance

Comparative analysis of early human scavenging strategies and those of other scavenger species reveals important dynamics in human evolution and Pleistocene ecosystems. One key interaction involved competition between hominins and hyenas for carcasses abandoned by saber-toothed felids. Simulations suggest that hominins needed to maintain an optimal group size—approximately 10 individuals—in order to compete successfully with hyenas, which were highly specialized “hypercarnivorous scavengers” [250,270,271].

Lions and saber-toothed tigers frequently left behind carcasses rich in edible tissues, which early hominins accessed through confrontational scavenging—actively contesting access to carcasses with other carnivores. In Africa, they exploited remains abandoned by modern lions, while in Eurasia, hominins competed for carrion with large felid species such as *Megantereon* and *Homotherium* [272,273].

## 11. Metabolomics and Protein Digestion in Humans

Protein digestion involves the breakdown of dietary proteins into amino acids, which are then absorbed and utilized by the body. One important metabolite is blood urea nitrogen (BUN), which is produced in the liver when proteins are broken down. BUN levels can indicate protein intake as well as kidney function. High-protein diets, such as those in carnivorous eating patterns, tend to increase BUN levels due to the increased breakdown of amino acids. Another key metabolite is creatinine, a byproduct of muscle metabolism. Creatinine levels are often used to estimate kidney function but can also reflect muscle mass and protein metabolism. Both elevated creatinine and BUN levels can be observed in individuals consuming large amounts of animal protein, as mentioned above [194]. This dietary pattern compromise health status, as it was highlighted by previous researchers how a raised protein diet may be related to elevated serum urean and urinary calcium excretion in obese or type 2 diabetic subjects, contrasted to a normal or low protein pattern [274]. Moreover, raised meat and processed meat consumption has been linked to inflammatory events as well as cardiovascular diseases [275].

Protein digestion and metabolism generate various byproducts, primarily ammonia and urea. The digestive process for proteins begins in the stomach, where hydrochloric acid enhances denaturalization protein and pepsin then breaks down the protein molecules into smaller peptides. These peptides are then further broken down into amino acids in the small intestine by proteolytic enzymes secreted by the pancreas, such as elastase, carboxypeptidase, trypsin, and chymotrypsin, as well as by aminopeptidase and dipeptidase, secreted by the small intestine. Then, these amino acids are absorbed through the intestinal lining and transported to the liver through hepatic portal vein, where they are used for various metabolic processes [276].

Ammonia is a highly toxic compound produced during the breakdown of amino acids, and its accumulation can be harmful in the organism. To prevent toxicity, the liver converts ammonia into urea through the urea cycle, a crucial metabolic pathway that ensures safe nitrogen elimination. This cycle consists of a series of enzymatic reactions developed in liver cells. It begins with the formation of carbamoyl phosphate from ammonia and bicarbonate in the mitochondria, followed by the synthesis of citrulline. Citrulline is extracted to cytosol, and through further transformations located in this area, urea is produced and then transported to the kidneys for excretion in urine. This process is essential for maintaining nitrogen balance and preventing the harmful effects of ammonia accumulation [277].

The identification of these metabolites is crucial for understanding protein metabolism. Factors such as dietary protein intake influence the efficiency of the urea cycle. High-protein diets, particularly those rich in animal proteins, lead to increased amino acid breakdown, resulting in higher ammonia production and, consequently, greater urea synthesis [278,279]. Thus, if the liver or kidneys are unable to process or eliminate these byproducts efficiently, their accumulation may indicate metabolic stress or impaired organ function [280]. In clinical settings, metabolic markers like BUN and creatinine help assess protein metabolism and organ health. Hence, it has been pointed out by previous researchers thar elevated levels of circulating BUN enhance protein carbamylation and produce reactive oxygen species, which, in turn, increase oxidative stress, inflammation, endothelial dysfunction, and the risk of cardiovascular disease [281].

BUN reflects the efficiency of ammonia conversion and urea excretion, while creatinine, a byproduct of creatine phosphate breakdown in muscle tissue, provides insight into muscle mass and kidney function. Nevertheless, contrary to what has been thought for many decades, a recent meta-analysis suggests that creatine supplementation did not significantly affect serum creatinine levels or plasma urea values. These findings imply that creatine supplementation does not cause renal damage when taken in the studied amounts and durations [282]. These findings suggest controversial results relating to creatine levels. Thus, more research is needed to confirm that high creatine intake may have a negative impact in general health status.

Amino acids derived from protein digestion are essential nutrients for the growth and maintenance of organisms. Mammals can synthesize approximately half of the 20 proteinogenic amino acids, while the other half are essential and must be acquired from the diet. The absorption of amino acids is mediated by a set of specific amino acid transporters, along with the transport of di- and tripeptides. This process depends on the type of amino acids, as it has been described different types of transporters, depending on the charge (neutral, cationic, or anionic amino acids), as well on the size of amino acid (transport of glycine, proline, and beta amino acid, dipeptides, and tripeptides). These transporters provide amino acids for systemic needs and for enterocyte metabolism. Absorption is largely complete by the end of the small intestine, where most amino acids are taken up. The large intestine also plays a role in mediating the uptake of amino acids derived from bacterial metabolism and endogenous sources [283].

The absorption of amino acids can vary significantly between carnivorous and mixed diets. Thus, there are significant differences in amino acid composition among various plant-derived proteins or plant-based protein sources. Carnivorous diets, which are high in animal proteins, tend to provide a more complete profile of essential amino acids compared to mixed diets that include plant proteins [284,285]. In contrast, most plant proteins have a lower content of essential amino acids and are often deficient in one or more specific amino acids, such as lysine and methionine [284]. Regarding absorbability, it has been described that animal proteins are generally more easily digested and absorbed than plant-based protein, leading to a higher bioavailability of amino acids [286]. Additionally, plant proteins often contain antinutritional factors that can inhibit protein digestion and amino acid absorption, including fiber and tannins [287].

Moreover, recent research has shown that the ingestion of plant proteins, such as soy and wheat, results in lower post-meal muscle protein synthesis compared to an equivalent amount of animal protein [288]. The reduced anabolic properties of plant-based proteins can be attributed to differences in protein digestion as mentioned above, but also to amino acid absorption kinetics. Thus, previous studies have proposed how the absorption speed of dietary amino acids varies with the type of protein ingested, affecting postprandial protein synthesis, breakdown, and deposition. In one study, healthy adults consumed a single meal containing two intrinsically 13C-leucine milk proteins, casein (CAS) and whey protein (WP). WP caused a rapid but short increase in plasma amino acids, while casein (CAS) led to a prolonged moderate increase. CAS inhibited whole body protein breakdown by 34%, unlike WP. Postprandial protein synthesis was higher with WP (68%) compared to CAS (31%). Leucine oxidation was lower with CAS, resulting in a more positive net leucine balance. Thus, protein digestion speed significantly impacts whole body protein anabolism, with slow and fast proteins modulating the metabolic response [289]. These results enhance the importance of this difference in amino acid absorption, as it may impact overall protein metabolism and muscle synthesis.

## 12. Metabolomics of Lipid Metabolism in Carnivorous Diets

Carnivorous diets, characterized by an almost complete restriction of carbohydrates and a predominant consumption of animal products, induce profound metabolic adaptations. One of the most interesting areas is the reprogramming of lipid metabolism, evidenced by state-of-the-art metabolomic techniques. This chapter explores how ketone bodies and fatty acids become major sources of energy, how lipid synthesis and oxidation are regulated in hyperproteic contexts, and what metabolomic profiles emerge in states of prolonged physiological ketosis.

### 12.1. Utilization of Ketone Bodies and Fatty Acids as Primary Energy Sources

Carnivore diets represent a subset of ketogenic dietary strategies characterized by a high intake of animal-based products and the partial or complete exclusion of plant-derived foods [290]. These dietary patterns have gained increasing popularity in recent years [291], leading to notable shifts in nutritional behavior. Ketogenic diets have demonstrated beneficial effects on blood pressure and other cardiovascular risk factors, particularly through mechanisms related to weight loss [292]. These trends have garnered scientific interest due to their potential impact on lipid metabolism and overall population health. Through metabolomic profiling, a specific array of bioactive metabolites has been identified, emerging both as dietary biomarkers and as potential mediators of cardiometabolic risk and broader health outcomes [293]. Carnivore diets, especially those rich in red and processed meats, exert a profound influence on lipid metabolic pathways. Within this context, ketone body metabolism emerges as a central node in physiological energy homeostasis, highlighting the need to identify metabolite signatures that can serve as reliable dietary biomarkers [294]. Ketone bodies are primarily synthesized in the liver from acetyl-CoA generated through β-oxidation, and are subsequently distributed to peripheral tissues for oxidation, enhancing the bioenergetic availability of fatty acids [295]. These molecules are intricately linked to fundamental metabolic networks in mammals, including fatty acid oxidation (FAO), the tricarboxylic acid (TCA) cycle, gluconeogenesis, de novo lipogenesis (DNL), and sterol biosynthesis [296].

Ketone bodies and fatty acids demonstrate optimal utilization as primary energy substrates under various physiological and pathological states. In advanced heart failure, ketone oxidation is upregulated as a compensatory mechanism while fatty acid metabolism is downregulated [297,298,299]. During prolonged exercise, ketone bodies enhance endurance capacity by shifting substrate preference away from carbohydrates and fatty acids [300,301]. In hypoglycemia, ketone bodies effectively substitute for glucose, preserving cognitive function and hormonal counter-regulation [302]. In the developing brain, both ketone bodies and fatty acids serve as critical energy substrates supporting cellular growth and differentiation [303]. Under ketotic conditions, such as fasting or adherence to a ketogenic diet, fatty acid oxidation predominates, sometimes at the expense of ketone utilization [304]. Additionally, in glioblastoma models, both fatty acids and ketone bodies are metabolized to sustain tumor growth, indicating a flexible substrate usage [305]. Across these contexts, ketone bodies frequently emerge as efficient and adaptive energy substrates during metabolic stress or altered homeostasis [306].

In the context of carnivorous diets, characterized by minimal carbohydrate intake, the human body undergoes profound metabolic adaptations to utilize fatty acids and ketone bodies as its primary energy sources. During states of reduced glucose availability, such as fasting or high-protein, high-fat diets, fatty acids are mobilized from adipose tissue and transported to the liver. There, they undergo β-oxidation within hepatic mitochondria, yielding acetyl-CoA. When the capacity of the tricarboxylic acid (TCA) cycle is saturated, excess acetyl-CoA is diverted toward ketogenesis, resulting in the production of ketone bodies such as β-hydroxybutyrate and acetoacetate. These ketone bodies are released into the bloodstream and taken up by peripheral tissues, including the brain, where they serve as efficient alternative fuel substrates [307]. Beyond energy provision, ketone bodies participate in cellular signaling and modulate inflammatory and oxidative processes, highlighting their relevance in maintaining energy homeostasis under carbohydrate-restricted conditions, as seen in carnivorous dietary patterns [308]. Under such conditions, the body’s reliance on ketone bodies (i.e., β-hydroxybutyrate, acetoacetate) and free fatty acids (FFAs) increases substantially to meet energetic demands. Hepatic mitochondrial β-oxidation is markedly upregulated, driving an accumulation of acetyl-CoA beyond TCA cycle capacity, thereby enhancing ketogenesis [309]. In peripheral tissues, ketone bodies are metabolized through enzymatic pathways that generate two molecules of acetyl-CoA from β-hydroxybutyrate, which subsequently enter the TCA cycle. Notably, ketone bodies may yield more energy than glucose owing to their elevated chemical potential; the high ΔG_3_ of ATP hydrolysis under ketotic conditions, driven by the redox properties of β-hydroxybutyrate, contributes to enhanced mitochondrial efficiency [310].

During ketosis, insulin typically activates key enzymes involved in pathways that promote the storage of carbohydrate-derived energy. In the absence or severe restriction of dietary carbohydrates, the resulting decline in circulating insulin levels leads to a downregulation of lipogenic pathways and reduced fat accumulation. After several days of fasting or a substantial reduction in carbohydrate intake (typically below 50 g/day), glucose reserves become insufficient to support both the normal oxidation of fatty acids, due to limited oxaloacetate availability in the tricarboxylic acid (TCA) cycle, hence the adage “fat burns in the flame of carbohydrates”, and the obligatory glucose supply required by the CNS [311]. Metabolomic studies have revealed sustained elevations in plasma β-hydroxybutyrate concentrations (>1 mmol/L), along with distinct acylcarnitine profiles indicative of increased fatty acid oxidation (FAO) overload, reflecting a systemic metabolic shift toward lipid-based energy metabolism [312].

During sustained carbohydrate restriction, as occurs in ketogenic or carnivorous diets, the human body undergoes a profound metabolic shift that favors the utilization of fatty acids and ketone bodies as primary energy substrates. Elevated plasma levels of β-hydroxybutyrate (βHB) reflect a marked upregulation of hepatic ketogenesis, wherein fatty acids mobilized from adipose tissue are oxidized in the mitochondria to produce acetyl-CoA. When acetyl-CoA production surpasses the oxidative capacity of the tricarboxylic acid (TCA) cycle, the excess is diverted toward ketone body synthesis. Once released into the bloodstream, these ketone bodies are taken up by peripheral tissues, such as skeletal muscle, the heart, and the central nervous system, where βHB is converted back to acetoacetate and subsequently to acetyl-CoA, fueling the mitochondrial respiratory chain. This mechanism is metabolically efficient, yielding a higher ATP/O_2_ ratio than glucose or long-chain fatty acids, and generating fewer reactive oxygen species. As a result, the metabolomic profiles of individuals in physiological ketosis exhibit sustained elevations in βHB alongside reductions in glucose, pyruvate, and lactate, characterizing a lipid-dominant bioenergetic phenotype [313]. β-Hydroxybutyrate (βOHB), long regarded as an energy carrier from liver to peripheral tissues during fasting or exercise, has more recently been identified as a signaling molecule. βOHB acts via extracellular receptors and serves as an endogenous inhibitor of histone deacetylases (HDACs). These findings support a model in which βOHB functions as a molecular link between environmental factors—such as diet—and gene expression via chromatin modification [314].

### 12.2. Regulation of Lipid Synthesis and Oxidation in Response to High-Meat Diets

Carnivorous diets strongly inhibit hepatic de novo lipogenesis (DNL), regulated by insulin, SREBP-1c, and ChREBP. Low insulin signaling represses acetyl-CoA carboxylase (ACC) and fatty acid synthase (FAS) activity, while PPARα and CPT1A are activated, promoting lipid oxidation [315,316]. In humans, metabolomic quantification has revealed a reduction in malonyl-CoA and plasma citrate, key metabolites of DNL, along with an elevation in acylcarnitines and ketone bodies [317]. These findings correlate with a decrease in RQ (respiratory quotient). A metabolic dependence on lipids at rest and during exercise was visualized in this study. Aerobic endurance exercise performed by well-trained cyclists was not compromised by four weeks of ketosis. This was achieved by a dramatic physiological adaptation that conserved limited carbohydrate stores (both glucose and muscle glycogen) and made fat the predominant muscle substrate at this submaximal power level [318].

The study by [319] demonstrates that exogenous elevation of ketone bodies, particularly β-hydroxybutyrate (βHB), has significant metabolic effects on the regulation of lipid metabolism in humans. Oral administration of ketone esters caused a sustained decrease in plasma levels of free fatty acids (FFA), triglycerides (TG), and glucose, even in the postprandial state, suggesting an acute inhibition of lipolysis and a reduction in the availability of lipogenic substrates. This modulation occurs independently of the dietary state and is mediated in part by the action of βHB on receptors in adipose tissue (such as GPR109A), suppressing lipid mobilization [319].

In the context of carnivorous diets, characterized by high protein and fat intake and low carbohydrate availability, these findings indicate that the high and sustained presence of ketone bodies can act as a negative regulator of endogenous lipid synthesis and as a metabolic signaler that enhances fatty acid oxidation by decreasing the need for hepatic re-esterification. Thus, exogenous ketone supplementation under conditions of high meat intake could amplify the oxidative responses of lipid metabolism characteristic of physiological ketosis. In the state of physiological ketosis, characterized by elevated plasma β-hydroxybutyrate (βOHB) levels, epigenetic adaptations emerge that are measurable through specific metabolomic profiles. Shimazu et al. (2013) demonstrated that βOHB acts as a selective endogenous inhibitor of class I histone deacetylases (HDAC1 and HDAC2), resulting in a widespread increase in histone acetylation, particularly at residues H3K9 and H3K14 [320]. This epigenetic modification activates the transcription of genes associated with resistance to oxidative stress, such as FOXO3A and MT2, whose pathways are enriched in key tissues such as the kidney. The correlation between circulating levels of βOHB and histone acetylation suggests an integrative role of ketone bodies as sensor metabolites linking cellular energy status with gene regulation. From a metabolomic perspective, this type of adaptation translates into a distinctive profile characterized not only by elevated ketonemia, but also by indirect biomarkers of epigenetic modulation and reduced oxidative damage, reinforcing the concept that physiological ketosis activates protective pathways beyond energy provision.

### 12.3. Metabolomic Profiles of Individuals in Physiological Ketosis

In states of physiological ketosis induced by carnivorous or ketogenic diets, metabolomic profiling consistently reveals a distinct biochemical signature. This includes elevated circulating levels of β-hydroxybutyrate, acetoacetate, acetone, and medium-chain acylcarnitines, alongside marked reductions in plasma glucose, pyruvate, and lactate concentrations [321,322]. Both untargeted and targeted metabolomics platforms have enabled the differentiation between physiological and pathological ketosis. For instance, individuals undergoing ketosis through fasting or nutritional intervention exhibit compensated elevations in BCAAs and glutamine, without concurrent increases in potentially toxic ketone intermediates, thereby distinguishing between adaptive and dysregulated metabolic states [323]. Furthermore, hepatic and skeletal muscle metabolomes from animal models of ketosis display a substantial depletion of glycolytic intermediates and a concomitant accumulation of fatty acid oxidation (FAO) derivatives, including 3-hydroxyacyl-CoA and succinyl-CoA [324]. These molecular adaptations, indicative of a systemic shift towards lipid-centric energy metabolism, may underlie the emerging therapeutic potential of ketogenic states in conditions such as epilepsy, type 2 diabetes, and neurodegenerative disorders [307].

The study by Bagheri et al. (2020), offers an in-depth characterization of plasma metabolites associated with dietary patterns, including profiles reflective of ketogenic or carnivore-like diets [294]. Through untargeted metabolomic profiling of over 200 metabolites across two large prospective cohorts (the Nurses’ Health Study and the Health Professionals Follow-up Study), the authors identified distinct lipid-based biomarkers strongly correlated with diet quality. In individuals adhering to low-carbohydrate, high-fat dietary patterns, hallmarks of physiological ketosis, there was a marked accumulation of medium- and long-chain acylcarnitines (e.g., C10, C12, C14 species), indicative of enhanced mitochondrial β-oxidation flux. These acylcarnitines serve as transport intermediates facilitating the translocation of fatty acids into the mitochondrial matrix, where sequential oxidation generates acetyl-CoA for ATP synthesis [294]. Elevated plasma levels of these metabolites represent a robust biochemical fingerprint of lipid-dominant energy metabolism. Moreover, the metabolomic signatures associated with high intake of processed meats (a negative component of the Alternate Healthy Eating Index) were enriched in saturated triacylglycerols (TAGs) and lower in unsaturated plasmalogens, whereas diets rich in polyunsaturated fats, such as those from fish or plant oils, were associated with highly unsaturated TAG species. Collectively, these findings support the notion that physiological ketosis yields a reproducible metabolomic profile marked by specific acylcarnitine elevations and lipid subclass remodeling. These molecular adaptations reflect both substrate availability and the metabolic efficiency of lipid oxidation pathways, providing insight into the systemic metabolic shifts induced by carbohydrate restriction [294].

In the context of physiological ketosis, the work of Cotter et al. (2013) provides compelling evidence that ketone bodies, particularly β-hydroxybutyrate (βHB) and acetoacetate, not only serve as alternative energy substrates under carbohydrate-restricted conditions but also exert profound regulatory control over cellular bioenergetics and metabolic signaling [325]. These molecules modulate the mitochondrial redox state (NAD^+^/NADH), enhance energetic efficiency by increasing ATP yield per unit of oxygen consumed, and attenuate reactive oxygen species (ROS) production, particularly in metabolically demanding tissues such as cardiac and skeletal muscle. Metabolomic analyses consistently reveal a characteristic profile in individuals undergoing sustained ketosis, marked by elevated circulating levels of ketone bodies, specific medium- and long-chain acylcarnitines, and the suppression of canonical glycolytic and lipogenic pathways. In addition to their role as fuels, ketone bodies function as epigenetic regulators through the inhibition of histone deacetylases (HDACs) and the activation of G-protein-coupled receptors such as GPR109A, eliciting anti-inflammatory and antioxidant responses.

This distinctive metabolic phenotype, observable in individuals adhering to ketogenic or carnivore diets, reflects a systemic reprogramming of human energy metabolism that is now measurable through high-resolution metabolomic platforms. Supporting this notion, Wedekind et al. (2020) demonstrated, through a randomized cross-over intervention and subsequent observational validation, that intake of red and processed meats significantly elevates both urinary and serum levels of acylcarnitines—specifically, acetylcarnitine (C2:0), propionylcarnitine (C3:0), butyrylcarnitine (C4:0), isovalerylcarnitine (C5:0), and stearoylcarnitine (C18:0) [293]. These metabolites are critical intermediates in the mitochondrial import and β-oxidation of fatty acids, and their accumulation reflects both enhanced lipid substrate availability and increased flux through oxidative pathways. Notably, this metabolomic signature was positively associated with habitual consumption of red meat, but not white meat such as poultry, implicating the high dietary carnitine and saturated fat content of red meats in the selective activation of these lipid catabolic pathways. From a mechanistic perspective, sustained elevations of specific acylcarnitines serve as robust biomarkers of physiological ketosis and enable precise characterization of the prevailing bioenergetic state in individuals metabolically adapted to fat oxidation, insights increasingly accessible through advanced mass spectrometry-based metabolomics.

Finally, we found how carnivorous diets induce a profound reconfiguration of human lipid metabolism. Through the application of advanced metabolomic technologies, distinct biochemical signatures have been identified that are characteristic of physiological ketosis, the suppression of de novo lipogenesis, and the upregulation of lipid oxidation pathways. These adaptive shifts in energy metabolism present a promising landscape for therapeutic interventions across a range of metabolic and neurological disorders. However, their long-term implications remain underexplored and warrant comprehensive investigation through longitudinal, multicenter studies.

## 13. Impact of a Carnivorous Diet on Modern Human Health

Understanding the impact of high meat consumption on modern human health requires an integrative approach that considers not only clinical and epidemiological data but also evolutionary context and individual metabolic variability. As human diets shift in response to cultural trends and nutritional ideologies, the reemergence of carnivorous and eating patterns focused on meat, such as ketogenic and palaeolithic diets, warrants careful examination. Therefore, metabolomic tools, combined with genetic and physiological markers, now allow for a deeper exploration of how these diets influence health outcomes, moving beyond reductionist paradigms toward personalized nutrition strategies grounded in biology.

### 13.1. Benefits and Risks of High Meat Consumption in Contemporary Diets

High meat consumption in modern human diets elicits polarized interpretations, yet a metabolomic and evolutionary lens provides a better understanding. Meat is an exceptionally nutrient-dense food, offering a high bioavailability of essential nutrients including complete proteins, vitamin B12, niacin, iron, zinc, and creatine. These qualities make meat a cornerstone in ketogenic, low-carbohydrate, and elimination-based diets aimed at improving satiety, glycemic control, and mental clarity [326,327].

From a metabolomic perspective, meat-based diets induce significant shifts in circulating metabolites. Notably, ketogenic diets high in animal products elevate β-hydroxybutyrate and acetoacetate biomarkers of enhanced fatty acid oxidation and ketone body metabolism while reducing markers of glycemic instability such as lactate and pyruvate [328]. Moreover, carnivorous diets also modulate amino acid metabolism, increasing levels of BCAAs and, potentially, enhancing muscle protein synthesis [329]. However, risks have been reported in the epidemiological literature, particularly concerning the long-term consumption of processed meats. Elevated intake of nitrates, heme iron, and advanced glycation end-products has been linked to oxidative stress and inflammation [330]. Additionally, concerns over compounds like trimethylamine N-oxide (TMAO) produced by gut microbes metabolizing meat-derived choline have prompted speculation about increased cardiovascular risk. Yet these effects are strongly modulated by gut microbiota composition, and causal pathways remain unclear [331]. Crucially, population-level dietary data often fail to disentangle high meat intake from confounding factors such as high intake of refined carbohydrates, seed oils, and sedentary behavior. Thus, the risks of meat consumption must be contextualized within broader dietary patterns.

### 13.2. Relationship with Metabolic and Cardiovascular Diseases

The association between meat consumption and non-communicable diseases, particularly cardiovascular disease and metabolic syndrome, has long been contested. While observational studies frequently suggest a positive correlation between red meat intake and the incidence of coronary artery disease or insulin resistance, emerging evidence from metabolomics and clinical interventions suggests this relationship is more complex and non-linear.

When consumed within whole-food, low-carbohydrate dietary frameworks, meat-based diets have been associated with improved metabolic markers. Studies on Paleolithic-type diets show significant reductions in triglycerides, improved HDL profiles, and better insulin sensitivity, even with relatively high saturated fat content [332]. Similarly, ketogenic diets, which often rely heavily on meat and animal fats, have demonstrated therapeutic potential in managing type 2 diabetes, obesity, and polycystic ovary syndrome, conditions rooted in metabolic dysfunction [333]. Metabolomic studies reinforce these findings by showing favorable shifts in lipid and energy metabolism. Decreased levels of lipotoxic intermediates (e.g., ceramides) and increased mitochondrial beta-oxidation markers suggest enhanced metabolic flexibility in individuals on meat-centric diets [328]. Nevertheless, excess caloric intake, particularly from highly processed meats, may still promote low-grade inflammation and contribute to obesity in the absence of carbohydrate restriction or physical activity [330]. The interplay of insulin resistance, endothelial dysfunction, and inflammation in cardiovascular disease etiology implies that meat is only one component among many influencing cardiometabolic risk. Therefore, the evidence supports a differentiated stance: Unprocessed, high-quality meat consumption within carbohydrate-controlled, nutrient-rich diets does not increase cardiometabolic risk and contributes positively to health outcomes.

### 13.3. Individual Adaptability and Genetic Differences in Meat Digestion

The impact of meat-heavy diets is also influenced by genetic and epigenetic variability in digestion, lipid metabolism, and nutrient absorption. Several gene loci have been identified as key modulators of dietary response, helping to explain interindividual differences in tolerance and risk. Variants of the APOE gene, especially APOE4, have been associated with heightened sensitivity to dietary cholesterol and saturated fats. Individuals carrying this allele may exhibit unfavorable lipid responses to high-meat diets, although outcomes also depend on broader dietary context and insulin sensitivity [334]. In addition, the FADS1 and FADS2 genes, responsible for the desaturation of omega-3 and omega-6 fatty acids, show considerable population-level variation. Some ancestral groups that historically consumed high-meat and low-plant diets exhibit efficient endogenous fatty acid desaturation, suggesting adaptation to low dietary polyunsaturated fat availability [327].

Carnivorous diets may also favor individuals with enhanced ketogenesis-related gene expressions (e.g., CPT1A, PPARα) and efficient gluconeogenic capacity, traits hypothesized to have evolved in arctic and pastoralist populations where carbohydrate availability was historically limited [326]. From a metabolomic standpoint, individuals differ significantly in the production of metabolites like TMAO, uric acid, and ammonia when consuming meat. These differences appear to stem from host–microbiota interactions and gene–environment dynamics, reinforcing the need for personalized dietary recommendations based on genomic and metabolomic profiles [331]. The convergence of metabolomics and nutrigenomics, thus, opens a path toward individualized meat consumption guidelines, moving beyond one-size-fits-all dietary models.

In sum, while high meat consumption has historically been viewed through a lens of risk, emerging metabolomic and genetic evidence invites a more nuanced interpretation. Meat-rich diets can confer metabolic advantages—particularly in carbohydrate-restricted contexts—and align with certain evolutionary adaptations in human physiology. Nonetheless, individual genetic makeup, microbiota composition, and dietary context are critical in modulating health outcomes. As such, the impact of a carnivorous diet on modern human health is best understood not as universally harmful or beneficial, but as contingent on biological individuality and overall dietary patterns.

## 14. Implications for Contemporary Nutrition

The evolutionary discordance hypothesis posits that numerous contemporary chronic diseases, including obesity, type 2 diabetes, and select cardiovascular disorders, may have originated from the pronounced discrepancy between the dietary patterns of Paleolithic hunter–gatherers and the highly processed diets prevalent in industrial societies [335]. Observational studies and clinical trials have demonstrated that adopting dietary patterns inspired by ancestral diets can lead to metabolic improvements. These dietary patterns advocate for the consumption of lean meats, fish, vegetables, fruits, and nuts, while eschewing ultra-processed and refined sugars [332,336]. However, the application of these principles to contemporary nutrition is not without nuances and controversies, particularly when addressing the enormous diversity of foods and human genetic and cultural evolution.

In this regard, the majority of Paleolithic dietary interventions implemented in clinical settings have been relatively brief (typically ranging from 3 to 12 months) and have involved modest sample sizes [337,338]. Nonetheless, these studies have demonstrated discernible trends toward reduced body weight, enhanced glycemic control, and diminished inflammatory markers [339]. The underlying mechanisms of these beneficial changes have been attributed to various factors, including an increased intake of high biological value proteins and a marked reduction in refined carbohydrates. Increased insulin sensitivity may be related to the lower glucose overload and the presence of a food matrix richer in micronutrients, antioxidants, and soluble fiber, provided that the paleo version includes sufficient vegetables [340]. Conversely, recent studies have indicated that a reduction in the consumption of ultra-processed foods and artificial sweeteners can positively influence systemic inflammation, a pivotal component in the pathophysiology of metabolic syndrome [341,342].

Conversely, metabolomics has yielded more precise information regarding the biochemical effects of disparate dietary patterns. A comprehensive analysis of metabolites in body fluids reveals significant changes in fatty acid utilization and ketone body synthesis when the diet is oriented towards a majority protein and fat intake [343]. In the short term, some of these changes have been associated with an improvement in body composition and lipid profile, albeit with detectable increases in LDL cholesterol in certain individuals [344,345]. This observation underscores the necessity for further research to ascertain whether these alterations in plasma lipids constitute a genuine cardiovascular risk or are merely a transient metabolic change compensated for by factors such as an increase in HDL cholesterol or a positive modulation of inflammation.

Moreover, the adoption of strict carnivorous diets has been proposed as a radical method to revert to purported human hunting and scavenging patterns. A cross-sectional study of more than 2000 participants following diets based exclusively on meat and animal products showed that many described benefits in weight reduction, glycemic regularization, and overall well-being. However, the study also reported elevations in LDL cholesterol, fiber deficiency, and possible deficiencies in essential micronutrients such as vitamin C and folate, which may have implications for bone, cardiovascular, and gastrointestinal health [346]. The extrapolation of evolutionary biology to everyday practice warrants caution regarding the long-term adherence and safety of restrictions that omit entire food groups. From a clinical standpoint, dietary interventions grounded in evolutionary biology should not be used to justify monothematic and imbalanced dietary patterns.

Conversely, numerous studies have indicated that the most pragmatic nutritional strategy for the present era would entail the incorporation of components from ancestral diets, a heightened nutritional density, the elimination of ultra-processed foods, and the prioritization of quality protein sources [347]. This approach would be informed by the findings of contemporary nutritional epidemiology, which acknowledge the value of select whole grains, legumes, and fermented dairy products in the prevention of chronic diseases [348]. It is plausible that the positive outcomes associated with the Paleolithic diet are predominantly attributable to the elimination of foods with a high degree of refining and additives, rather than to the strict restriction of grains and legumes [349]. In the specific instance of the Mediterranean diet, frequently utilized as a point of reference in epidemiological research, there is a congruence with the perspective concerning the significance attributed to the consumption of fresh foods, healthy fats (e.g., olive oil), and a sufficient intake of vegetables and fruit [350].

The adaptation of individuals to their diets is contingent upon the interplay among genetics, epigenetics, and the composition of the gut microbiota. Evolution has conserved fundamental nutritional needs. However, certain recent mutations reflect a specific biological adaptation. The persistence of lactase and the increase in AMY1 (salivary amylase) gene copies are evidence of metabolic plasticity in populations with an agrarian tradition [351]. These variations may influence tolerance to foods that some strict interpretations of evolutionary diet discourage. Consequently, the clinical application of the evolutionary approach requires a personalized analysis that considers inter-individual variability and promotes an evidence-based nutritional balance.

Therefore, the proposal for contemporary nutrition is appealing to the extent that it promotes the abandonment of ultra-processed foods, free sugars, and trans fats, which are key factors implicated in the growing prevalence of obesity and metabolic syndrome. The emphasis on minimally processed foods, lean meats, and vegetables aligns with research findings that demonstrate objective improvements in cardiometabolic health, particularly in the short and medium term. However, recommendations that advocate for the complete exclusion of food groups should be supported by substantial evidence that validates the significance of plant-based foods, which are abundant in fiber, vitamins, and minerals. The absence of large-scale longitudinal studies and the ambiguity of findings related to lipid profiles underscore the necessity for cautious interpretation. In this line, the application of omics technologies, as well as the monitoring of microbiota, could provide more clarity on which components of the evolutionary diet are truly beneficial and to what extent they are safely applied in contemporary society.

While evolutionary adaptations supported metabolic flexibility during periods of intermittent animal-based food consumption, modern diets often diverge significantly from these ancestral patterns. Contemporary Western dietary habits frequently include continuous, high-volume consumption of ultra-processed red and processed meats, often in the absence of corresponding physical activity levels or dietary fiber intake [352]. This mismatch may contribute to the rise in metabolic disorders, including cardiovascular disease, type 2 diabetes, and colorectal cancer. The type and processing of meat appear to be critical mediators of health outcomes [353,354]. Epidemiological studies consistently associate high consumption of processed meats (e.g., bacon, sausages, deli meats) with increased risk of mortality, metabolic syndrome, and inflammatory diseases. In contrast, moderate intake of unprocessed red meat, when part of a balanced diet, shows more heterogeneous effects, especially when offset by a high intake of fiber and polyphenol-rich plant foods [355].

Therefore, it is important to distinguish between the intermittent, unprocessed, and nutrient-dense carnivory of prehistoric humans—typically shaped by food scarcity and high physical activity—and the continuous, calorie-dense, and chemically preserved meat consumption common in industrialized societies [356]. From a metabolomic perspective, markers such as elevated TMAO, oxidized lipids, and inflammatory cytokines are frequently observed in individuals consuming high levels of processed animal products. These findings underscore the importance of dietary context, quality, and frequency in interpreting the evolutionary legacy of carnivorous adaptations.

## 15. Controversies and Limitations of the Evolutionary Approach

The application of an evolutionary approach to the study of nutrition has given rise to a substantial debate within scientific literature. Several authors emphasize the potential to counteract current chronic diseases by reconnecting with the dietary patterns that dominated much of human evolutionary history, while also pointing to methodological problems, biases in the interpretation of the fossil record, insufficient number of long-term studies, and the inability to capture the true diversity of ancestral diets [340,357,358].

One of the primary challenges pertains to the complexity of directly extrapolating the diet of hunter–gatherers to the present day. The extant archaeological and paleontological record indicates that hominins consume lean meats and fish, but also plants, tubers, and fruits. However, these latter elements are more difficult to trace due to their rapid degradation. This preservation bias, which favors the preservation of meat over plant matter, has led to the underestimation of plant intake in early human diets [359,360]. This has contributed to the prevailing belief that early humans were predominantly carnivorous [361]. Furthermore, the planet exhibits substantial climatic and geographic variability, suggesting the capacity for diverse human groups to adapt their diets to local resources. The supposition of a singular, ancestral diet disregards the preponderance of evidence indicating that flexibility and diversity have been pivotal in the expansion and evolutionary success of *Homo sapiens*. This complicates the establishment of a universal dietary prescription for all human populations.

Another contradictory theory asserts that the human organism has remained genetically static throughout history and, therefore, is not designed to process foods of agricultural origin, such as cereals and dairy products. However, population genetic studies contradict this notion, highlighting recent adaptations [362]. These include the persistence of lactase in adults from populations with a tradition of livestock farming and the increase in the number of copies of the AMY1 gene (salivary amylase) in groups with a high consumption of complex carbohydrates [363]. These modifications, which have occurred in the last few millennia, demonstrate that human evolution has persisted and that a portion of the population can benefit nutritionally from the consumption of milk, cereals, or legumes [151]. Despite the absence of uniformity in these adaptations and their non-inclusion across all ethnic groups, they provide a compelling argument that challenges the rigid interpretation of agriculture as inherently unnatural.

The assessment of health in pre-agricultural times is also a matter of controversy. The apparent rarity of degenerative diseases such as arteriosclerosis or diabetes in hunter–gatherers is often cited as evidence, but it should be noted that life expectancy in these societies was notably lower than in contemporary times [364]. Moreover, lifestyles in these societies were characterized by high physical activity and mortality risks due to infections or trauma. Conversely, certain tumors or cardiovascular pathologies exhibit an increase with age, complicating the comparison with past populations that faced vastly different environmental, health, and demographic conditions [365,366]. Furthermore, the presence of atherosclerotic lesions in ancient mummies indicates that arteriosclerosis may not be exclusive to modern societies.

A notable limitation is the absence of long-term follow-up studies. Numerous clinical trials have demonstrated short-term improvements in cardiometabolic risk factors with evolution-inspired diets [367]. However, no conclusive evidence is available on long-term cardiovascular mortality or cancer. Numerous experts in the field of preventive nutrition argue that the promotion of the exclusion of food groups such as whole grains and legumes is risky [368,369]. This assertion is based on traditional epidemiological evidence that shows that their consumption is associated with a lower incidence of chronic diseases. Similarly, the recommendation to substantially increase animal protein consumption raises questions about environmental sustainability and carbon footprint, an issue that was not considered in the Paleolithic era but now has enormous implications for the future [290].

Furthermore, contemporary Paleolithic diets exhibit variability in their specific components. Proponents of this dietary approach permit the consumption of certain high-glycemic fruits, tubers, and vegetable oils, while others adhere to a strict exclusion of these items [370]. These discrepancies are indicative of the challenges inherent in establishing a universally applicable Paleolithic standard, given the paucity of definitive records and the wide range of ecological conditions that characterized the habitats of hominids [31]. Consequently, ancestral dietary proposals may be susceptible to personal interpretation and lack unified empirical validation.

In the context of diets that are particularly restrictive, such as the carnivorous diet, the level of controversy rises considerably. While some adherents report positive outcomes, including improvements in insulin resistance and weight loss, most professionals express caution [371]. These professionals highlight the absence of fiber, the potential for vitamin deficiencies, the possibility of renal overload, and the increase in LDL cholesterol as significant concerns [372]. Additionally, the potential for a biochemical pattern derived from a high protein and saturated fat intake to augment the risk of atherosclerosis, despite short-term improvements in glycemia, is a subject of discussion [373]. Thus, the rigidity of certain evolutionary interpretations stands in conflict with public health guidelines and the balanced diet proposed by most medical associations, which emphasizes the importance of dietary fiber and a balanced macronutrient intake.

In this regard, research focused on metabolomics and gut microbiota reveals that evolutionary adaptation involves the human genome, as well as co-evolution with bacteria and other microorganisms [374]. It is important to note that any drastic and prolonged dietary modification has the potential to alter microbial composition in complex ways, with as-yet unpredictable long-term physiological and metabolic consequences [375]. Due to the multifaceted nature of the study of evolutionary diet, the most widely accepted recommendation in the literature is to apply the evolutionary perspective with caution and to complement its principles with extensive contemporary evidence from clinical trials, epidemiological studies, and sustainability considerations [376,377].

The evolutionary approach provides significant insights into the importance of reducing the consumption of ultra-processed foods and maintaining a nutritional profile that aligns more closely with natural foods. However, there are important limitations in its application, mainly due to the lack of information. The approach is predicated on a limited paleontological record, underestimates ancestral dietary diversity and post-Paleolithic biological evolution, lacks robust studies on long-term outcomes, and often generates discrepancies with modern epidemiology, which confirms benefits of certain supposedly novel food groups. It is, therefore, imperative to acknowledge these limitations and to adopt a critical and balanced approach in integrating the evolutionary perspective into the broader scientific literature.

## 16. Conclusions

This review highlights the evolutionary trajectory of human digestive physiology and its adaptations to carnivorous and scavenger dietary patterns, drawing on metabolomic, anatomical, and archaeological evidence. The human metabolic system exhibits remarkable flexibility, allowing the efficient utilization of fats and proteins, especially under conditions of carbohydrate scarcity. These adaptations, which once conferred survival advantages, may now contribute to the prevalence of metabolic disorders in modern societies dominated by highly processed foods and sedentary lifestyles.

Metabolomic signatures such as ketone bodies, branched-chain amino acids, and TMAO not only provide insight into ancestral dietary habits but also hold clinical relevance for modern nutritional strategies. While animal-sourced foods played a pivotal role in human evolution, the interplay between such diets and contemporary health outcomes requires nuanced interpretation. Future research integrating personalized metabolomics and evolutionary biology may offer more precise dietary guidelines aligned with human physiological capabilities.

## Data Availability

No new data were created or analyzed in this study. Data sharing is not applicable to this article.

## References

[1] Scalbert A., Brennan L., Manach C., Andres-Lacueva C., Dragsted L.O., Draper J., Rappaport S.M., Van Der Hooft J.J.J., Wishart D.S. (2014). The Food Metabolome: A Window over Dietary Exposure. Am. J. Clin. Nutr..

[2] David L.A., Maurice C.F., Carmody R.N., Gootenberg D.B., Button J.E., Wolfe B.E., Ling A.V., Devlin A.S., Varma Y., Fischbach M.A. (2014). Diet rapidly and reproducibly alters the human gut microbiome. Nature.

[3] Ungar P.S., Sponheimer M. (2011). The Diets of Early Hominins. Science.

[4] Wishart D.S. (2019). Metabolomics for Investigating Physiological and Pathophysiological Processes. Physiol. Rev..

[5] Rothschild D., Weissbrod O., Barkan E., Kurilshikov A., Korem T., Zeevi D., Costea P.I., Godneva A., Kalka I.N., Bar N. (2018). Environment Dominates over Host Genetics in Shaping Human Gut Microbiota. Nature.

[6] Cordain L., Eaton S.B., Sebastian A., Mann N., Lindeberg S., Watkins B.A., O’Keefe J.H., Brand-Miller J. (2005). Origins and Evolution of the Western Diet: Health Implications for the 21st Century. Am. J. Clin. Nutr..

[7] Belinchón-deMiguel P., Navarro-Jiménez E., Laborde-Cárdenas C.C., Clemente-Suárez V.J. (2024). Evolutionary Echoes: A Four-Day Fasting and Low-Caloric Intake Study on Autonomic Modulation and Physiological Adaptations in Humans. Life.

[8] Belinchón-deMiguel P., Ramos-Campo D.J., Clemente-Suárez V.J. (2023). Exploring the Evolutionary Disparities: A Case Study on the Psychophysiological Response to Recreating the Hunter–Gatherer Lifestyle through Physical Activity and Caloric Restriction. Appl. Sci..

[9] Weimbs T., Saville J., Kalantar-Zadeh K. (2024). Ketogenic Metabolic Therapy for Chronic Kidney Disease—The pro Part. Clin. Kidney J..

[10] Wu G.D., Chen J., Hoffmann C., Bittinger K., Chen Y.-Y., Keilbaugh S.A., Bewtra M., Knights D., Walters W.A., Knight R. (2011). Linking Long-Term Dietary Patterns with Gut Microbial Enterotypes. Science.

[11] Milton K. (2003). The Critical Role Played by Animal Source Foods in Human (Homo) Evolution. J. Nutr..

[12] Aiello L.C., Wheeler P. (1995). The Expensive-Tissue Hypothesis: The Brain and the Digestive System in Human and Primate Evolution. Curr. Anthr..

[13] Beasley D.E., Koltz A.M., Lambert J.E., Fierer N., Dunn R.R. (2015). The Evolution of Stomach Acidity and Its Relevance to the Human Microbiome. PLoS ONE.

[14] Guasch-Ferre M., Bhupathiraju S.N., Hu F.B. (2018). Use of Metabolomics in Improving Assessment of Dietary Intake. Clin. Chem..

[15] Speth J.D. (2017). Putrid Meat and Fish in the Eurasian Middle and Upper Paleolithic: Are We Missing a Key Part of Neanderthal and Modern Human Diet?. PaleoAnthropology.

[16] Bocherens H. (2009). Neanderthal Dietary Habits: Review of the Isotopic Evidence. Vertebrate Paleobiology and Paleoanthropology.

[17] Sonnenburg J.L., Bäckhed F. (2016). Diet-Microbiota Interactions as Moderators of Human Metabolism. Nature.

[18] De Filippo C., Cavalieri D., Di Paola M., Ramazzotti M., Poullet J.B., Massart S., Collini S., Pieraccini G., Lionetti P. (2010). Impact of Diet in Shaping Gut Microbiota Revealed by a Comparative Study in Children from Europe and Rural Africa. Proc. Natl. Acad. Sci. USA.

[19] Oellgaard J., Winther S.A., Hansen T.S., Rossing P., von Scholten B.J. (2017). Trimethylamine N-Oxide (TMAO) as a New Potential Therapeutic Target for Insulin Resistance and Cancer. Curr. Pharm. Des..

[20] Koeth R.A., Wang Z., Levison B.S., Buffa J.A., Org E., Sheehy B.T., Britt E.B., Fu X., Wu Y., Li L. (2013). Intestinal Microbiota Metabolism of L-Carnitine, a Nutrient in Red Meat, Promotes Atherosclerosis. Nat. Med..

[21] Solon-Biet S.M., Cogger V.C., Pulpitel T., Wahl D., Clark X., Bagley E.E., Gregoriou G.C., Senior A.M., Wang Q.P., Brandon A.E. (2019). Branched-Chain Amino Acids Impact Health and Lifespan Indirectly via Amino Acid Balance and Appetite Control. Nat. Metab..

[22] Paoli A., Bianco A., Grimaldi K.A. (2015). The Ketogenic Diet and Sport: A Possible Marriage?. Exerc. Sport Sci. Rev..

[23] Roberts L.D., Gerszten R.E. (2013). Toward New Biomarkers of Cardiometabolic Diseases. Cell Metab..

[24] Newgard C.B. (2017). Metabolomics and Metabolic Diseases: Where Do We Stand?. Cell Metab..

[25] Martín-Rodríguez A., Gostian-Ropotin L.A., Beltrán-Velasco A.I., Belando-Pedreño N., Simón J.A., López-Mora C., Navarro-Jiménez E., Tornero-Aguilera J.F., Clemente-Suárez V.J. (2024). Sporting Mind: The Interplay of Physical Activity and Psychological Health. Sports.

[26] Merino M., Tornero-Aguilera J.F., Rubio-Zarapuz A., Villanueva-Tobaldo C.V., Martín-Rodríguez A., Clemente-Suárez V.J. (2024). Body Perceptions and Psychological Well-Being: A Review of the Impact of Social Media and Physical Measurements on Self-Esteem and Mental Health with a Focus on Body Image Satisfaction and Its Relationship with Cultural and Gender Factors. Healthcare.

[27] Clemente-Suárez V.J., Beltrán-Velasco A.I., Redondo-Flórez L., Martín-Rodríguez A., Tornero-Aguilera J.F. (2023). Global Impacts of Western Diet and Its Effects on Metabolism and Health: A Narrative Review. Nutrients.

[28] Clemente-Suárez V.J., Martín-Rodríguez A., Yáñez-Sepúlveda R., Tornero-Aguilera J.F. (2023). Mitochondrial Transfer as a Novel Therapeutic Approach in Disease Diagnosis and Treatment. Int. J. Mol. Sci..

[29] Clemente-Suárez V.J., Navarro-Jiménez E., Ruisoto P., Dalamitros A.A., Beltran-Velasco A.I., Hormeño-Holgado A., Laborde-Cárdenas C.C., Tornero-Aguilera J.F. (2021). Performance of Fuzzy Multi-Criteria Decision Analysis of Emergency System in COVID-19 Pandemic. An Extensive Narrative Review. Int. J. Environ. Res. Public Health.

[30] Clemente-Suárez V.J., Redondo-Flórez L., Beltrán-Velasco A.I., Martín-Rodríguez A., Martínez-Guardado I., Navarro-Jiménez E., Laborde-Cárdenas C.C., Tornero-Aguilera J.F. (2023). The Role of Adipokines in Health and Disease. Biomedicines.

[31] Alt K.W., Al-Ahmad A., Woelber J.P. (2022). Nutrition and Health in Human Evolution–Past to Present. Nutrients.

[32] Scott R.S., Ungar P.S., Bergstrom T.S., Brown C.A., Childs B.E., Teaford M.F., Walker A. (2006). Dental Microwear Texture Analysis: Technical Considerations. J. Hum. Evol..

[33] Sponheimer M., Alemseged Z., Cerling T.E., Grine F.E., Kimbel W.H., Leakey M.G., Lee-Thorp J.A., Manthi F.K., Reed K.E., Wood B.A. (2013). Isotopic Evidence of Early Hominin Diets. Proc. Natl. Acad. Sci. USA.

[34] McPherron S.P., Alemseged Z., Marean C.W., Wynn J.G., Reed D., Geraads D., Bobe R., Béarat H.A. (2010). Evidence for Stone-Tool-Assisted Consumption of Animal Tissues before 3.39 Million Years Ago at Dikika, Ethiopia. Nature.

[35] Byeon W., Domínguez-Rodrigo M., Arampatzis G., Baquedano E., Yravedra J., Maté-González M.A., Koumoutsakos P. (2019). Automated Identification and Deep Classification of Cut Marks on Bones and Its Paleoanthropological Implications. J. Comput. Sci..

[36] Braun D.R. (2010). Australopithecine Butchers. Nature.

[37] Rowan J., Lazagabaster I.A., Campisano C.J., Bibi F., Bobe R., Boisserie J.R., Frost S.R., Getachew T., Gilbert C.C., Lewis M.E. (2022). Early Pleistocene Large Mammals from Maka’amitalu, Hadar, Lower Awash Valley, Ethiopia. PeerJ.

[38] Domínguez-Rodrigo M., Pickering T.R., Semaw S., Rogers M.J. (2005). Cutmarked Bones from Pliocene Archaeological Sites at Gona, Afar, Ethiopia: Implications for the Function of the World’s Oldest Stone Tools. J. Hum. Evol..

[39] Ferraro J.V., Plummer T.W., Pobiner B.L., Oliver J.S., Bishop L.C., Braun D.R., Ditchfield P.W., Seaman J.W., Binetti K.M., Seaman J.W. (2013). Earliest Archaeological Evidence of Persistent Hominin Carnivory. PLoS ONE.

[40] Curran S.C., Drăgușin V., Pobiner B., Pante M., Hellstrom J., Woodhead J., Croitor R., Doboș A., Gogol S.E., Ersek V. (2025). Hominin Presence in Eurasia by at Least 1.95 Million Years Ago. Nat. Commun..

[41] Gorbunova N.A. (2024). Assessing the Role of Meat Consumption in Human Evolutionary Changes. A Review. Theory Pract. Meat Process..

[42] Antón S.C., Taboada H.G., Middleton E.R., Rainwater C.W., Taylor A.B., Turner T.R., Turnquist J.E., Weinstein K.J., Williams S.A. (2016). Morphological Variation in Homo Erectus and the Origins of Developmental Plasticity. Philos. Trans. R. Soc. B Biol. Sci..

[43] Puech P.F., Albertini H., Serratrice C. (1983). Tooth Microwear and Dietary Patterns in Early Hominids from Laetoli, Hadar and Olduvai. J. Hum. Evol..

[44] Zink K.D., Lieberman D.E. (2016). Impact of Meat and Lower Palaeolithic Food Processing Techniques on Chewing in Humans. Nature.

[45] Day L., Cakebread J.A., Loveday S.M. (2022). Food Proteins from Animals and Plants: Differences in the Nutritional and Functional Properties. Trends Food Sci. Technol..

[46] Harvati K., Reyes-Centeno H. (2022). Evolution of Homo in the Middle and Late Pleistocene. J. Hum. Evol..

[47] Richards M.P., Trinkaus E. (2009). Out of Africa: Modern Human Origins Special Feature: Isotopic Evidence for the Diets of European Neanderthals and Early Modern Humans. Proc. Natl. Acad. Sci. USA.

[48] Jaouen K., Beasley M., Schoeninger M., Hublin J.J., Richards M.P. (2016). Zinc Isotope Ratios of Bones and Teeth as New Dietary Indicators: Results from a Modern Food Web (Koobi Fora, Kenya). Sci. Rep..

[49] Hardy K., Buckley S., Collins M.J., Estalrrich A., Brothwell D., Copeland L., García-Tabernero A., García-Vargas S., De La Rasilla M., Lalueza-Fox C. (2012). Neanderthal Medics? Evidence for Food, Cooking, and Medicinal Plants Entrapped in Dental Calculus. Naturwissenschaften.

[50] Rampelli S., Turroni S., Mallol C., Hernandez C., Galván B., Sistiaga A., Biagi E., Astolfi A., Brigidi P., Benazzi S. (2021). Components of a Neanderthal Gut Microbiome Recovered from Fecal Sediments from El Salt. Commun. Biol..

[51] Shipley G.P., Kindscher K. (2016). Evidence for the Paleoethnobotany of the Neanderthal: A Review of the Literature. Scientifica.

[52] Palma-Morales M., Mateos A., Rodríguez J., Casuso R.A., Huertas J.R. (2022). Food Made Us Human: Recent Genetic Variability and Its Relevance to the Current Distribution of Macronutrients. Nutrition.

[53] Barsky D., Vergès J.M., Titton S., Guardiola M., Sala R., Moyano I.T. (2018). The Emergence and Significance of Heavy-Duty Scrapers in Ancient Stone Toolkits. Comptes Rendus Palevol.

[54] de la Torre I., Doyon L., Benito-Calvo A., Mora R., Mwakyoma I., Njau J.K., Peters R.F., Theodoropoulou A., d’Errico F. (2025). Systematic Bone Tool Production at 1.5 Million Years Ago. Nature.

[55] Braun D.R., Pante M., Archer W. (2016). Cut Marks on Bone Surfaces: Influences on Variation in the Form of Traces of Ancient Behaviour. Interface Focus.

[56] Muller A., Shipton C., Clarkson C. (2022). Stone Toolmaking Difficulty and the Evolution of Hominin Technological Skills. Sci. Rep..

[57] Conard N.J., Serangeli J., Böhner U., Starkovich B.M., Miller C.E., Urban B., Van Kolfschoten T. (2015). Excavations at Schöningen and Paradigm Shifts in Human Evolution. J. Hum. Evol..

[58] Zink K.D., Lieberman D.E., Lucas P.W. (2014). Food Material Properties and Early Hominin Processing Techniques. J. Hum. Evol..

[59] Karkanas P., Shahack-Gross R., Ayalon A., Bar-Matthews M., Barkai R., Frumkin A., Gopher A., Stiner M.C. (2007). Evidence for Habitual Use of Fire at the End of the Lower Paleolithic: Site-Formation Processes at Qesem Cave, Israel. J. Hum. Evol..

[60] Shipman P., Rose J. (1983). Early Hominid Hunting, Butchering, and Carcass-Processing Behaviors: Approaches to the Fossil Record. J. Anthropol. Archaeol..

[61] Blasco R., Rosell J., Arilla M., Margalida A., Villalba D., Gopher A., Barkai R. (2019). Bone Marrow Storage and Delayed Consumption at Middle Pleistocene Qesem Cave, Israel (420 to 200 Ka). Sci. Adv..

[62] Pante M., de La T.A., d’Errico F., Njau J., Blumenschine R. (2020). Bone Tools from Beds II–IV, Olduvai Gorge, Tanzania, and Implications for the Origins and Evolution of Bone Technology. J. Hum. Evol..

[63] Ollé A., Mosquera M., Rodríguez X.P., de Lombera-Hermida A., García-Antón M.D., García-Medrano P., Peña L., Menéndez L., Navazo M., Terradillos M. (2013). The Early and Middle Pleistocene Technological Record from Sierra de Atapuerca (Burgos, Spain). Quat. Int..

[64] Solodenko N., Zupancich A., Cesaro S.N., Marder O., Lemorini C., Barkai R. (2015). Fat Residue Use-Wear Found on Acheulian Biface Scraper Associated with Butchered Elephant Remains at the Site of Revadim, Israel. PLoS ONE.

[65] Bhargavi A., Ajay S., Rohit B., Vishal A., Minkle G. (2013). Comparative Tooth Anatomy—A Review. Int. J. Dent. Sci. Res..

[66] Grenot C.J. (1992). Ecophysiological Characteristics of Large Herbivorous Mammals in Arid Africa and the Middle East. J. Arid Environ..

[67] Linnell J.D.C., Cretois B., Nilsen E.B., Rolandsen C.M., Solberg E.J., Veiberg V., Kaczensky P., Van Moorter B., Panzacchi M., Rauset G.R. (2020). The Challenges and Opportunities of Coexisting with Wild Ungulates in the Human-Dominated Landscapes of Europe’s Anthropocene. Biol. Conserv..

[68] Kim G., Oh J., Cho M. (2022). Differences between Vegetarians and Omnivores in Food Choice Motivation and Dietarian Identity. Foods.

[69] ZAWAWI R.N., ALMOSA N.A. (2023). Assessment of Enamel Surface Roughness and Hardness with Metal and Ceramic Orthodontic Brackets Using Different Etching and Adhesive Systems: An in Vitro Study. Saudi Dent. J..

[70] Daubert D.M., Kelley J.L., Udod Y.G., Habor C., Kleist C.G., Furman I.K., Tikonov I.N., Swanson W.J., Roberts F.A. (2016). Human Enamel Thickness and ENAM Polymorphism. Int. J. Oral Sci..

[71] Ricketts R.M. (1955). Facial and Denture Changes during Orthodontic Treatment as Analyzed from the Temporomandibular Joint. Am. J. Orthod..

[72] Koc D., Dogan A., Bek B. (2010). Bite Force and Influential Factors on Bite Force Measurements: A Literature Review. Eur. J. Dent..

[73] Evans A.R., Daly E.S., Catlett K.K., Paul K.S., King S.J., Skinner M.M., Nesse H.P., Hublin J.J., Townsend G.C., Schwartz G.T. (2016). A Simple Rule Governs the Evolution and Development of Hominin Tooth Size. Nature.

[74] Ungar P.S., Grine F.E., Teaford M.F. (2008). Dental Microwear and Diet of the Plio-Pleistocene Hominin Paranthropus Boisei. PLoS ONE.

[75] Zollikofer C.P.E., Beyrand V., Lordkipanidze D., Tafforeau P., Ponce de León M.S. (2024). Dental Evidence for Extended Growth in Early Homo from Dmanisi. Nature.

[76] Domínguez-Solera S.D., Martín-Lerma I., Moreno D., Pérez-Garrido C. (2022). Lower Paleolithic Butchery Knives and Carpentry Tools: MODE 1 Industry of “El Pino” (Campos Del Paraíso, Cuenca, Spain). J. Archaeol. Sci. Rep..

[77] Prat S. (2022). Emergence of the Genus Homo: From Concept to Taxonomy. Anthropologie.

[78] Thackeray J.F., Maureille B., Vandermeersch B., Braga J., Chaix R. (2005). Morphometric Comparisons between Neanderthals and ‘Anatomically Modern’ Homo Sapiens from Europe and the Near East. Ann. Transvaal Musuem.

[79] Kondo M., Ikenaka Y., Nakayama S.M.M., Kawai Y.K., Mizukawa H., Mitani Y., Nomyama K., Tanabe S., Ishizuka M. (2023). Sulfotransferases (SULTs), Enzymatic and Genetic Variation in Carnivora: Limited Sulfation Capacity in Pinnipeds. Comp. Biochem. Physiol. Part—C Toxicol. Pharmacol..

[80] Luca F., Perry G.H., Di Rienzo A. (2010). Evolutionary Adaptations to Dietary Changes. Annu. Rev. Nutr..

[81] James W.P.T., Johnson R.J., Speakman J.R., Wallace D.C., Frühbeck G., Iversen P.O., Stover P.J. (2019). Nutrition and Its Role in Human Evolution. J. Intern. Med..

[82] Rees J.S., Castellano S., Andrés A.M. (2020). The Genomics of Human Local Adaptation. Trends Genet..

[83] Esteves F., Rueff J., Kranendonk M. (2021). The Central Role of Cytochrome P450 in Xenobiotic Metabolism—A Brief Review on a Fascinating Enzyme Family. J. Xenobiotics.

[84] He W., Connolly E.D., Wu G. (2024). Characteristics of the Digestive Tract of Dogs and Cats.

[85] Gibson L.A., Hume I.D. (2000). Digestive Performance and Digesta Passage in the Omnivorous Greater Bilby, Macrotis Lagotis (Marsupialia: Peramelidae). J. Comp. Physiol. B.

[86] Pujahari A.K. (2016). Tiger Food for Short Bowel: Two Cases. J. Clin. Diagn. Res..

[87] Helander H.F., Fändriks L. (2014). Surface Area of the Digestive Tract—Revisited. Scand. J. Gastroenterol..

[88] Neves M.P., Amorim J.P.d.A., Delariva R.L., Kratina P., Fialho C.B. (2024). Linking Anatomical and Histological Traits of the Digestive Tract to Resource Consumption and Assimilation of Omnivorous Tetra Fishes. Ecol. Evol..

[89] Liu Y., Shu Y., Huang Y., Tan J., Wang F., Tang L., Fang T., Yuan S., Wang L. (2024). Microbial Biogeography along the Gastrointestinal Tract of a Wild Chinese Muntjac (Muntiacus Reevesi). Microorganisms.

[90] Harter T.S., Verreth J.A.J., Heinsbroek L.T.N., Schrama J.W. (2013). Isoenergetic Replacement of Fat by Starch in Diets for African Catfish (Clarias Gariepinus): Effect on Water Fluxes in the Gastro Intestinal Tract. PLoS ONE.

[91] McKenney E.A., Hale A.R., Anderson J., Larsen R., Grant C., Dunn R.R. (2023). Hidden Diversity: Comparative Functional Morphology of Humans and Other Species. PeerJ.

[92] Leroy F., Smith N.W., Adesogan A.T., Beal T., Iannotti L., Moughan P.J., Mann N. (2023). The Role of Meat in the Human Diet: Evolutionary Aspects and Nutritional Value. Anim. Front..

[93] Xiao J., Metzler-Zebeli B.U., Zebeli Q. (2015). Gut Function-Enhancing Properties and Metabolic Effects of Dietary Indigestible Sugars in Rodents and Rabbits. Nutrients.

[94] Havenaar R. (2011). Intestinal Health Functions of Colonic Microbial Metabolites: A Review. Benef. Microbes.

[95] Brown K., Abbott D.W., Uwiera R.R.E., Inglis G.D. (2018). Removal of the Cecum Affects Intestinal Fermentation, Enteric Bacterial Community Structure, and Acute Colitis in Mice. Gut Microbes.

[96] Sulistyowati E., Handayani D., Soeharto S., Rudijanto A. (2022). A High-Fat and High-Fructose Diet Lowers the Cecal Digesta’s Weight and Short-Chain Fatty Acid Level of a Sprague–Dawley Rat Model. Turk. J. Med. Sci..

[97] Fu B., Zhao X., Khan M., Jiang Y., Li W., Mushtaq M., Danzeng B., Ni X., Azeem Z., Shao Q. (2025). Cecum Microbiota Composition, Fermentation Characteristics, and Immunometabolic Biomarkers of Yunshang Black Goat Fed Varying Dietary Energy and Protein Levels. Front. Microbiol.

[98] Zaborin A., Penalver Bernabe B., Keskey R., Sangwan N., Hyoju S., Gottel N., Gilbert J.A., Zaborina O., Alverdy J.C. (2020). Spatial Compartmentalization of the Microbiome between the Lumen and Crypts Is Lost in the Murine Cecum Following the Process of Surgery, Including Overnight Fasting and Exposure to Antibiotics. Msystems.

[99] Kim C.H. (2018). Microbiota or Short-Chain Fatty Acids: Which Regulates Diabetes?. Cell. Mol. Immunol..

[100] Bhat M.A., Mishra A.K., Tantray J.A., Alatawi H.A., Saeed M., Rahman S., Jan A.T. (2022). Gut Microbiota and Cardiovascular System: An Intricate Balance of Health and the Diseased State. Life.

[101] Mirzaei R., Afaghi A., Babakhani S., Sohrabi M.R., Hosseini-Fard S.R., Babolhavaeji K., Khani Ali Akbari S., Yousefimashouf R., Karampoor S. (2021). Role of Microbiota-Derived Short-Chain Fatty Acids in Cancer Development and Prevention. Biomed. Pharmacother..

[102] Mariño E., Richards J.L., McLeod K.H., Stanley D., Yap Y.A., Knight J., McKenzie C., Kranich J., Oliveira A.C., Rossello F.J. (2017). Gut Microbial Metabolites Limit the Frequency of Autoimmune T Cells and Protect against Type 1 Diabetes. Nat. Immunol..

[103] Urlacher S.S., Kim E.Y., Luan T., Young L.J., Adjetey B. (2022). Minimally Invasive Biomarkers in Human and Non-Human Primate Evolutionary Biology: Tools for Understanding Variation and Adaptation. Am. J. Hum. Biol..

[104] Amato K.R. (2016). Incorporating the Gut Microbiota into Models of Human and Non-Human Primate Ecology and Evolution. Am. J. Phys. Anthropol..

[105] MacKie A. (2023). The Role of Food Structure in Gastric Emptying Rate, Absorption and Metabolism. Proc. Nutr. Soc..

[106] Goyal R.K., Guo Y., Mashimo H. (2019). Advances in the Physiology of Gastric Emptying. Neurogastroenterol. Motil..

[107] Kuhar S., Lee J.H., Seo J.H., Pasricha P.J., Mittal R. (2022). Effect of Stomach Motility on Food Hydrolysis and Gastric Emptying: Insight from Computational Models. Phys. Fluids.

[108] Fernandes S.Q., Kothare M.V., Mahmoudi B. (2024). A Novel Compartmental Approach for Modeling Stomach Motility and Gastric Emptying. Comput. Biol. Med..

[109] Ebara R., Ishida S., Miyagawa T., Imai Y. (2023). Effects of Peristaltic Amplitude and Frequency on Gastric Emptying and Mixing: A Simulation Study. J. R. Soc. Interface.

[110] Miyagawa T., Imai Y., Ishida S., Ishikawa T. (2016). Relationship between Gastric Motility and Liquid Mixing in the Stomach. Am. J. Physiol. Gastrointest Liver Physiol..

[111] Goyal R.K., Cristofaro V., Sullivan M.P. (2019). Rapid Gastric Emptying in Diabetes Mellitus: Pathophysiology and Clinical Importance. J. Diabetes Its Complicat..

[112] Mori H., Verbeure W., Schol J., Carbone F., Tack J. (2022). Gastrointestinal Hormones and Regulation of Gastric Emptying. Curr. Opin. Endocrinol. Diabetes Obes..

[113] Wei L., Singh R., Ha S.E., Martin A.M., Jones L.A., Jin B., Jorgensen B.G., Zogg H., Chervo T., Gottfried-Blackmore A. (2021). Serotonin Deficiency Is Associated with Delayed Gastric Emptying. Gastroenterology.

[114] Barr B., Levitt D.E., Gollahon L. (2025). Red Meat Amino Acids for Beginners: A Narrative Review. Nutrients.

[115] Wang Z., Xu W., Liu D., Li X., Liu S., Wu X., Wang H. (2025). Impact of Food Physical Properties on Oral Drug Absorption: A Comprehensive Review. Drug Des. Dev. Ther..

[116] Tian L., Huang C., Fu W., Gao L., Mi N., Bai M., Ma H., Zhang C., Lu Y., Zhao J. (2023). Proton Pump Inhibitors May Enhance the Risk of Digestive Diseases by Regulating Intestinal Microbiota. Front. Pharmacol..

[117] Freedberg D.E., Lebwohl B., Abrams J.A. (2014). The Impact of Proton Pump Inhibitors on the Human Gastrointestinal Microbiome. Clin. Lab. Med..

[118] Adetunji C.O., Olaniyan O.T., Varma A. (2022). Enzymes Involved with Digestion of Animal Nutrition: Role and Their Biotechnological Application.

[119] Corsato Alvarenga I., Aldrich C.G., Shi Y.C. (2021). Factors Affecting Digestibility of Starches and Their Implications on Adult Dog Health. Anim. Feed. Sci. Technol..

[120] Janiak M.C. (2016). Digestive Enzymes of Human and Nonhuman Primates. Evol. Anthropol..

[121] Dominy N.J., Vogel E.R., Yeakel J.D., Constantino P., Lucas P.W. (2008). Mechanical Properties of Plant Underground Storage Organs and Implications for Dietary Models of Early Hominins. Evol. Biol..

[122] Zhang J. (2006). Parallel Adaptive Origins of Digestive RNases in Asian and African Leaf Monkeys. Nat. Genet..

[123] Perry G.H., Dominy N.J., Claw K.G., Lee A.S., Fiegler H., Redon R., Werner J., Villanea F.A., Mountain J.L., Misra R. (2007). Diet and the Evolution of Human Amylase Gene Copy Number Variation. Nat. Genet..

[124] Heda R., Toro F., Tombazzi C.R. (2023). Physiology, Pepsin.

[125] Robertson D.S. (2005). The Chemical Reactions in the Human Stomach and the Relationship to Metabolic Disorders. Med. Hypotheses.

[126] Yang M., Yang Z., Everett D.W., Gilbert E.P., Singh H., Ye A. (2025). Digestion of Food Proteins: The Role of Pepsin. Crit. Rev. Food Sci. Nutr..

[127] Whitcomb D.C., Lowe M.E. (2007). Human Pancreatic Digestive Enzymes. Dig. Dis. Sci..

[128] Karasov W.H., Douglas A.E. (2013). Comparative Digestive Physiology. Compr. Physiol..

[129] Goldberg D.M. (1976). Functional, Chemical, and Clinical Aspects of Proteolytic Enzymes in the Alimentary Canal. Clin. Biochem..

[130] Bolognini D., Halgren A., Lou R.N., Raveane A., Rocha J.L., Guarracino A., Soranzo N., Chin C.-S., Garrison E., Sudmant P.H. (2024). Recurrent Evolution and Selection Shape Structural Diversity at the Amylase Locus. Nature.

[131] Yang Q., Chang S., Zhang X., Luo F., Li W., Ren J. (2024). The Fate of Dietary Polysaccharides in the Digestive Tract. Trends Food Sci. Technol..

[132] Fu J., Zheng Y., Gao Y., Xu W. (2022). Dietary Fiber Intake and Gut Microbiota in Human Health. Microorganisms.

[133] Holland C., Ryden P., Edwards C.H., Grundy M.M.L. (2020). Plant Cell Walls: Impact on Nutrient Bioaccessibility and Digestibility. Foods.

[134] German D.P., Horn M.H., Gawlicka A. (2004). Digestive Enzyme Activities in Herbivorous and Carnivorous Prickleback Fishes (Teleostei: Stichaeidae): Ontogenetic, Dietary, and Phylogenetic Effects. Physiol. Biochem. Zool..

[135] Castro-Ruiz D., Andree K.B., Blondeau-Bidet E., Fernández-Méndez C., García-Dávila C., Gisbert E., Darias M.J. (2021). Isolation, Identification, and Gene Expression Analysis of the Main Digestive Enzymes during Ontogeny of the Neotropical Catfish Pseudoplatystoma Punctifer (Castelnau, 1855). Aquaculture.

[136] Chen Y.H., Zhao H. (2019). Evolution of Digestive Enzymes and Dietary Diversification in Birds. PeerJ.

[137] Boehlke C., Zierau O., Hannig C. (2015). Salivary Amylase—The Enzyme of Unspecialized Euryphagous Animals. Arch. Oral Biol..

[138] Verbrugghe A., Hesta M. (2017). Cats and Carbohydrates: The Carnivore Fantasy?. Vet. Sci..

[139] Llobat L., Soriano P., Bordignon F., de Evan T., Larsen T., Marín-García P.J. (2024). Dietary Type (Carnivore, Herbivore and Omnivore) and Animal Species Modulate the Nutritional Metabolome of Terrestrial Species. Comp. Biochem. Physiol. B Biochem. Mol. Biol..

[140] Dwyer G.K., Stoffels R.J., Silvester E., Rees G.N. (2023). Two Wild Carnivores Selectively Forage for Prey but Not Amino Acids. Sci. Rep..

[141] Kim S., Cho Y.S., Kim H.M., Chung O., Kim H., Jho S., Seomun H., Kim J., Bang W.Y., Kim C. (2016). Comparison of Carnivore, Omnivore, and Herbivore Mammalian Genomes with a New Leopard Assembly. Genome Biol..

[142] Lonnie M., Hooker E., Brunstrom J.M., Corfe B.M., Green M.A., Watson A.W., Williams E.A., Stevenson E.J., Penson S., Johnstone A.M. (2018). Protein for Life: Review of Optimal Protein Intake, Sustainable Dietary Sources and the Effect on Appetite in Ageing Adults. Nutrients.

[143] Christiansen P., Wroe S. (2007). Bite Forces and Evolutionary Adaptations to Feeding Ecology in Carnivores. Ecology.

[144] Zhu G., Fang Q., Zhu F., Huang D., Yang C. (2021). Structure and Function of Pancreatic Lipase-Related Protein 2 and Its Relationship with Pathological States. Front. Genet..

[145] De Cuyper A., Meloro C., Abraham A.J., Müller D.W.H., Codron D., Janssens G.P.J., Clauss M. (2020). The Uneven Weight Distribution between Predators and Prey: Comparing Gut Fill between Terrestrial Herbivores and Carnivores. Comp. Biochem. Physiol. A Mol. Integr. Physiol..

[146] Hoffmann A., Sgró C. (2011). Climate Change and Evolutionary Adaptation. Nature.

[147] Capuano E. (2017). The Behavior of Dietary Fiber in the Gastrointestinal Tract Determines Its Physiological Effect. Crit. Rev. Food Sci. Nutr..

[148] Chen Y., Michalak M., Agellon L.B. (2018). Importance of Nutrients and Nutrient Metabolism on Human Health. Yale J. Biol. Med..

[149] Nutrient Metabolism, Human|Learn Science at Scitable. https://www.nature.com/scitable/topicpage/dynamic-adaptation-of-nutrient-utilization-in-humans-14232807/.

[150] Smith R.L., Soeters M.R., Wüst R.C.I., Houtkooper R.H. (2018). Metabolic Flexibility as an Adaptation to Energy Resources and Requirements in Health and Disease. Endocr. Rev..

[151] Clemente-Suárez V.J., Mielgo-Ayuso J., Martín-Rodríguez A., Ramos-Campo D.J., Redondo-Flórez L., Tornero-Aguilera J.F. (2022). The Burden of Carbohydrates in Health and Disease. Nutrients.

[152] Alberts B., Johnson A., Lewis J., Raff M., Roberts K., Walter P. (2002). How Cells Obtain Energy Food.

[153] Nelson D.L., Cox M.M. (2001). Lehninger Biochemie. https://cir.nii.ac.jp/crid/1361137044511668736#citations_container.

[154] Corsetti G., Pasini E., Scarabelli T.M., Romano C., Singh A., Scarabelli C.C., Dioguardi F.S. (2024). Importance of Energy, Dietary Protein Sources, and Amino Acid Composition in the Regulation of Metabolism: An Indissoluble Dynamic Combination for Life. Nutrients.

[155] Princiotta M.F., Finzi D., Qian S.B., Gibbs J., Schuchmann S., Buttgereit F., Bennink J.R., Yewdell J.W. (2003). Quantitating Protein Synthesis, Degradation, and Endogenous Antigen Processing. Immunity.

[156] Hardie D.G. (2011). AMP-Activated Protein Kinase—An Energy Sensor That Regulates All Aspects of Cell Function. Genes Dev..

[157] Hardie D.G., Ross F.A., Hawley S.A. (2012). AMPK—A Nutrient and Energy Sensor That Maintains Energy Homeostasis. Nat. Rev. Mol. Cell Biol..

[158] Rehman S.U., Ali R., Zhang H., Zafar M.H., Wang M. (2023). Research Progress in the Role and Mechanism of Leucine in Regulating Animal Growth and Development. Front. Physiol..

[159] Dreyer H.C., Drummond M.J., Pennings B., Fujita S., Glynn E.L., Chinkes D.L., Dhanani S., Volpi E., Rasmussen B.B. (2007). Leucine-Enriched Essential Amino Acid and Carbohydrate Ingestion Following Resistance Exercise Enhances MTOR Signaling and Protein Synthesis in Human Muscle. Am. J. Physiol. Endocrinol. Metab..

[160] Chandel N.S. (2021). Lipid Metabolism. Cold Spring Harb. Perspect. Biol..

[161] Grabner G.F., Xie H., Schweiger M., Zechner R. (2021). Lipolysis: Cellular Mechanisms for Lipid Mobilization from Fat Stores. Nat. Metab..

[162] Edwards M., Mohiuddin S.S. (2023). Biochemistry, Lipolysis.

[163] Panov A.V., Mayorov V.I., Dikalov S.I. (2024). Role of Fatty Acids β-Oxidation in the Metabolic Interactions Between Organs. Int. J. Mol. Sci..

[164] Puchalska P., Crawford P.A. (2021). Metabolic and Signaling Roles of Ketone Bodies in Health and Disease. Annu. Rev. Nutr..

[165] Mergenthaler P., Lindauer U., Dienel G.A., Meisel A. (2013). Sugar for the Brain: The Role of Glucose in Physiological and Pathological Brain Function. Trends Neurosci..

[166] Li X., Zhu X., Han D., Yang Y., Jin J., Xie S. (2016). Carbohydrate Utilization by Herbivorous and Omnivorous Freshwater Fish Species: A Comparative Study on Gibel Carp (Carassius Auratus Gibelio. Var. CAS III) Grass Carp. (Ctenopharyngodon Idellus). Aquac. Res..

[167] Hawlena D., Schmitz O.J. (2010). Herbivore Physiological Response to Predation Risk and Implications for Ecosystem Nutrient Dynamics. Proc. Natl. Acad. Sci. USA.

[168] Clemente-Suárez V.J., Redondo-Flórez L., Martín-Rodríguez A., Curiel-Regueros A., Rubio-Zarapuz A., Tornero-Aguilera J.F. (2025). Impact of Vegan and Vegetarian Diets on Neurological Health: A Critical Review. Nutrients.

[169] Cellulose in Digestion—Animals, Ruminants, & Herbivores—GeeksforGeeks. https://www.geeksforgeeks.org/cellulose-in-digestion-herbivores-termites-ruminants/.

[170] Sensoy I. (2021). A Review on the Food Digestion in the Digestive Tract and the Used in Vitro Models. Curr. Res. Food Sci..

[171] Foley P.J. (2021). Effect of Low Carbohydrate Diets on Insulin Resistance and the Metabolic Syndrome. Curr. Opin. Endocrinol. Diabetes Obes..

[172] Ashtary-Larky D., Bagheri R., Bavi H., Baker J.S., Moro T., Mancin L., Paoli A. (2021). Ketogenic Diets, Physical Activity and Body Composition: A Review. Br. J. Nutr..

[173] Galali Y., Zebari S.M.S., Aj Jabbar A., Hashm Balaky H., Sadee B.A., Hassanzadeh H. (2024). The Impact of Ketogenic Diet on Some Metabolic and Non-Metabolic Diseases: Evidence from Human and Animal Model Experiments. Food Sci. Nutr..

[174] Barber T.M., Hanson P., Kabisch S., Pfeiffer A.F.H., Weickert M.O. (2021). The Low-Carbohydrate Diet: Short-Term Metabolic Efficacy Versus Longer-Term Limitations. Nutrients.

[175] Tzenios N., Lewis E.D., Crowley D.C., Chahine M., Evans M. (2022). Examining the Efficacy of a Very-Low-Carbohydrate Ketogenic Diet on Cardiovascular Health in Adults with Mildly Elevated Low-Density Lipoprotein Cholesterol in an Open-Label Pilot Study. Metab. Syndr. Relat. Disord..

[176] Wachsmuth N.B., Aberer F., Haupt S., Schierbauer J.R., Zimmer R.T., Eckstein M.L., Zunner B., Schmidt W., Niedrist T., Sourij H. (2022). The Impact of a High-Carbohydrate/Low Fat vs. Low-Carbohydrate Diet on Performance and Body Composition in Physically Active Adults: A Cross-Over Controlled Trial. Nutrients.

[177] Santamarina A.B., Mennitti L.V., de Souza E.A., Mesquita L.M.d.S., Noronha I.H., Vasconcelos J.R.C., Prado C.M., Pisani L.P. (2023). A Low-Carbohydrate Diet with Different Fatty Acids’ Sources in the Treatment of Obesity: Impact on Insulin Resistance and Adipogenesis. Clin. Nutr..

[178] Mozaffarian D. (2016). Dietary and Policy Priorities for Cardiovascular Disease, Diabetes, and Obesity: A Comprehensive Review. Circulation.

[179] Oddy W.H., Herbison C.E., Jacoby P., Ambrosini G.L., O’Sullivan T.A., Ayonrinde O.T., Olynyk J.K., Black L.J., Beilin L.J., Mori T.A. (2013). The Western Dietary Pattern Is Prospectively Associated with Nonalcoholic Fatty Liver Disease in Adolescence. Am. J. Gastroenterol..

[180] Belinova L., Kahleova H., Malinska H., Topolcan O., Vrzalova J., Oliyarnyk O., Kazdova L., Hill M., Pelikanova T. (2014). Differential Acute Postprandial Effects of Processed Meat and Isocaloric Vegan Meals on the Gastrointestinal Hormone Response in Subjects Suffering from Type 2 Diabetes and Healthy Controls: A Randomized Crossover Study. PLoS ONE.

[181] Cocate P.G., Natali A.J., Oliveira A.D., Alfenas R.D.C.G., Peluzio M.D.C.G., Longo G.Z., Santos E.C.D., Buthers J.M., De Oliveira L.L., Hermsdorff H.H.M. (2015). Red but Not White Meat Consumption Is Associated with Metabolic Syndrome, Insulin Resistance and Lipid Peroxidation in Brazilian Middle-Aged Men. Eur. J. Prev. Cardiol..

[182] Fretts A.M., Follis J.L., Nettleton J.A., Lemaitre R.N., Ngwa J.S., Wojczynski M.K., Kalafati I.P., Varga T.V., Frazier-Wood A.C., Houston D.K. (2015). Consumption of Meat Is Associated with Higher Fasting Glucose and Insulin Concentrations Regardless of Glucose and Insulin Genetic Risk Scores: A Meta-Analysis of 50,345 Caucasians. Am. J. Clin. Nutr..

[183] Wang Z., Klipfell E., Bennett B.J., Koeth R., Levison B.S., Dugar B., Feldstein A.E., Britt E.B., Fu X., Chung Y.M. (2011). Gut Flora Metabolism of Phosphatidylcholine Promotes Cardiovascular Disease. Nature.

[184] Papandreou C., Moré M., Bellamine A. (2020). Trimethylamine N-Oxide in Relation to Cardiometabolic Health—Cause or Effect?. Nutrients.

[185] Wu W.-K., Lo Y.-L., Chiu J.-Y., Hsu C.-L., Lo I.-H., Panyod S., Liao Y.-C., Chiu T.H.T., Yang Y.-T., Kuo H.-C. (2025). Gut Microbes with the *Gbu* Genes Determine TMAO Production from L-Carnitine Intake and Serve as a Biomarker for Precision Nutrition. Gut Microbes.

[186] Ahmad M.I., Umair Ijaz M., Hussain M., Ali Khan I., Mehmood N., Siddiqi S.M., Liu C., Zhao D., Xu X., Zhou G. (2020). High Fat Diet Incorporated with Meat Proteins Changes Biomarkers of Lipid Metabolism, Antioxidant Activities, and the Serum Metabolomic Profile in Glrx1-/- Mice. Food Funct..

[187] Arage G., Dekkers K.F., Rašo L.M., Hammar U., Ericson U., Larsson S.C., Engel H., Baldanzi G., Pertiwi K., Sayols-Baixeras S. (2025). Plasma Metabolite Profiles of Meat Intake and Their Association with Cardiovascular Disease Risk: A Population-Based Study in Swedish Cohorts. Metabolism.

[188] Lanuza F., Meroño T., Zamora-Ros R., Bondonno N.P., Rostgaard-Hansen A.L., Sánchez-Pla A., Miro B., Carmona-Pontaque F., Riccardi G., Tjønneland A. (2023). Plasma Metabolomic Profiles of Plant-Based Dietary Indices Reveal Potential Pathways for Metabolic Syndrome Associations. Atherosclerosis.

[189] Chen L., Dai J., Fei Z., Liu X., Zhu Y., Rahman M.L., Lu R., Mitro S.D., Yang J., Hinkle S.N. (2023). Metabolomic Biomarkers of the Mediterranean Diet in Pregnant Individuals: A Prospective Study. Clin. Nutr..

[190] Huang Z.B., Hu Z., Lu C.X., Luo S.D., Chen Y., Zhou Z.P., Hu J.J., Zhang F.L., Deng F., Liu K.X. (2022). Gut Microbiota-Derived Indole 3-Propionic Acid Partially Activates Aryl Hydrocarbon Receptor to Promote Macrophage Phagocytosis and Attenuate Septic Injury. Front. Cell. Infect. Microbiol..

[191] Qi Q., Li J., Yu B., Moon J.Y., Chai J.C., Merino J., Hu J., Ruiz-Canela M., Rebholz C., Wang Z. (2022). Host and Gut Microbial Tryptophan Metabolism and Type 2 Diabetes: An Integrative Analysis of Host Genetics, Diet, Gut Microbiome and Circulating Metabolites in Cohort Evidence. Gut.

[192] Baldi S., Tristán Asensi M., Pallecchi M., Sofi F., Bartolucci G., Amedei A. (2023). Interplay between Lignans and Gut Microbiota: Nutritional, Functional and Methodological Aspects. Molecules.

[193] Liu G., Li J., Li Y., Hu Y., Franke A.A., Liang L., Hu F.B., Chan A.T., Mukamal K.J., Rimm E.B. (2021). Gut Microbiota-Derived Metabolites and Risk of Coronary Artery Disease: A Prospective Study among US Men and Women. Am. J. Clin. Nutr..

[194] Syauqy A., Hsu C.-Y., Lee H.-A., Rau H.-H., Chao J.C.-J. (2020). Association between Dietary Patterns and Kidney Function Parameters in Adults with Metabolic Syndrome: A Cross-Sectional Study. Nutrients.

[195] Mahajan H., Mallinson P.A.C., Lieber J., Bhogadi S., Banjara S.K., Reddy V.S., Reddy G.B., Kulkarni B., Kinra S. (2024). The Association of Total Meat Intake with Cardio-Metabolic Disease Risk Factors and Measures of Sub-Clinical Atherosclerosis in an Urbanising Community of Southern India: A Cross-Sectional Analysis for the APCAPS Cohort. Nutrients.

[196] Sholl J., Mailing L.J., Wood T.R. (2021). Reframing Nutritional Microbiota Evidence to Reflect an Inherent Metabolic Flexibility of the Human Gut: A Narrative Review Focusing on High-Fat Diets. mBio.

[197] Mostafavi Abdolmaleky H., Zhou J.R. (2024). Gut Microbiota Dysbiosis, Oxidative Stress, Inflammation, and Epigenetic Alterations in Metabolic Diseases. Antioxidants.

[198] Sheflin A.M., Melby C.L., Carbonero F., Weir T.L. (2016). Linking Dietary Patterns with Gut Microbial Composition and Function. Gut Microbes.

[199] Moeller A.H., Sanders J.G. (2020). Roles of the Gut Microbiota in the Adaptive Evolution of Mammalian Species. Philos. Trans. R. Soc. B Biol. Sci..

[200] de Jonge N., Carlsen B., Christensen M.H., Pertoldi C., Nielsen J.L. (2022). The Gut Microbiome of 54 Mammalian Species. Front. Microbiol..

[201] Dearing M.D., Kohl K.D. (2017). Beyond Fermentation: Other Important Services Provided to Endothermic Herbivores by Their Gut Microbiota. Integr. Comp. Biol..

[202] Zoelzer F., Burger A.L., Dierkes P.W. (2021). Unraveling Differences in Fecal Microbiota Stability in Mammals: From High Variable Carnivores and Consistently Stable Herbivores. Anim. Microbiome.

[203] Milani C., Alessandri G., Mancabelli L., Mangifesta M., Lugli G.A., Viappiani A., Longhi G., Anzalone R., Duranti S., Turroni F. (2020). Multi-omics Approaches to Decipher the Impact of Diet and Host Physiology on the Mammalian Gut Microbiome. Appl. Environ. Microbiol..

[204] Schwab C., Gänzle M. (2011). Comparative Analysis of Fecal Microbiota and Intestinal Microbial Metabolic Activity in Captive Polar Bears. Can. J. Microbiol..

[205] Todd E.C.D. (2014). Foodborne Diseases: Overview of Biological Hazards and Foodborne Diseases. Encycl. Food Saf..

[206] Clostridium Perfringens—GOV.UK. https://www.gov.uk/guidance/clostridium-perfringens.

[207] Cheng J., Hu J., Geng F., Nie S. (2022). Bacteroides Utilization for Dietary Polysaccharides and Their Beneficial Effects on Gut Health. Food Sci. Hum. Wellness.

[208] Hofstad T. (2006). The Genus Fusobacterium. Prokaryotes.

[209] Fusco W., Lorenzo M.B., Cintoni M., Porcari S., Rinninella E., Kaitsas F., Lener E., Mele M.C., Gasbarrini A., Collado M.C. (2023). Short-Chain Fatty-Acid-Producing Bacteria: Key Components of the Human Gut Microbiota. Nutrients.

[210] Karačić A., Dikarlo P., Dorst I., Renko I., Pršo A.-M.L. (2024). The Gut Microbiome Without Any Plant Food? A Case Study on the Gut Microbiome of a Healthy Carnivore. Microbiota Host.

[211] McInerney J.O. (2016). Evolution: A Four Billion Year Old Metabolism. Nat. Microbiol..

[212] Jie Z., Xia H., Zhong S.-L., Feng Q., Li S., Liang S., Zhong H., Liu Z., Gao Y., Zhao H. (2017). The Gut Microbiome in Atherosclerotic Cardiovascular Disease. Nat. Commun..

[213] Muegge B.D., Kuczynski J., Knights D., Clemente J.C., González A., Fontana L., Henrissat B., Knight R., Gordon J.I. (2011). Diet Drives Convergence in Gut Microbiome Functions Across Mammalian Phylogeny and Within Humans. Science.

[214] Betancur-Murillo C.L., Aguilar-Marín S.B., Jovel J. (2022). Prevotella: A Key Player in Ruminal Metabolism. Microorganisms.

[215] Berry D. (2016). The Emerging View of *Firmicutes* as Key Fibre Degraders in the Human Gut. Environ. Microbiol..

[216] Fujimori S. (2021). Humans Have Intestinal Bacteria That Degrade the Plant Cell Walls in Herbivores. World J. Gastroenterol..

[217] Tardiolo G., La Fauci D., Riggio V., Daghio M., Di Salvo E., Zumbo A., Sutera A.M. (2025). Gut Microbiota of Ruminants and Monogastric Livestock: An Overview. Animals.

[218] Mafra D., Borges N.A., Baptista B.G., Martins L.F., Borland G., Shiels P.G., Stenvinkel P. (2024). What Can the Gut Microbiota of Animals Teach Us about the Relationship between Nutrition and Burden of Lifestyle Diseases?. Nutrients.

[219] Zhang P. (2022). Influence of Foods and Nutrition on the Gut Microbiome and Implications for Intestinal Health. Int. J. Mol. Sci..

[220] Desai M.S., Seekatz A.M., Koropatkin N.M., Kamada N., Hickey C.A., Wolter M., Pudlo N.A., Kitamoto S., Terrapon N., Muller A. (2016). A Dietary Fiber-Deprived Gut Microbiota Degrades the Colonic Mucus Barrier and Enhances Pathogen Susceptibility. Cell.

[221] McRorie J.W., McKeown N.M. (2017). Understanding the Physics of Functional Fibers in the Gastrointestinal Tract: An Evidence-Based Approach to Resolving Enduring Misconceptions about Insoluble and Soluble Fiber. J. Acad. Nutr. Diet..

[222] Makki K., Deehan E.C., Walter J., Bäckhed F. (2018). The Impact of Dietary Fiber on Gut Microbiota in Host Health and Disease. Cell Host Microbe.

[223] DeGruttola A.K., Low D., Mizoguchi A., Mizoguchi E. (2016). Current Understanding of Dysbiosis in Disease in Human and Animal Models. Inflamm. Bowel Dis..

[224] LeBlanc J.G., Milani C., de Giori G.S., Sesma F., van Sinderen D., Ventura M. (2013). Bacteria as Vitamin Suppliers to Their Host: A Gut Microbiota Perspective. Curr. Opin. Biotechnol..

[225] Turnbaugh P.J., Ley R.E., Mahowald M.A., Magrini V., Mardis E.R., Gordon J.I. (2006). An Obesity-Associated Gut Microbiome with Increased Capacity for Energy Harvest. Nature.

[226] Rahman S., Hossain K.S., Das S., Kundu S., Adegoke E.O., Rahman A., Hannan A., Uddin J., Pang M.-G. (2021). Role of Insulin in Health and Disease: An Update. Int. J. Mol. Sci..

[227] Yanagisawa Y. (2023). How Dietary Amino Acids and High Protein Diets Influence Insulin Secretion. Physiol. Rep..

[228] Torres N., Noriega L., Tovar A.R. (2009). Chapter 9 Nutrient Modulation of Insulin Secretion. Vitam. Horm..

[229] Kalogeropoulou D., LaFave L., Schweim K., Gannon M.C., Nuttall F.Q. (2008). Leucine, When Ingested with Glucose, Synergistically Stimulates Insulin Secretion and Lowers Blood Glucose. Metabolism.

[230] Krause M.S., McClenaghan N.H., Flatt P.R., Bittencourt P.I.H.d., Murphy C., Newsholme P. (2011). l-Arginine is Essential for Pancreatic β-Cell Functional Integrity, Metabolism and Defense from Inflammatory Challenge. J. Endocrinol..

[231] Halperin F., Mezza T., Li P., Shirakawa J., Kulkarni R.N., Goldfine A.B. (2022). Insulin Regulates Arginine-Stimulated Insulin Secretion in Humans. Metabolism.

[232] Brand-Miller J.C., Griffin H.J., Colagiuri S. (2011). The Carnivore Connection Hypothesis: Revisited. J. Obes..

[233] Manninen A.H. (2004). Metabolic Effects of the Very-Low-Carbohydrate Diets: Misunderstood “Villains” of Human Metabolism. J. Int. Soc. Sports Nutr..

[234] Zelber-Sagi S., Ivancovsky-Wajcman D., Isakov N.F., Webb M., Orenstein D., Shibolet O., Kariv R. (2018). High Red and Processed meat Consumption is Associated with Non-Alcoholic Fatty Liver Disease and Insulin Resistance. J. Hepatol..

[235] Kim Y., Keogh J.B., Clifton P.M. (2017). Consumption of Red and Processed Meat and Refined Grains for 4 Weeks Decreases Insulin Sensitivity in Insulin-Resistant adults: A Randomized Crossover Study. Metabolism.

[236] Mendoza-Herrera K., Florio A.A., Moore M., Marrero A., Tamez M., Bhupathiraju S.N., Mattei J. (2021). The Leptin System and Diet: A Mini Review of the Current Evidence. Front. Endocrinol..

[237] Perakakis N., Mantzoros C.S. (2024). Evidence from Clinical Evidence of Leptin: Current and Future Clinical Applications in Humans. Metabolism.

[238] Weigle D.S., A Breen P., Matthys C.C., Callahan H.S., E Meeuws K., Burden V.R., Purnell J.Q. (2005). A High-Protein Diet Induces Sustained Reductions in Appetite, Ad Libitum Caloric Intake, and Body Weight Despite Compensatory Changes in Diurnal Plasma Leptin and Ghrelin Concentrations. Am. J. Clin. Nutr..

[239] Lv Y., Liang T., Wang G., Li Z. (2018). Ghrelin, a Gastrointestinal Hormone, Regulates Energy Balance and Lipid Metabolism. Biosci. Rep..

[240] Torres J.M., Arias-Gastelum M., Almazán A.M., Verdezoto-Alvarado A., Grijalva-Castro M., Martínez G., Ochoa-Nogales B., Vega-López S. (2022). Associations of Diet Patterns and Cardiometabolic Disease Risk Factors Among Mexican Women in the Sonora-Arizona Transborder Region. Curr. Dev. Nutr..

[241] Ewy M.W., Patel A., Abdelmagid M.G., Mohamed Elfadil O., Bonnes S.L., Salonen B.R., Hurt R.T., Mundi M.S. (2022). Plant-Based Diet: Is It as Good as an Animal-Based Diet When It Comes to Protein?. Curr. Nutr. Rep..

[242] Zhang J., Zheng Y., Martens L., Pfeiffer A.F.H. (2023). The Regulation and Secretion of Glucagon in Response to Nutrient Composition: Unraveling Their Intricate Mechanisms. Nutrients.

[243] Halton T.L., Hu F.B. (2004). The Effects of High Protein Diets on Thermogenesis, Satiety and Weight Loss: A Critical Review. J. Am. Coll. Nutr..

[244] Guarneiri L.L., Adams C.G., Garcia-Jackson B., Koecher K., Wilcox M.L., Maki K.C. (2024). Effects of Varying Protein Amounts and Types on Diet-Induced Thermogenesis: A Systematic Review and Meta-Analysis. Adv. Nutr. Int. Rev. J..

[245] Nunes E.A., Colenso-Semple L., McKellar S.R., Yau T., Ali M.U., Fitzpatrick-Lewis D., Sherifali D., Gaudichon C., Tomé D., Atherton P.J. (2022). Systematic Review and Meta-Analysis of Protein Intake to Support Muscle Mass and Function in Healthy Adults. J. Cachex-Sarcopenia Muscle.

[246] Hernández-Alonso P., Salas-Salvadó J., Ruiz-Canela M., Corella D., Estruch R., Fitó M., Arós F., Gómez-Gracia E., Fiol M., Lapetra J. (2016). High Dietary Protein Intake is Associated with an Increased Body Weight and Total Death Risk. Clin. Nutr..

[247] Pesta D.H., Samuel V.T. (2014). A High-Protein Diet for Reducing Body fat: Mechanisms and Possible Caveats. Nutr. Metab..

[248] Alisson-Silva F., Kawanishi K., Varki A. (2016). Human Risk of Diseases Associated with Red Meat Intake: Analysis of Current Theories and Proposed Role for Metabolic Incorporation of a Non-Human Sialic Acid. Mol. Asp. Med..

[249] Domínguez-Rodrigo M., Courtenay L.A., Cobo-Sánchez L., Baquedano E., Mabulla A. (2022). A Case of Hominin Scavenging 1.84 Million Years Ago from Olduvai Gorge (Tanzania). Ann. N. Y. Acad. Sci..

[250] Rodríguez J., Hölzchen E., Caso-Alonso A.I., Berndt J.O., Hertler C., Timm I.J., Mateos A. (2023). Computer Simulation of Scavenging by Hominins and Giant Hyenas in the Late Early Pleistocene. Sci. Rep..

[251] Szilágyi A., Kovács V.P., Czárán T., Szathmáry E. (2023). Evolutionary Ecology of Language Origins Through Confrontational Scavenging. Philos. Trans. R. Soc. B Biol. Sci..

[252] Pobiner B.L. (2015). New Actualistic Data on the Ecology and Energetics of Hominin Scavenging Opportunities. J. Hum. Evol..

[253] Marean C.W. (1998). A Critique of the Evidence for Scavenging by Neandertals and Early Modern Humans: New Data from Kobeh Cave (Zagros Mountains, Iran) and Die Kelders Cave 1 Layer 10 (South Africa). J. Hum. Evol..

[254] Carlson K.J., Pickering T.R. (2003). Intrinsic Qualities of Primate Bones as Predictors of skeletal Element Representation in Modern and Fossil Carnivore Feeding Assemblages. J. Hum. Evol..

[255] Cobo-Sánchez L., Pizarro-Monzo M., Cifuentes-Alcobendas G., García B.J., Beltrán N.A., Courtenay L.A., Mabulla A., Baquedano E., Domínguez-Rodrigo M. (2022). Computer Vision Supports Primary Access to Meat by Early Homo 1.84 Million Years ago. PeerJ.

[256] Hanon R., Patou-Mathis M., Péan S., Prat S., Cohen B.F., Steininger C. (2022). Early Pleistocene Hominin Subsistence Behaviors in South Africa: Evidence from the Hominin-Bearing Deposit of Cooper’s D (Bloubank Valley, South Africa). J. Hum. Evol..

[257] Matsumoto T. (2019). Opportunistic Feeding Strategy in wild Immature Chimpanzees: Implications for Children as Active Foragers in Human Evolution. J. Hum. Evol..

[258] Matsumoto T. (2017). Developmental Changes in Feeding Behaviors of Infant Chimpanzees at Mahale, Tanzania: Implications for Nutritional Independence Long Before Cessation of Nipple Contact. Am. J. Phys. Anthr..

[259] Watts D.P. (2008). Scavenging by Chimpanzees at Ngogo and the Relevance of Chimpanzee Scavenging to Early Hominin Behavioral Ecology. J. Hum. Evol..

[260] Alger I., Dridi S., Stieglitz J., Wilson M.L. (2023). The Evolution of Early Hominin Food Production and Sharing. Proc. Natl. Acad. Sci. USA.

[261] Lüdecke T., Kullmer O., Wacker U., Sandrock O., Fiebig J., Schrenk F., Mulch A. (2018). Dietary Versatility of Early Pleistocene Hominins. Proc. Natl. Acad. Sci. USA.

[262] Quinn R.L., Lepre C.J. (2021). Contracting eastern African C4 Grasslands During the Extinction of Paranthropus boisei. Sci. Rep..

[263] Swedell L., Plummer T. (2019). Social Evolution in Plio-Pleistocene Hominins: Insights from Hamadryas Baboons and Paleoecology. J. Hum. Evol..

[264] Ungar P.S. (2011). Dental Evidence for the Diets of Plio-Pleistocene Hominins. Am. J. Phys. Anthr..

[265] Drummond-Clarke R.C. (2023). Bringing trees Back into the Human Evolutionary Story: Recent Evidence from Extant Great apes. Commun. Integr. Biol..

[266] Romero M.J.R.H., Ungar P.S., Fried D., Lippert F., Zero D.T., Zunt S., Eckert G.J., Gossweiler A.G., Elkington-Stauss D.J., Tamayo-Cabeza G. (2023). Exploratory Analysis of Objective Outcome Measures for the Clinical Assessment of Erosive Tooth Wear. Diagnostics.

[267] Berthaume M.A., Kupczik K. (2021). Molar biomechanical function in South African hominins *Australopithecus africanus* and *Paranthropus robustus*. Interface Focus.

[268] Domínguez-Rodrigo M., Baquedano E., Organista E., Cobo-Sánchez L., Mabulla A., Maskara V., Gidna A., Pizarro-Monzo M., Aramendi J., Galán A.B. (2021). Early Pleistocene Faunivorous Hominins Were Not Kleptoparasitic, and This Impacted the Evolution of Human Anatomy and Socio-Ecology. Sci. Rep..

[269] Borrero L.A. (2023). The Ephemeral Record: The Role of Opportunistic Animal Resources in the Archaeology of Pampa and Patagonia. Proc. Natl. Acad. Sci. USA.

[270] Palmqvist P., Martínez-Navarro B., Pérez-Claros J.A., Torregrosa V., Figueirido B., Jiménez-Arenas J.M., Espigares M.P., Ros-Montoya S., De Renzi M. (2011). The Giant Hyena Pachycrocuta Brevirostris: Modelling the Bone-Cracking Behavior of an Extinct Carnivore. Quat. Int..

[271] Espigares M.P., Palmqvist P., Rodríguez-Ruiz M.D., Ros-Montoya S., Pérez-Ramos A., Rodríguez-Gómez G., Guerra-Merchán A., García-Aguilar J.M., Granados A., Campaña I. (2023). Sharing Food with Hyenas: A Latrine of Pachycrocuta brevirostris in the Early Pleistocene Assemblage of Fuente Nueva-3 (Orce, Baza Basin, SE Spain). Archaeol. Anthr. Sci..

[272] Parkinson J.A., Plummer T., Hartstone-Rose A. (2015). Characterizing Felid Tooth Marking and Gross Bone Damage Patterns Using GIS Image Analysis: An Experimental Feeding Study with Large Felids. J. Hum. Evol..

[273] Domínguez-Rodrigo M. (1999). Flesh Availability and Bone Modifications in Carcasses Consumed by Lions: Palaeoecological Relevance in Hominid Foraging Patterns. Palaeogeogr. Palaeoclim. Palaeoecol..

[274] Schwingshackl L., Hoffmann G., Sands J.M. (2014). Comparison of High vs. Normal/Low Protein Diets on Renal Function in Subjects without Chronic Kidney Disease: A Systematic Review and Meta-Analysis. PLOS ONE.

[275] Christ A., Lauterbach M., Latz E. (2019). Western Diet and the Immune System: An Inflammatory Connection. Immunity.

[276] Khonsary S. (2017). Guyton and Hall: Textbook of Medical Physiology. Surg. Neurol. Int..

[277] Nelson D.L., Cox M.M. (2017). Lehninger Principles of Biochemistry.

[278] Frank H., Graf J., Amann-Gassner U., Bratke R., Daniel H., Heemann U., Hauner H. (2009). Effect of Short-Term high-Protein Compared with Normal-Protein Diets on Renal Hemodynamics and Associated Variables in Healthy Young Men. Am. J. Clin. Nutr..

[279] Friedman A.N., Ogden L.G., Foster G.D., Klein S., Stein R., Miller B., Hill J.O., Brill C., Bailer B., Rosenbaum D.R. (2012). Comparative Effects of Low-Carbohydrate High-Protein Versus Low-Fat Diets on the Kidney. Clin. J. Am. Soc. Nephrol..

[280] Kamper A.-L., Strandgaard S. (2017). Long-Term Effects of High-Protein Diets on Renal Function. Annu. Rev. Nutr..

[281] Kalantar-Zadeh K., Fouque D., Ingelfinger J.R. (2017). Nutritional Management of Chronic Kidney Disease. N. Engl. J. Med..

[282] Ko G.-J., Rhee C.M., Kalantar-Zadeh K., Joshi S. (2020). The Effects of High-Protein Diets on Kidney Health and Longevity. J. Am. Soc. Nephrol..

[283] Bröer S. (2023). Intestinal Amino Acid Transport and Metabolic Health. Annu. Rev. Nutr..

[284] Gorissen S.H.M., Crombag J.J.R., Senden J.M.G., Waterval W.A.H., Bierau J., Verdijk L.B., van Loon L.J.C. (2018). Protein Content and Amino Acid Composition of Commercially Available Plant-Based Protein Isolates. Amino Acids.

[285] van Vliet S., A Burd N., van Loon L.J. (2015). The Skeletal Muscle Anabolic Response to Plant- Versus Animal-Based Protein Consumption. J. Nutr..

[286] Kashyap S., Shivakumar N., Varkey A., Duraisamy R., Thomas T., Preston T., Devi S., Kurpad A.V. (2018). Ileal Digestibility of Intrinsically Labeled Hen’s Egg and Meat Protein Determined with the Dual Stable Isotope Tracer Method in Indian Adults. Am. J. Clin. Nutr..

[287] Gilani G.S., Xiao C.W., Cockell K.A. (2012). Impact of Antinutritional Factors in Food Proteins on the Digestibility of Protein and the Bioavailability of Amino Acids and on Protein Quality. Br. J. Nutr..

[288] Gorissen S.H., Horstman A.M., Franssen R., Crombag J.J., Langer H., Bierau J., Respondek F., van Loon L.J. (2016). Ingestion of Wheat Protein Increases In Vivo Muscle Protein Synthesis Rates in Healthy Older Men in a Randomized Trial. J. Nutr..

[289] Boirie Y., Dangin M., Gachon P., Vasson M.-P., Maubois J.-L., Beaufrère B. (1997). Slow and Fast Dietary Proteins Differently Modulate Postprandial Protein Accretion. Proc. Natl. Acad. Sci. USA.

[290] O’hEarn A. (2020). Can a Carnivore diet Provide all Essential Nutrients?. Curr. Opin. Endocrinol. Diabetes.

[291] Bolla A.M., Caretto A., Laurenzi A., Scavini M., Piemonti L. (2019). Low-Carb and Ketogenic Diets in Type 1 and Type 2 Diabetes. Nutrients.

[292] Dyńka D., Kowalcze K., Charuta A., Paziewska A. (2023). The Ketogenic Diet and Cardiovascular Diseases. Nutrients.

[293] Wedekind R., Kiss A., Keski-Rahkonen P., Viallon V., A Rothwell J., Cross A.J., Rostgaard-Hansen A.L., Sandanger T.M., Jakszyn P., A Schmidt J. (2020). A Metabolomic Study of Red and Processed Meat Intake and Acylcarnitine Concentrations in Human Urine and Blood. Am. J. Clin. Nutr..

[294] Bagheri M., Willett W., Townsend M.K., Kraft P., Ivey K.L., Rimm E.B., Wilson K.M., Costenbader K.H., Karlson E.W., Poole E.M. (2020). A Lipid-Related Metabolomic Pattern of Diet Quality. Am. J. Clin. Nutr..

[295] Cahill G.F. (2006). Fuel Metabolism in Starvation. Annu. Rev. Nutr..

[296] Puchalska P., Crawford P.A. (2017). Multi-dimensional Roles of Ketone Bodies in Fuel Metabolism, Signaling, and Therapeutics. Cell Metab..

[297] Bedi K.C., Snyder N.W., Brandimarto J., Aziz M., Mesaros C., Worth A.J., Wang L.L., Javaheri A., Blair I.A., Margulies K.B. (2016). Evidence for Intramyocardial Disruption of Lipid Metabolism and Increased Myocardial Ketone Utilization in Advanced Human Heart Failure. Circulation.

[298] Aubert G., Martin O.J., Horton J.L., Lai L., Vega R.B., Leone T.C., Koves T., Gardell S.J., Krüger M., Hoppel C.L. (2016). The Failing Heart Relies on Ketone Bodies as a Fuel. Circulation.

[299] Bertero E., Sequeira V., Maack C. (2018). Hungry Hearts. Circ. Hear. Fail..

[300] Cox P.J., Kirk T., Ashmore T., Willerton K., Evans R., Smith A., Murray A.J., Stubbs B., West J., McLure S.W. (2016). Nutritional Ketosis Alters Fuel Preference and Thereby Endurance Performance in Athletes. Cell Metab..

[301] Poffé C., Hespel P. (2020). Ketone Bodies: Beyond Their Role as a Potential Energy Substrate in Exercise. J. Physiol..

[302] Veneman T., Mitrakou A., Mokan M., Cryer P., Gerich J. (1994). Effect of Hyperketonemia and Hyperlacticacidemia on Symptoms, Cognitive Dysfunction, and Counterregulatory Hormone Responses During Hypoglycemia in Normal Humans. Diabetes.

[303] Edmond J. (1992). Energy metabolism in developing brain cells. Can. J. Physiol. Pharmacol..

[304] Wentz A.E., D’aVignon D.A., Weber M.L., Cotter D.G., Doherty J.M., Kerns R., Nagarajan R., Reddy N., Sambandam N., Crawford P.A. (2010). Adaptation of Myocardial Substrate Metabolism to a Ketogenic Nutrient Environment. J. Biol. Chem..

[305] Sperry J., Condro M.C., Guo L., Braas D., Vanderveer-Harris N., Kim K.K., Pope W.B., Divakaruni A.S., Lai A., Christofk H. (2020). Glioblastoma Utilizes Fatty Acids and Ketone Bodies for Growth Allowing Progression during Ketogenic Diet Therapy. iScience.

[306] Sato K., Kashiwaya Y., A Keon C., Tsuchiya N., King M.T., Radda G.K., Chance B., Clarke K., Veech R.L. (1995). Insulin, Ketone Bodies, and Mitochondrial Energy Transduction. FASEB J..

[307] Samborska J., Więckowiak P., Stelmaszak K., Stencel K., Ogiegło-Kowalczyk A., Wojtasik M., Głodzik M., Miśkiewicz M., Żak K., Łęcka M. (2024). The Impact of the Ketogenic Diet on the Health of Patients with Alzheimer’s Disease. J. Educ. Health Sport.

[308] Zhu H., Bi D., Zhang Y., Kong C., Du J., Wu X., Wei Q., Qin H. (2022). Ketogenic Diet for Human Diseases: The Underlying Mechanisms and Potential for Clinical Implementations. Signal Transduct. Target. Ther..

[309] Volek J.S., Phinney S.D., Forsythe C.E., Quann E.E., Wood R.J., Puglisi M.J., Kraemer W.J., Bibus D.M., Fernandez M.L., Feinman R.D. (2008). Carbohydrate Restriction has a More Favorable Impact on the Metabolic Syndrome than a Low Fat Diet. Lipids.

[310] Veech R.L. (2004). The therapeutic implications of ketone bodies: The Effects of Ketone Bodies in Pathological Conditions: Ketosis, Ketogenic Diet, Redox States, Insulin Resistance, and Mitochondrial Metabolism. Prostaglandins Leukot. Essent. Fatty Acids.

[311] Paoli A., Rubini A., Volek J.S., Grimaldi K.A. (2013). Beyond Weight Loss: A Review of the Therapeutic Uses of Very-Low-Carbohydrate (Ketogenic) Diets. Eur. J. Clin. Nutr..

[312] Rho J.M., Sankar R. (2008). The Ketogenic Diet in a Pill: Is This Possible?. Epilepsia.

[313] Nakamura K., Tonouchi H., Sasayama A., Ashida K. (2018). A Ketogenic Formula Prevents Tumor Progression and Cancer Cachexia by Attenuating Systemic Inflammation in Colon 26 Tumor-Bearing Mice. Nutrients.

[314] Newman J.C., Verdin E. (2014). Ketone Bodies as Signaling Metabolites. Trends Endocrinol. Metab..

[315] Mardinoglu A., Wu H., Bjornson E., Zhang C., Hakkarainen A., Räsänen S.M., Lee S., Mancina R.M., Bergentall M., Pietiläinen K.H. (2018). An Integrated Understanding of the Rapid Metabolic Benefits of a Carbohydrate-Restricted Diet on Hepatic Steatosis in Humans. Cell Metab..

[316] Serino M., Luche E., Gres S., Baylac A., Bergé M., Cenac C., Waget A., Klopp P., Iacovoni J., Klopp C. (2011). Metabolic Adaptation to a High-Fat Diet is associated with a Change in the Gut Microbiota. Gut.

[317] Roberts M.N., Wallace M.A., Tomilov A.A., Zhou Z., Marcotte G.R., Tran D., Perez G., Gutierrez-Casado E., Koike S., Knotts T.A. (2017). A Ketogenic Diet Extends Longevity and Healthspan in Adult Mice. Cell Metab..

[318] Phinney S., Bistrian B., Evans W., Gervino E., Blackburn G. (1983). The Human Metabolic Response to Chronic Ketosis Without Caloric Restriction: Preservation of Submaximal Exercise Capability with Reduced Carbohydrate Oxidation. Metabolism.

[319] Stubbs B.J., Cox P.J., Evans R.D., Santer P., Miller J.J., Faull O.K., Magor-Elliott S., Hiyama S., Stirling M., Clarke K. (2017). On the Metabolism of Exogenous Ketones in Humans. Front. Physiol..

[320] Shimazu T., Hirschey M.D., Newman J., He W., Shirakawa K., Le Moan N., Grueter C.A., Lim H., Saunders L.R., Stevens R.D. (2013). Suppression of Oxidative Stress by β-Hydroxybutyrate, an Endogenous Histone Deacetylase Inhibitor. Science.

[321] Sumithran P., Proietto J. (2008). Ketogenic Diets for Weight Loss: A Review of Their Principles, Safety and Efficacy. Obes. Res. Clin. Pract..

[322] Calcaterra V., Cena H., Sottotetti F., Hruby C., Madini N., Zelaschi N., Zuccotti G. (2023). Low-Calorie Ketogenic Diet: Potential Application in the Treatment of Polycystic Ovary Syndrome in Adolescents. Nutrients.

[323] Verboeket-van de Venne W.P.H.G., Westerterp K.R. (1993). Frequency of Feeding, Weight Reduction and Energy Metabolism. Int. J. Obes..

[324] Gao Z., Yin J., Zhang J., Ward R.E., Martin R.J., Lefevre M., Cefalu W.T., Ye J. (2009). Butyrate Improves Insulin Sensitivity and Increases Energy Expenditure in Mice. Diabetes.

[325] Cotter D.G., Schugar R.C., Crawford P.A. (2013). Ketone Body Metabolism and Cardiovascular Disease. Am. J. Physiol. Circ. Physiol..

[326] Klement R.J. (2022). Was There a Need for High Carbohydrate Content in Neanderthal Diets?. Am. J. Biol. Anthr..

[327] Cordain L., Eaton S., Miller J.B., Mann N., Hill K. (2002). The Paradoxical Nature of Hunter-Gatherer Diets: Meat-Based, Yet Non-atherogenic. Eur. J. Clin. Nutr..

[328] Bragazzi N.L., Del Rio D., A Mayer E., Mena P. (2024). We Are What, When, And How We Eat: The Evolutionary Impact of Dietary Shifts on Physical and Cognitive Development, Health, and Disease. Adv. Nutr. Int. Rev. J..

[329] Williams A.C., Hill L.J. (2017). Meat and Nicotinamide: A Causal Role in Human Evolution, History, and Demographics. Int. J. Tryptophan Res..

[330] You W., Henneberg M. (2016). Meat Consumption Providing a Surplus Energy in Modern Diet Contributes to Obesity Prevalence: An ecological Analysis. BMC Nutr..

[331] Goh Y.Q., Cheam G., Wang Y. (2021). Understanding Choline Bioavailability and Utilization: First Step Toward Personalizing Choline Nutrition. J. Agric. Food Chem..

[332] de Menezes E.V.A., Sampaio H.A.d.C., Carioca A.A.F., Parente N.A., Brito F.O., Moreira T.M.M., de Souza A.C.C., Arruda S.P.M. (2019). Influence of Paleolithic Diet on Anthropometric Markers in Chronic Diseases: Systematic Review and Meta-Analysis. Nutr. J..

[333] Dai L., Schurgers L., Shiels P.G., Stenvinkel P. (2021). A Biomimetic natural Sciences Approach to Understanding the Mechanisms of Ageing in Burden of Lifestyle Diseases. Clin. Sci..

[334] Simopoulos A.P. (1999). Genetic Variation and Nutrition. World Rev. Nutr. Diet.

[335] Singh A., Singh D. (2023). The Paleolithic Diet. Cureus.

[336] Kakodkar S., Mutlu E.A. (2017). Diet as a Therapeutic Option for Adult Inflammatory Bowel Disease. Gastroenterol. Clin. N. Am..

[337] Sohouli M.H., Fatahi S., Lari A., Lotfi M., Seifishahpar M., Găman M.-A., Rahideh S.T., AlBatati S.K., AlHossan A.M., Alkhalifa S.A. (2021). The Effect of Paleolithic Diet on Glucose Metabolism and Lipid Profile Among Patients with Metabolic Disorders: A Systematic Review and Meta-Analysis of Randomized Controlled Trials. Crit. Rev. Food Sci. Nutr..

[338] Jospe M.R., Roy M., Brown R.C., Haszard J.J., Meredith-Jones K., Fangupo L.J., Osborne H., A Fleming E., Taylor R.W. (2020). Intermittent Fasting, Paleolithic, or Mediterranean Diets in the Real World: Exploratory Secondary Analyses of a Weight-Loss Trial That Included Choice of Diet and Exercise. Am. J. Clin. Nutr..

[339] Jamka M., Kulczyński B., Juruć A., Gramza-Michałowska A., Stokes C.S., Walkowiak J. (2020). The Effect of the Paleolithic Diet vs. Healthy Diets on Glucose and Insulin Homeostasis: A Systematic Review and Meta-Analysis of Randomized Controlled Trials. J. Clin. Med..

[340] Anderson K. (2023). Popular Fad Diets: An Evidence-Based Perspective. Prog. Cardiovasc. Dis..

[341] Fontes-Villalba M., Granfeldt Y., Sundquist K., Memon A.A., Hedelius A., Carrera-Bastos P., Jönsson T. (2024). Effects of a Paleolithic Diet Compared to a Diabetes Diet on Leptin Binding Inhibition in Secondary Analysis of a Randomised Cross-Over Study. BMC Endocr. Disord..

[342] Lavie M., Lavie I., Maslovitz S. (2019). Paleolithic Diet During Pregnancy—A Potential Beneficial Effect on Metabolic Indices and Birth Weight. Eur. J. Obstet. Gynecol. Reprod. Biol..

[343] Qiu H., Kan C., Han F., Luo Y., Qu N., Zhang K., Ma Y., Hou N., Wu D., Sun X. (2024). Metagenomic and Metabolomic Analysis Showing the Adverse Risk–Benefit Trade-Off of the Ketogenic Diet. Lipids Health Dis..

[344] Cipryan L., Kosek V., García C.J., Dostal T., Bechynska K., Hajslova J., Hofmann P. (2023). A lipidomic and Metabolomic Signature of a Very Low-Carbohydrate High-Fat Diet and High-Intensity Interval Training: An Additional Analysis of a Randomized Controlled clinical trial. Metabolomics.

[345] Dilmore A.H., Martino C., Neth B.J., West K.A., Zemlin J., Rahman G., Panitchpakdi M., Meehan M.J., Weldon K.C., Blach C. (2023). Effects of a Ketogenic and Low-Fat Diet on the Human Metabolome, Microbiome, and Foodome in Adults at Risk for Alzheimer’s disease. Alzheimer’s Dement..

[346] Lennerz B.S., Mey J.T., Henn O.H., Ludwig D.S. (2021). Behavioral Characteristics and Self-Reported Health Status among 2029 Adults Consuming a “Carnivore Diet”. Curr. Dev. Nutr..

[347] Schwingshackl L., Schwedhelm C., Hoffmann G., Lampousi A.-M., Knüppel S., Iqbal K., Bechthold A., Schlesinger S., Boeing H. (2017). Food Groups and Risk of All-Cause Mortality: A Systematic Review and Meta-Analysis of Prospective Studies. Am. J. Clin. Nutr..

[348] Pitt C.E. (2016). Cutting through the Paleo Hype: The Evidence for the Palaeolithic Diet. Aust. Fam. Physician.

[349] Dahl W.J., Rivero Mendoza D., Lambert J.M. (2020). Diet, Nutrients and the Microbiome. Prog. Mol. Biol. Transl. Sci..

[350] Kiani A.K., Medori M.C., Bonetti G., Aquilanti B., Velluti V., Matera G., Iaconelli A., Stuppia L., Connelly S.T., Herbst K.L. (2022). Modern vision of the Mediterranean diet. J. Prev. Med. Hyg..

[351] Heianza Y., Zhou T., Yuhang C., Huang T., Willett W.C., Hu F.B., Bray G.A., Sacks F.M., Qi L. (2020). Starch Digestion–Related Amylase Genetic Variants, Diet, and Changes in Adiposity: Analyses in Prospective Cohort Studies and a Randomized Dietary Intervention. Diabetes.

[352] Micha R., Michas G., Mozaffarian D. (2012). Unprocessed Red and Processed Meats and Risk of Coronary Artery Disease and Type 2 Diabetes—An Updated Review of the Evidence. Curr. Atheroscler. Rep..

[353] Rohrmann S., Overvad K., Bueno-De-Mesquita H.B., Jakobsen M.U., Egeberg R., Tjønneland A., Nailler L., Boutron-Ruault M.-C., Clavel-Chapelon F., Krogh V. (2013). Meat consumption and mortality—Results from the European Prospective Investigation into Cancer and Nutrition. BMC Med..

[354] Valles-Colomer M., Menni C., Berry S.E., Valdes A.M., Spector T.D., Segata N. (2023). Cardiometabolic Health, Diet and the Gut Microbiome: A Meta-Omics Perspective. Nat. Med..

[355] Zinöcker M.K., Lindseth I.A. (2018). The Western Diet–Microbiome-Host Interaction and Its Role in Metabolic Disease. Nutrients.

[356] Liu R., Hong J., Xu X., Feng Q., Zhang D., Gu Y., Shi J., Zhao S., Liu W., Wang X. (2017). Gut Microbiome and Serum Metabolome Alterations in Obesity and After Weight-Loss Intervention. Nat. Med..

[357] Gentile C.L., Weir T.L. (2018). The Gut Microbiota at the Intersection of Diet and Human Health. Science.

[358] D’almeida K.S.M., Spillere S.R., Zuchinali P., Souza G.C. (2018). Mediterranean Diet and Other Dietary Patterns in Primary Prevention of Heart Failure and Changes in Cardiac Function Markers: A Systematic Review. Nutrients.

[359] Lieberman D.E., Worthington S., Schell L.D., Parkent C.M., Devinsky O., Carmody R.N. (2023). Comparing Measured Dietary Variation Within and Between Tropical Hunter-Gatherer Groups to the Paleo Diet. Am. J. Clin. Nutr..

[360] Konner M., Eaton S.B. (2023). Hunter-Gatherer Diets and Activity as a Model for Health Promotion: Challenges, Responses, and Confirmations. Evol. Anthr..

[361] Veile A. (2018). Hunter-Gatherer Diets and Human Behavioral Evolution. Physiol. Behav..

[362] Pontzer H., Wood B.M. (2021). Effects of Evolution, Ecology, and Economy on Human Diet: Insights from Hunter-Gatherers and Other Small-Scale Societies. Annu. Rev. Nutr..

[363] Olmo B.M.G., Butler M.J., Barrientos R.M. (2021). Evolution of the Human Diet and Its Impact on Gut Microbiota, Immune Responses, and Brain Health. Nutrients.

[364] Genoni A., Lyons-Wall P., Lo J., Devine A. (2016). Cardiovascular, Metabolic Effects and Dietary Composition of Ad-Libitum Paleolithic *vs.* Australian Guide to Healthy Eating Diets: A 4-Week Randomised Trial. Nutrients.

[365] Ghaedi E., Mohammadi M., Mohammadi H., Ramezani-Jolfaie N., Malekzadeh J., Hosseinzadeh M., Salehi-Abargouei A. (2019). Effects of a Paleolithic Diet on Cardiovascular Disease Risk Factors: A Systematic Review and Meta-Analysis of Randomized Controlled Trials. Adv. Nutr. Int. Rev. J..

[366] Vesnina A., Prosekov A., Atuchin V., Minina V., Ponasenko A. (2022). Tackling Atherosclerosis via Selected Nutrition. Int. J. Mol. Sci..

[367] Graff R., Jennings K., Davies N., Carrillo A., Lavoy E., Ryan E., Markofski M. (2021). A Short-Term Paleolithic Dietary Intervention Does Not Alter Adipokines Linked to Adiposity. Int. J. Exerc. Sci..

[368] Shah S., Mahamat-Saleh Y., Hajji-Louati M., Correia E., Oulhote Y., Boutron-Ruault M.-C., Laouali N. (2023). Palaeolithic Diet Score and Risk of Breast Cancer Among Postmenopausal Women Overall and by Hormone Receptor and Histologic Subtypes. Eur. J. Clin. Nutr..

[369] Stomby A., Simonyte K., Mellberg C., Ryberg M., Stimson R.H., Larsson C., Lindahl B., Andrew R., Walker B.R., Olsson T. (2014). Diet-Induced Weight loss Has Chronic Tissue-Specific Effects on Glucocorticoid Metabolism in Overweight Postmenopausal Women. Int. J. Obes..

[370] Fadó R., Molins A., Rojas R., Casals N. (2022). Feeding the Brain: Effect of Nutrients on Cognition, Synaptic Function, and AMPA Receptors. Nutrients.

[371] Fanzo J., Davis C. (2019). Can Diets Be Healthy, Sustainable, and Equitable?. Curr. Obes. Rep..

[372] Schoeneck M., Iggman D. (2021). The Effects of Foods on LDL Cholesterol Levels: A Systematic Review of the Accumulated Evidence from Systematic Reviews and Meta-Analyses of Randomized Controlled Trials. Nutr. Metab. Cardiovasc. Dis..

[373] Zhang X., Sergin I., Evans T.D., Jeong S.-J., Rodriguez-Velez A., Kapoor D., Chen S., Song E., Holloway K.B., Crowley J.R. (2020). High-Protein Diets Increase Cardiovascular Risk by Activating Macrophage mTOR to Suppress Mitophagy. Nat. Metab..

[374] Berding K., Vlckova K., Marx W., Schellekens H., Stanton C., Clarke G., Jacka F., Dinan T.G., Cryan J.F. (2021). Diet and the Microbiota–Gut–Brain Axis: Sowing the Seeds of Good Mental Health. Adv. Nutr. Int. Rev. J..

[375] Kolodziejczyk A.A., Zheng D., Elinav E. (2019). Diet–Microbiota Interactions and Personalized Nutrition. Nat. Rev. Microbiol..

[376] Duncanson K., Williams G., Hoedt E.C., Collins C.E., Keely S., Talley N.J. (2024). Diet-Microbiota Associations in Gastrointestinal Research: A Systematic Review. Gut Microbes.

[377] Tinahones Madueño F.J. (2023). Nutrición y Microbiota. Nutr. Hosp..

